# A Copper-Binding Peptide with Therapeutic Potential against Alzheimer′s Disease: From the Blood–Brain Barrier to Metal Competition

**DOI:** 10.1021/acschemneuro.4c00796

**Published:** 2024-12-26

**Authors:** Victor E. López-Guerrero, Yanahi Posadas, Carolina Sánchez-López, Amanda Smart, Jael Miranda, Kevin Singewald, Yamir Bandala, Eusebio Juaristi, Christophe Den Auwer, Claudia Perez-Cruz, Lorenza González-Mariscal, Glenn Millhauser, Jose Segovia, Liliana Quintanar

**Affiliations:** † Department of Physiology, Biophysics, and Neuroscience, Center for Research and Advanced Studies (Cinvestav), Mexico City 07360, Mexico; ‡ Department of Chemistry, Center for Research and Advanced Studies (Cinvestav), Mexico City 07360, Mexico; § Department of Pharmacology, Center for Research and Advanced Studies (Cinvestav), Mexico City 07360, Mexico; ∥ Center for Research in Aging, Center for Research and Advanced Studies (Cinvestav), Mexico City 14330, Mexico; ⊥ Department of Chemistry and Biochemistry, University of California, Santa Cruz, 1156, Santa Cruz 95064, United States; # El Colegio Nacional, Mexico City 06020, Mexico; ∇ Université Côte d′Azur, CNRS, Institute de Chimie de Nice, Nice 06108, France

**Keywords:** prion protein, copper, Alzheimer′s disease, NMDA receptor, chelation therapy

## Abstract

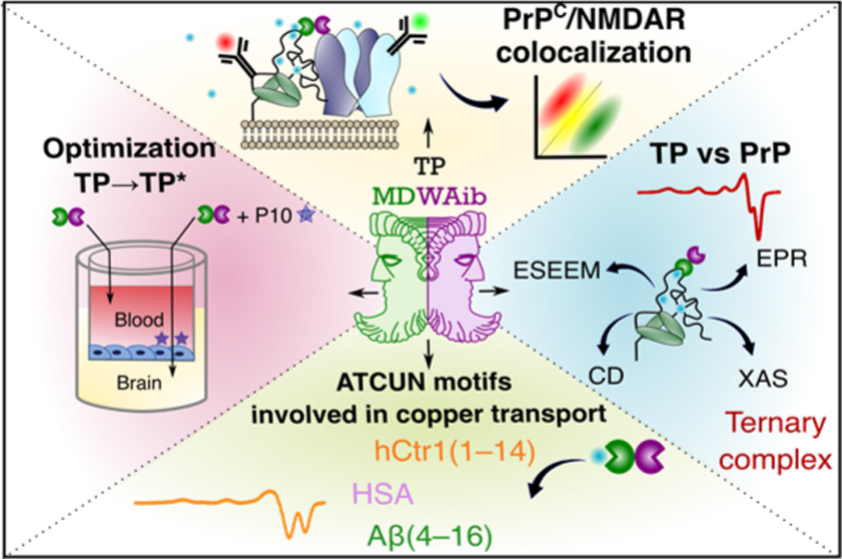

Alzheimer’s disease (AD) is the most common form of dementia worldwide. AD brains are characterized by the accumulation of amyloid-β peptides (Aβ) that bind Cu^2+^ and have been associated with several neurotoxic mechanisms. Although the use of copper chelators to prevent the formation of Cu^2+^-Aβ complexes has been proposed as a therapeutic strategy, recent studies show that copper is an important neuromodulator that is essential for a neuroprotective mechanism mediated by Cu^2+^ binding to the cellular prion protein (PrP^C^). Therefore, in addition to metal selectivity and blood–brain barrier (BBB) permeability, an emerging challenge for copper chelators is to prevent the formation of neurotoxic Cu^2+^-Aβ species without perturbing the neuroprotective Cu^2+^-PrP^C^ interaction. Previously, we reported the design of a tetrapeptide (TP) that withdraws Cu^2+^ from Aβ(1–16) and impacts the Cu^2+^-induced aggregation of Aβ(1–40). In this study, we improved the drug-like properties of TP in a BBB model, evaluated the metal selectivity of the optimized peptide (TP*), and tested its effect on Cu^2+^ coordination to PrP^C^ and proteins involved in copper trafficking, such as copper transporter 1 and albumin. Our results show that changing the stereochemistry of the first residue prevents TP degradation in the BBB model and coadministration of TP with a peptide that increases BBB permeability allows its passage through the BBB model. TP* is highly selective toward Cu^2+^ in the presence of Zn^2+^ ions, transfers Cu^2+^ to copper-trafficking proteins, and forms a ternary TP*-Cu^2+^-PrP species that does not perturb the physiological conformation of PrP and displays only a minor impact in the neuroprotective Cu^2+^-dependent interaction of PrP^C^ with the *N*-methyl-d-aspartate receptor. Overall, these results show that TP* displays desirable features for a copper chelator with therapeutic potential against AD. Moreover, this is the first study that explores the effect of a Cu^2+^ chelator with therapeutic potential for AD on Cu^2+^ coordination to PrP^C^ (an emerging key player in AD pathology), integrating recent knowledge about metalloproteins involved in AD with the design of copper chelators against AD.

## Introduction

Alzheimer′s disease (AD) is a fatal disorder that affects around 35 million people worldwide and lacks an effective treatment.^1^ According to the World Health Organization, AD was the seventh leading cause of death in 2019.^2^ Although AD etiology is not entirely understood, accumulation of amyloid-β peptides (Aβ) in neuritic plaques and copper dyshomeostasis are AD hallmarks.^3−10^ Aβ is a 37 to 42 amino acid Cu^2+^-binding peptide, termed Aβ(1–X), secreted at the synapse after the cleavage of the amyloid precursor protein (APP).^11^ Cu^2+^-Aβ(1–X) complexes produce reactive oxygen species,^12^ and its accumulation in neuritic plaques^5,6^ could trigger copper dyshomeostasis observed in AD patients.^10,13^ Hence, preventing Cu^2+^–Aβ(1–X) interactions using copper chelators has been proposed as a therapeutic strategy against AD.^10,13–15^

Since copper homeostasis is already affected in AD, appropriate copper chelators should not further aggravate this condition by altering Cu^2+^ binding to proteins involved in copper trafficking,^17^ such as the human copper transporter 1 (hCtr1)^18,19^ and the human serum albumin (HSA),^20^ which bind copper with high affinity. While hCtr1 is the main transporter that uptake copper in mammalian cells, HSA is a major copper carrier in the bloodstream.^21−24^ Thus, testing the impact of therapeutic chelators on Cu^2+^ binding to these proteins is critical to understanding its effect in copper homeostasis.

On the other hand, successful design of copper chelators that target Cu^2+^–Aβ(1–X) interactions has been hampered due to the limited understanding of the role of copper ions in the brain. Emerging evidence suggests that copper plays an important role in modulating the activation of *N*-methyl-d-aspartate receptors (NMDARs), mainly by a mechanism that depends on the cellular prion protein (PrP^C^).^25−27^ NMDARs are Ca^2+^-permeable excitatory ionic channels involved in memory and learning processes; nonetheless, its improper modulation results in neurotoxicity.^28−30^ PrP^C^ binds copper ions and regulates the NMDAR in a copper-dependent manner, protecting neurons from calcium overload.^25−27^ Interestingly, Cu^2+^ chelators, such as bathocuproine and Aβ(1–42), disrupt the neuroprotective PrP^C^-NMDAR interaction, increasing neuron susceptibility to excitatory damage.^25^ Thus, although the removal of Cu^2+^ from Cu^2+^-Aβ(1–X) complexes might display a therapeutic benefit for AD, indiscriminate copper chelation might alter physiological copper-dependent processes associated with neurotransmission, such as NMDAR modulation.

**Scheme 1:**
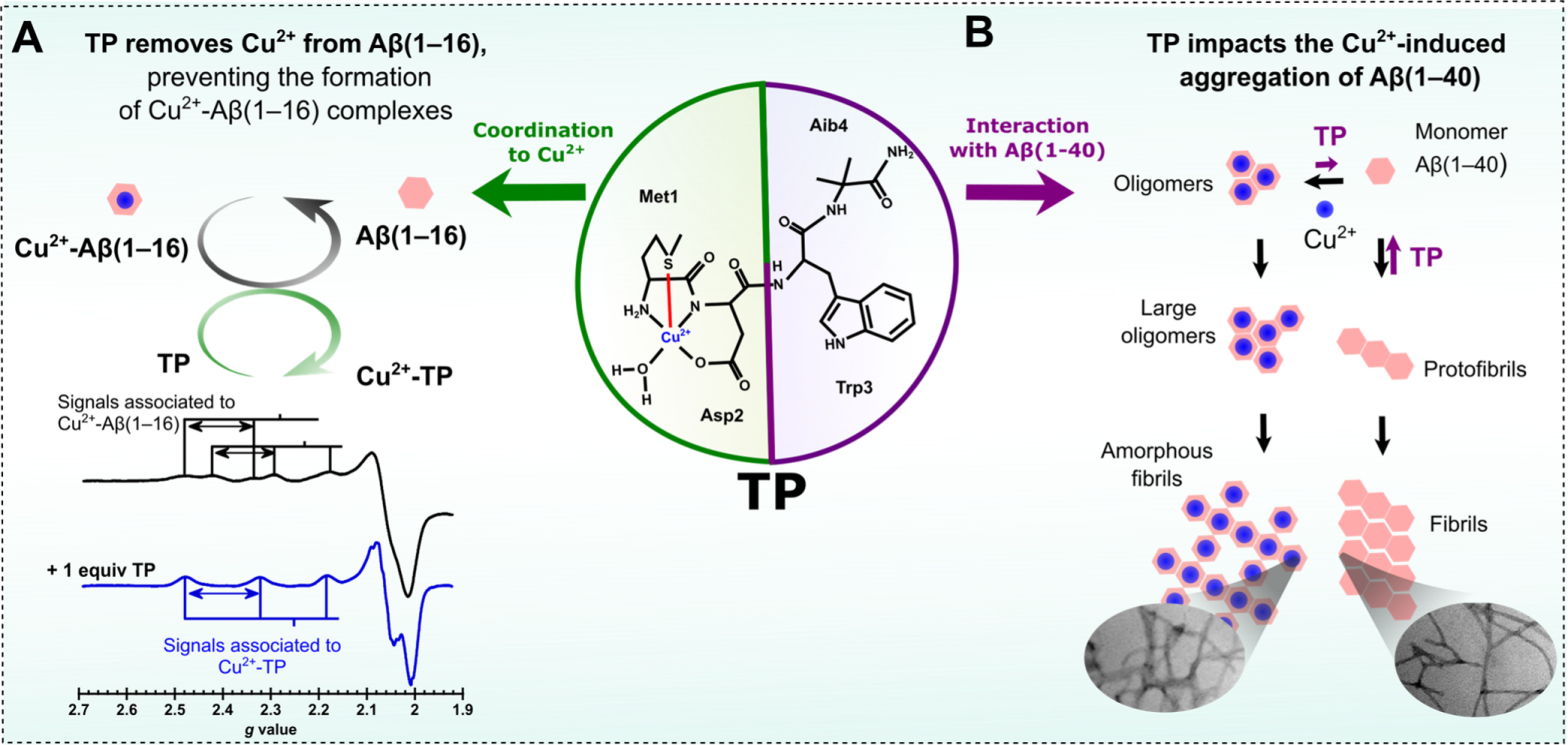
TP Holds Therapeutic Potential against AD TP is a tetrapeptide with the sequence MDdWAib. The MD sequence binds Cu^2+^ with high affinity (A). The hydrophobic dWAib fragment displays β-breaker properties, and it interacts with early intermediate assemblies of Aβ(1–40/42). The presence of both moieties in the same peptide sequence provide bifunctional properties to TP: It removes Cu^2+^ from Aβ(1–16), as demonstrated by EPR (A), and it impacts the Cu^2+^-induced aggregation of Aβ(1–40), inhibiting the formation of large Cu^2+^-bound Aβ(1–40) oligomers (B). These findings support a therapeutic potential for TP against AD. (Figure adapted from (16) ).

**Scheme 2:**
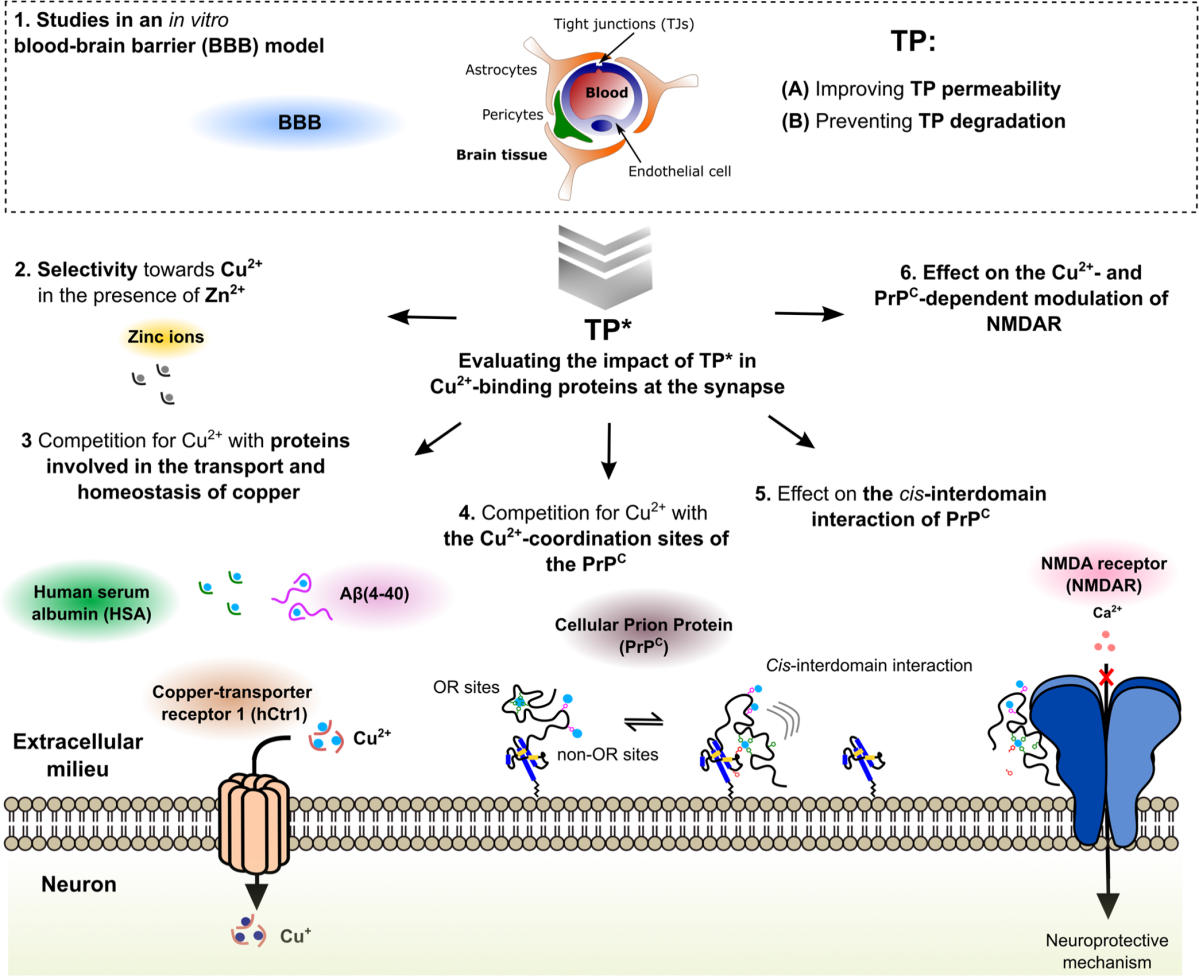
Strategy to Evaluate and Optimize the Drug-like Properties of TP and Evaluate Its Impact on Cu^2+^ Binding to Proteins Involved in Copper Homeostasis and Neuroprotection TP with the sequence MDdWAib was optimized and evaluated using spectroscopic and cell culture assays. This approach includes (1) improving TP permeability in an *in vitro* BBB cellular model and its optimization to prevent degradation; (2) selectivity towards Cu^2+^ in the presence of Zn^2+^ ions; (3) competitions for Cu^2+^ with peptide models of metal-binding sites of proteins associated to the copper transport and homeostasis, such as HSA, hCtr1, and Aβ(4–40); (4) competitions for Cu^2+^ with each of the Cu^2+^-binding sites of the PrP^C^; and (5) its impact on the Cu^2+^-dependent *cis*-interdomain interaction of the PrP; and in (G) the Cu^2+^- and PrP^C^-dependent modulation of NMDARs.

In this context, an ideal chelator to treat AD must remove the metal from Cu^2+^-Aβ(1–X) species without interfering with Cu^2+^ binding to proteins involved in neuroprotection, such as PrP^C^, a metal-binding protein highly abundant in synaptic terminals that coordinates at least 60–80% of copper in synaptosomes.^31,32^ In the past two decades, the coordination chemistry of Cu^2+^-PrP^C^ complexes has been deeply characterized.^33−39^ Cu^2+^ binding to PrP^C^ is highly dynamic: at physiological pH, PrP^C^ can coordinate up to six copper ions at the *N*-terminal region depending on the Cu^2+/^PrP^C^ ratio: up to four ions in a region named octarepeat (OR),^33−35^ consisting of four tandem repeats of the eight-residue sequence (Pro-His-Gly-Gly-Gly-Trp-Gly-Gln), and two additional Cu^2+^ ions at the nonoctarepeat (non-OR) region, which contains the Cu^2+^-anchoring His96 and His111 sites.^36–38^ Although PrP^C^ is one of the most important Cu^2+^-binding proteins in the brain, its functional role is not completely understood. Interestingly, the six His residues that anchored Cu^2+^ at the *N*-terminal of PrP^C^ are required for the Cu^2+^-dependent modulation of NMDARs.^26^ Recent studies have demonstrated that Cu^2+^ binding to PrP^C^ promotes contacts between the *N*- and *C*-terminal domains (*cis*-interdomain interaction).^40^ Moreover, electrophysiological studies suggest that the *cis*-conformation of PrP^C^ is key for its physiologically relevant interaction with different protein partners,^41,42^ such as NMDARs. Hence, evaluating the effect of copper chelators in the Cu^2+^ coordination to PrP^C^ and its metal-induced *cis*-conformation is crucial to characterize its therapeutic potential. We have previously reported the design of a bifunctional tetrapeptide (TP) that can efficiently compete for Cu^2+^ with Aβ(1–16) and it impacts Cu^2+^-induced aggregation of Aβ(1–40) (Scheme 1).^16^ In this study, we have evaluated and improved the drug-like properties of TP (Scheme 2), using an innovative approach that combines a wide range of spectroscopic studies, electron paramagnetic resonance (EPR), electronic circular dichroism (CD), X-ray absorption spectroscopy (XAS), and nuclear magnetic resonance (NMR) in tandem with cell culture assays. In brief, we probed TP for its (1) permeability through the BBB; (2) selectivity toward Cu^2+^ over other relevant metal ions at the synapse, such as Zn^2+^; (3) ability to compete with proteins involved in Cu^2+^ homeostasis; (4) ability to compete with Cu^2+^-binding sites of PrP^C^; impact in the Cu^2+^-induced *cis*-conformation of PrP^C^ (5), and effect on the Cu^2+^-dependent interaction of PrP^C^ with NMDARs (6).

## Results

### Evaluation of TP Permeability in a BBB Model

One of the main obstacles for TP and other peptide-based drugs is the susceptibility to enzymatic degradation and low permeability through the blood–brain barrier (BBB),^43,44^ which is a physical barrier that separates brain tissue from the bloodstream.^45^ The BBB is formed by tight junctions (TJs) present in the endothelial cells of the brain microvasculature. The BBB is surrounded by pericytes and the end feet of astrocytes that provide biochemical support for TJ sealing.^45^ Only molecules with high lipophilicity or with a specific transport mechanism can enter the brain, crossing the plasma membrane of brain capillaries through what is known as the transcellular pathway. The BBB limits the passage of pathogens, immune factors, solutes, and drugs through the paracellular pathway (between endothelial cells) from the blood into the brain.^46,47^ Thus, the evaluation of BBB permeability is a critical step in the design and optimization of drugs that target the brain, such as TP.

#### TP Crosses the BBB When It Is Coadministered with a Peptide That Modulates Tight Junctions

**Figure 1:**
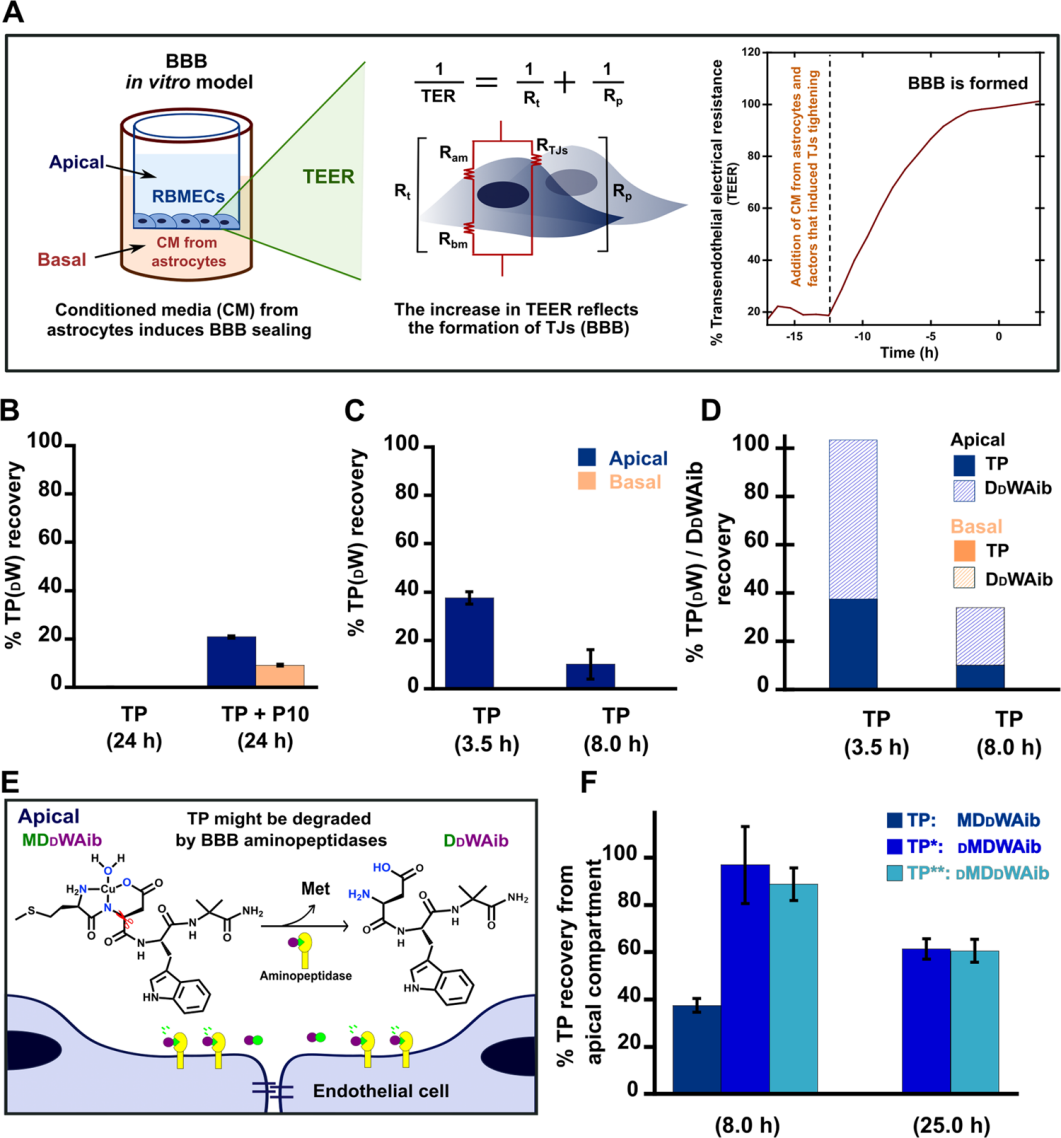
Evaluation of TP permeability in a BBB *in vitro* model. (A) BBB *in vitro* model consists of a primary culture of rat brain microvascular endothelial cells (RBMECs) plated in the upper chamber of an insert with a semipermeable filter set in a multiwell plate, where conditioned media derived from a primary culture of astrocytes bathe the lower compartment. The electrical circuit that describes the movement of electrical charges through a monolayer of endothelial cells is defined by the transendothelial electrical resistance (TEER), which depends on the paracellular resistance (Rp) and the transcellular resistance (Rt). Rt is due to the resistance to the apical (Ram) and basal (Rbm) membranes, while Rp depends on the resistance of tight junctions (R_TJ_). The BBB is formed by the endothelial cells TJs; thus, an increase in TEER is directly associated with the sealing of the BBB. (B) TP recovery from apical and basal compartments of the BBB *in vitro* model, in the absence and presence of P10 after 24 h. (C) TP recovery from both compartments of the BBB *in vitro* model, after 3.5 and 8 h. (D) TP and DdWAib recoveries from apical and basal compartments of the BBB *in vitro* model, at 3.5 and 8 h. (E) Proposed degradation mechanism of MDdWAib to DdWAib in the BBB *in vitro* model. (F) Recovery of TP and its variants TP* and TP** from the apical compartment of the BBB *in vitro* model, at 8 and 25 h.

To assay the permeability of TP through the BBB, we used an *in vitro* BBB model previously described,^48^ where a primary culture of rat brain microvascular endothelial cells (RBMECs) was plated on inserts with semipermeable filters placed in a multiwell plate. In this model (Figure 1A, left panel), the apical compartment contains the RBMECs that *in vivo* would have their apical membranes in contact with the bloodstream, while the basal compartment contains the astrocyte-conditioned medium that bathes through the inset filter the basolateral membrane of RBMECs and induces the BBB formation, simulating the contact of brain endothelial cells with astrocytes in the brain parenchyma. Sealing of the BBB after the addition of astrocyte-conditioned media was demonstrated by continuous measurements of the transendothelial electrical resistance (TEER) (Figure 1A, right panel). Once the RBMECs reached a stable TEER (plateau), TP was added into the apical compartment alone or coadministered with peptide P10, which consists of three amino acids, Lys–Leu–Tyr, selected from the sequence of rotavirus protein VP8 that transiently opens TJs.^49,50^Figure S1 shows that administration of P10, either alone or together with TP, reduces the TEER in comparison with control monolayers and monolayers treated only with TP. To further confirm that P10 opens the BBB and allows the passage of TP to the basal compartment, TP was quantified in the apical and basal compartments using an HPLC method (Figure 1B). Interestingly, TP was not detected in the apical or basal compartments when it was administrated alone. However, TP was recovered from both compartments when it was coadministered with P10 (Figure 1B). These results suggest that TP is degraded in the BBB model and that coadministration with P10 prevents its degradation and allows TP to cross the BBB.

To characterize the degradation of TP in the BBB *in vitro* model, TP was administered in the apical compartment and samples from culture media were taken from the apical and basal compartments after 3.5 and 8 h, confirming that TP is rapidly degraded and cannot cross the BBB without P10 (Figure 1C). A further analysis of the chromatograms of these samples reveals a peak (RT ∼ 5.0 min) with a different retention time from that of TP (RT ∼ 9.5) and of cultured medium components (RTs: ∼ 1.8 and 3.5 min) (Figure S2A). Interestingly, the peak at 5.0 min was identified by mass spectrometry as the fragment DdWAib. This was confirmed by comparison of these retention times and mass spectra to those of the synthesized peptide DdWAib (Figure S2B,C). Hence, DdWAib was quantified in the samples at 3.5 and 8 h (Figure 1D). The sum of recovered TP and DdWAib at 3.5 h yields 100%, indicating that DdWAib is the main degradation product of TP in the BBB model. However, at 8 h, the sum of recovered peptides yields only 34%, suggesting that at longer time periods, there are additional nonidentified degradation products.

#### Presence of a d-Amino Acid in the First Position Is Key to Prevent TP Degradation

Considering the peptidic nature of TP, its degradation to DdWAib might be driven by aminopeptidases, enzymes that recognize and cleave the first or last amino acid in the sequence (Figure 1E).^51^ Hence, to prevent TP degradation, we synthesized two TP variants in which the l-to-d stereochemistry of Met1 was changed, namely, dMDWAib (TP*) and dMDdWAib (TP**) (with d-Met). TP and its d-Met variants were administered into the apical compartment of the BBB *in vitro* model and quantified after 8 and 25 h (Figure 1F). Consistent with the inability of TP to cross the BBB alone, TP and its variants were detected in the apical but not in the basal compartment. On the other hand, both d-Met variants displayed lower degradation rate in comparison with the original TP (Figure 1F), indicating that the presence of a d-amino acid at the beginning of the sequence protects the peptide from degradation, while a d-amino acid in the middle does not. Therefore, in the subsequent studies to evaluate the impact of TP on the Cu^2+^ binding of proteins at the synapse (Scheme 2), we used the optimized sequence TP* with the sequence dMDWAib that is resistant to degradation and preserves the bifunctional properties of the original TP. In fact, TP* displays the same copper coordination properties as TP (Figure S3A–C), and it retains the ability to impact the mechanism of Cu^2+^-induced aggregation of Aβ(1–40) (Figure S3D).

### TP* Binds Preferentially Cu^2+^ in the Presence of Zn^2+^

**Figure 2:**
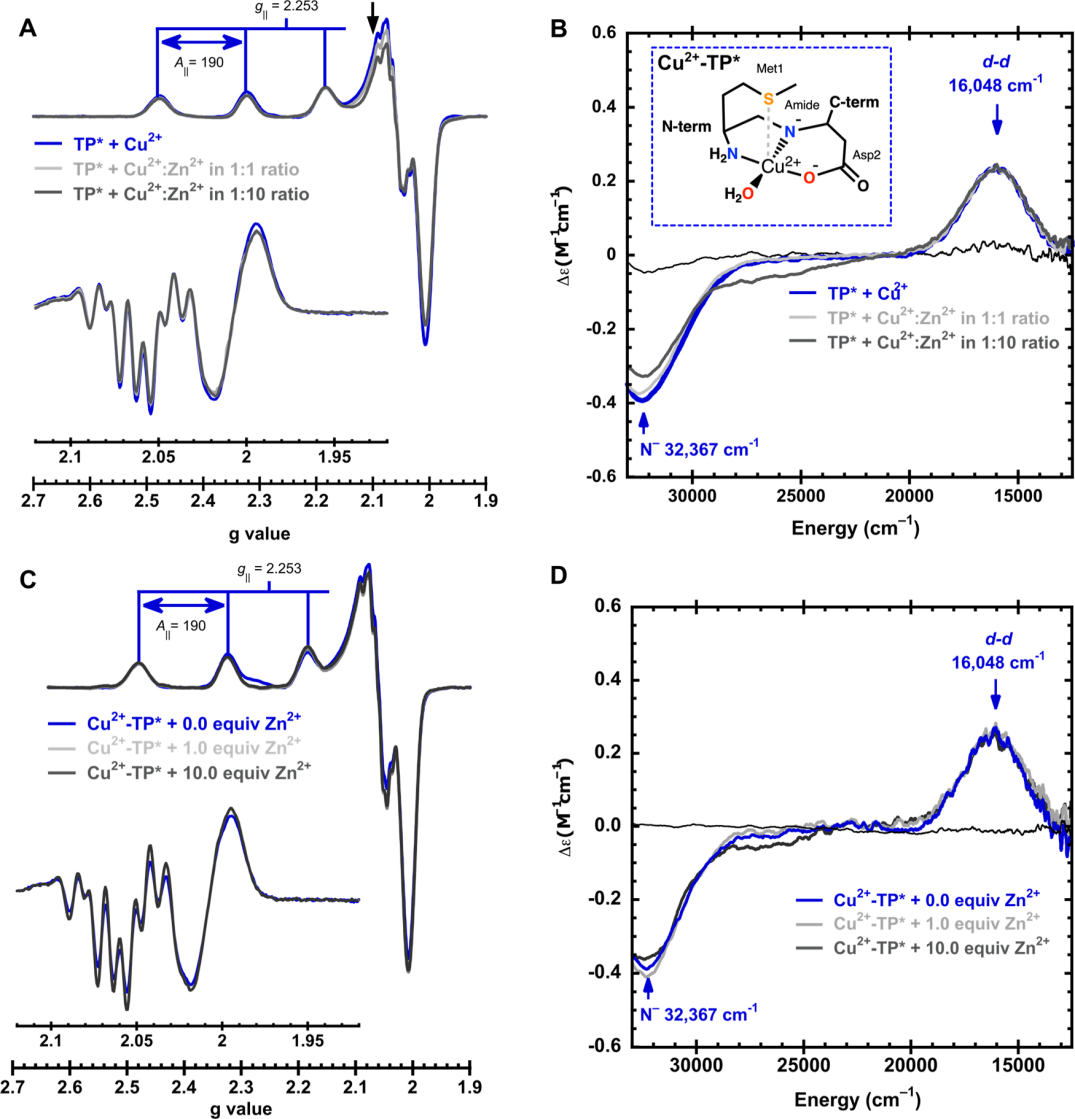
TP* binds preferentially Cu^2+^ ions even in the presence of excess Zn^2+^ ions. EPR (A) and CD (B) spectra of TP* solutions containing 1 equiv of Cu^2+^ (blue spectra) and TP* with 1 equiv of stock solutions containing a mixture of Cu^2+^:Zn^2+^ ions in either 1:1 (light-gray spectra) or 1:10 ratio (dark-gray spectra). In the three conditions, the EPR signals and the nitrogen superhyperfine coupling pattern (inset in part A) are very similar. EPR (C) and CD (D) spectra of the titration of Cu^2+^-TP* with 1 or 10 equiv of Zn^2+^ (light and dark gray, respectively). *A*_∥_ values are given in 1 × 10^–4^ cm^–1^. The inset in (C) shows the second derivative of the EPR spectra, showing very similar nitrogen superhyperfine splittings in the three conditions. A coordination model for the 2N2O or 2N2O1S (participation of the axial S ligand is represented as gray dotted lines) for the Cu^2+^-TP* complex is included in the inset in (B).

Copper is not the only metal ion that acts as a neuromodulator and is involved in AD.^52^ Zn^2+^ accumulates in amyloid plaques and is released to glutamatergic synapses.^5,8,53,54^ Moreover, both metal ions modulate neurotransmission differently, and its dyshomeostasis displays varied toxic pathways.^53−55^ Consequently, metal selectivity is crucial to dissect the effect of copper from zinc ions in AD. Hence, to evaluate the selectivity of optimized TP* toward Cu^2+^ ions in the presence of Zn^2+^ ions, experiments where copper and zinc have equal exposure to TP* were performed. EPR and CD spectroscopies were used to assess the binding of Cu^2+^ to TP*. Samples were prepared starting with a solution of TP* at pH = 7.4, and one chemical equivalent (equiv) of Cu^2+^ was added using stock solutions containing a mixture of Cu^2+^:Zn^2+^ ions in either 1:1 or 1:10 ratio. The EPR spectrum of the Cu^2+^-TP* complex (Figure 2A) displays a *g*_∥_ of 2.253 and an *A*_∥_ of 190 × 10^–4^ cm^–1^ with a characteristic nitrogen superhyperfine coupling pattern (Figure 2A, inset). TP* solutions with a Cu^2+^:Zn^2+^metal mixture in 1:1 ratio or 1:10 display EPR spectra that are identical to those of the Cu^2+^-TP* complex. Moreover, the amount of Cu^2+^-TP* in each sample was determined by EPR spin quantitation, finding that the concentration of the Cu^2+^-TP* complex in the presence of Zn^2+^, in 1:1 or 1:10 ratio, is practically the same (∼99.6 and ∼98.5%, respectively) as compared to the sample without Zn^2+^. Consistently, the CD signals associated with the Cu^2+^-TP* complex are observed even in the presence of a 10-fold Zn^2+^ excess (Figure 2B). Namely, its positive *d–d* band at 16,048 cm^–1^ remains unchanged and the negative ligand-to-metal charge transfer (LMCT) band at 32,367 cm^–1^, assigned to a deprotonated amide to Cu^2+^,^16,56^ displays only minor intensity changes. These results demonstrate that TP* preferentially binds Cu^2+^ ions, even in the presence of excess Zn^2+^ ions. Additionally, to evaluate if Zn^2+^ ions can remove Cu^2+^ from Cu^2+^-TP* species, the Cu^2+^-TP* complex was titrated at physiological pH = 7.4 with increasing amounts of Zn^2+^ followed by EPR (Figure 2C) and CD (Figure 2D). Upon the addition of 1 and 10 equiv of Zn^2+^, the spectroscopic features of the Cu^2+^-TP* complex remain unchanged. Altogether, these results demonstrate that TP* binds Cu^2+^ more tightly than does Zn^2+^.

### TP* Cannot Remove but Transfers Cu^2+^ to Proteins Involved in Copper Homeostasis

**Figure 3:**
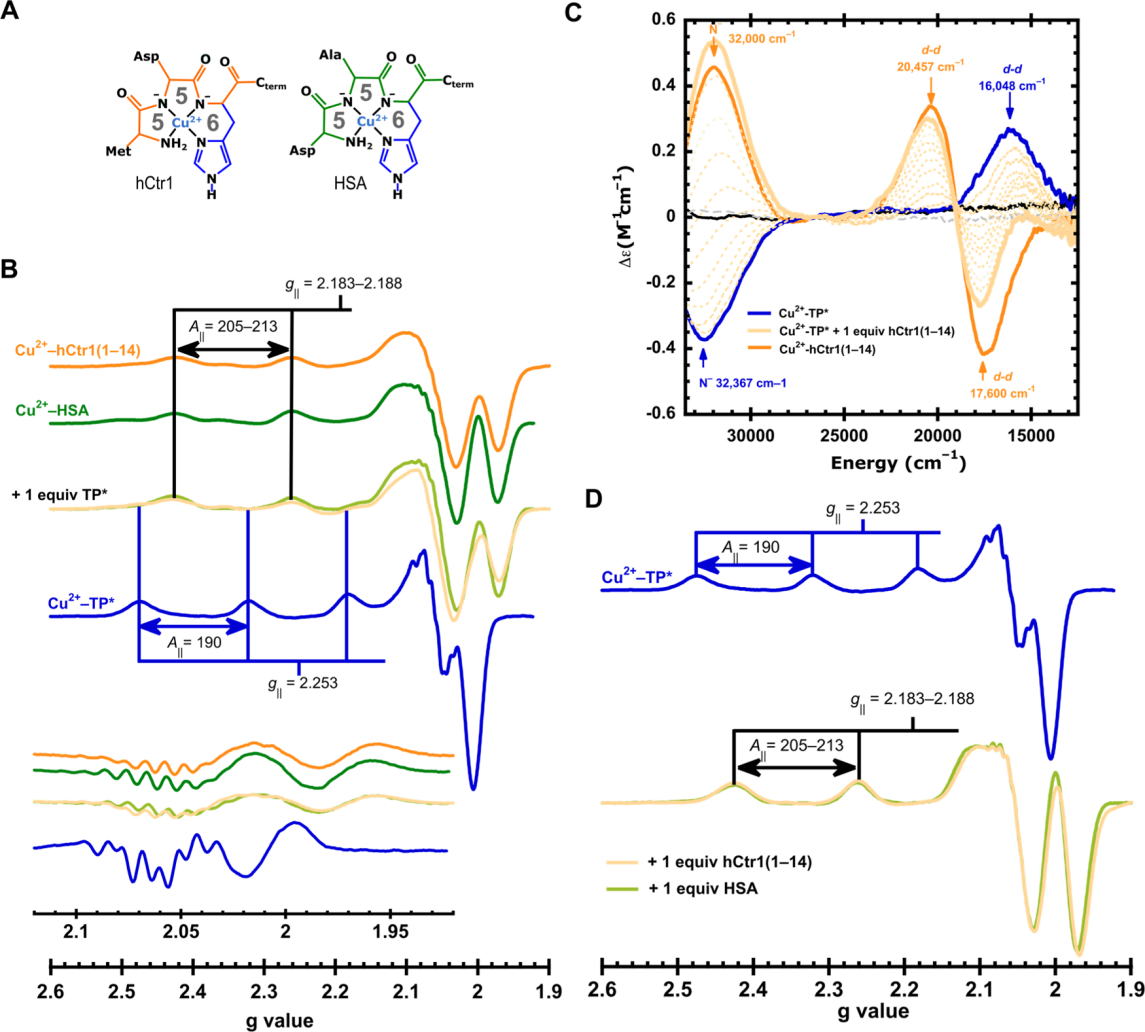
Competition for Cu^2+^ between TP* and proteins involved in copper homeostasis. Coordination modes for Cu^2+^-hCtr1(1–14) (orange) and Cu^2+^-HSA (green) complexes (A). EPR (B) and CD (C) spectra of the Cu^2+^-ATCUN complexes. By EPR (A), Cu^2+^-ATCUN complexes were titrated with 1 equiv of TP* (overlapped spectra in light shades of colors) and Cu^2+^-TP* is included for comparison (blue spectrum); the inset shows the second derivative of the perpendicular region of the spectra. *A*_∥_ values are given in 1 × 10^–4^ cm^–1^. Representative CD spectra of the titration of the Cu^2+^-TP* complex (blue spectrum) with increasing amounts of hCtr1(1–14) (dotted light orange spectra) up to 1 equiv (solid light-orange spectrum) (C), and corresponding EPR spectra of the final point of the titration of Cu^2+^-TP*with hCtr1(1–14) or HSA are shown in (D).

Although selective Cu^2+^ chelators designed for AD must compete for Cu^2+^ with Cu^2+^-Aβ(1-X) species, they should not alter normal copper homeostasis. Henceforth, we next evaluated whether TP* could remove Cu^2+^ from these extracellular proteins or peptides involved in copper transport and homeostasis, such as HSA and hCtr1 proteins. Both proteins contain the typical amino-terminal copper and nickel (ATCUN) binding motif, associated with the *N*-terminal sequence H_2_N-Xaa-Yaa-His-. The His residue in the third position enables high-affinity Cu^2+^ binding through a 4N (H_2_N,N^–^,N^–^,N_His_) coordination sphere, involving the NH_2_ terminal group, two deprotonated backbone amides, and the His imidazole group, resulting in stable (5,5,6)-membered chelate rings (Figure 3A).^57^ Here, the HSA protein and the *N*-terminal peptide model of hCtr1(1–14) were used. Their Cu^2+^ complexes were prepared at pH = 7.4 with 1:1 metal:peptide ratio, yielding the characteristic spectroscopic features of ATCUN complexes, a set of EPR signals in the range of *g*_∥_ = 2.183–2.188 and *A*_∥_ = 205–213 × 10^–4^ cm^–1^ (Figure 3B), and CD spectra with two ligand field transitions, a negative one at the range of 17,650–17,900 cm^–1^ and a positive in the range of 20,520 to 20,900 cm^–1^ and a LMCT band in a range of 31,643–32,000 cm^–1^ that may be associated with N^–^ amide or N_im_π_1_ to Cu^2+^ CT (Figure S4 and Table S3), consistent with previous reports.^57^ These two complexes were confronted with TP*; upon addition of 1 equiv of TP*, their characteristic spectroscopic features remained unaffected, and no signals associated with the Cu^2+^-TP* complex were observed (Figure 3B). This is particularly evident by EPR, where the nitrogen superhyperfine coupling patterns of the ATCUN complexes are maintained in the presence of 1 equiv of TP*, and they are distinct from those observed for the Cu^2+^-TP* complex (Figure 3B, inset). Hence, TP* cannot remove Cu^2+^ from the ATCUN sites of these important metal-trafficking proteins.

Next, to assess if TP* can deliver Cu^2+^ to these ATCUN sites, the Cu^2+^-TP* complex at pH = 7.4 was titrated with hCtr1(1–14) or HSA and monitored by CD and EPR spectroscopies.

A representative titration for hCtr1(1–14) is shown in Figure 3C, while data for HSA are shown in Figure S5 and Table S4. Upon addition of increasing amounts of hCtr1(1–14), the typical CD signals of the Cu^2+^-TP* complex (Figure 3C, blue spectrum) decrease, while *d–d* bands at −17,600 cm^–1^ and +20,457 cm^–1^ and a LMCT band at +32,000 cm^–1^ appear. The final CD spectrum of the titration exhibits signals almost identical to those of the Cu^2+^-hCtr1(1–14) complex (Figure 3C); a similar result was obtained when the Cu^2+^-TP* complex was titrated with HSA (Figure S5, Table S4). Moreover, the EPR spectra of the final points of these titrations yield the characteristic signals of the Cu^2+^-ATCUN sites in hCtr1(1–14) and HSA, while no signals of the Cu^2+^-TP* complex are detected (Figure 3D). These findings show that Cu^2+^-loaded TP* can deliver the metal ion to the ATCUN motif containing proteins involved in copper trafficking.

### TP* Takes Cu^2+^ Away from Aβ(1–16) but Not from Aβ (4–16)

**Figure 4:**
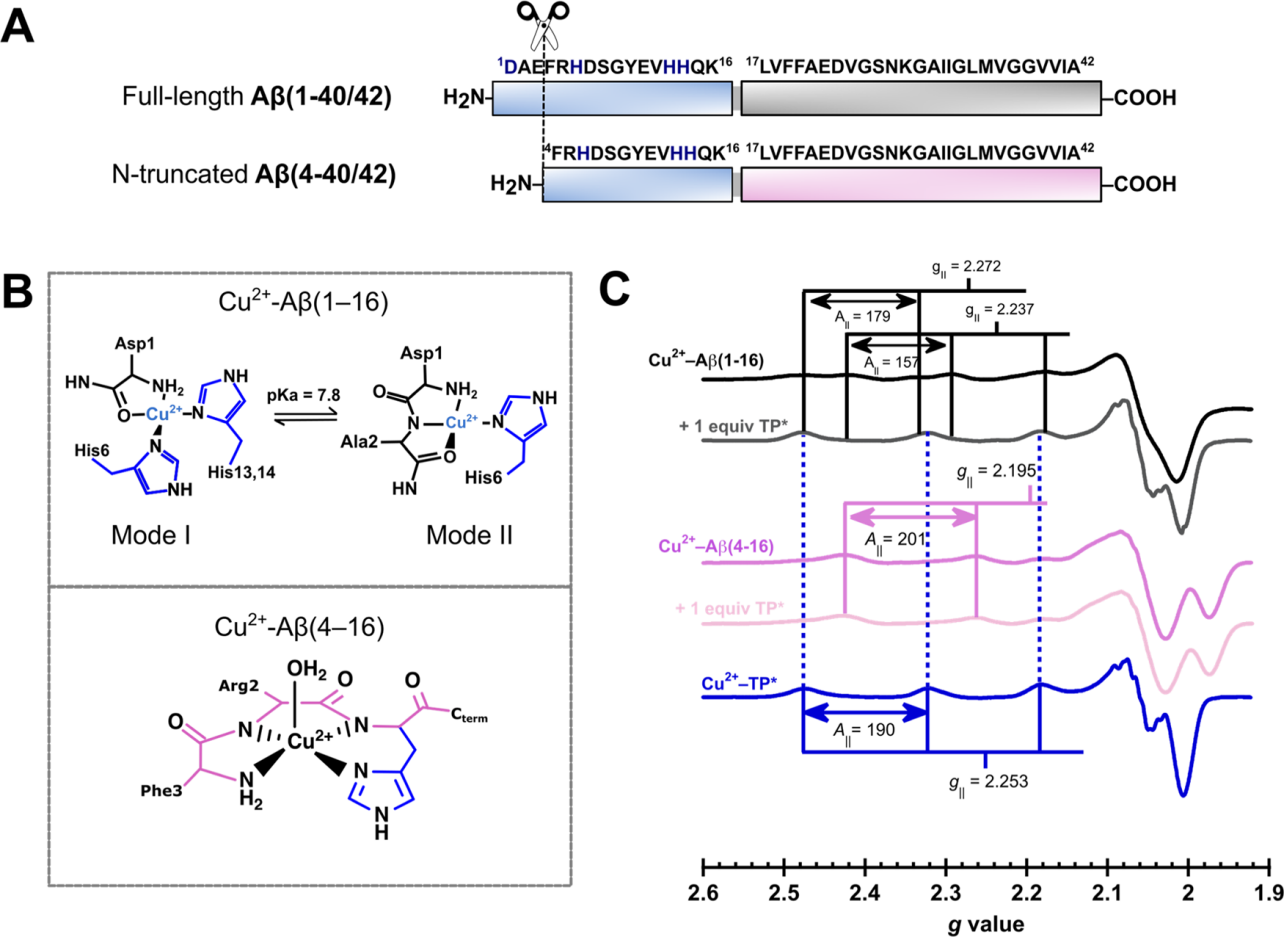
TP* can take Cu^2+^ away from Cu^2+^-Aβ(1–16) but not from Cu^2+^-Aβ(4–16). Full-length Aβ(1–16) is a 40–42 fragment peptide (A, top) that can be proteolytically cleaved to the *N*-truncated Aβ(4–16) variant. Cu^2+^ coordination to Aβ(1–16) and Aβ(4–16) can be assessed using the first 16 residues of their peptide sequence (A, blue box). Cu^2+^-Aβ(1–16) and Cu^2+^-Aβ(4–16) complexes bind Cu^2+^ very differently, as illustrated in (B). EPR spectra (C) of Cu^2+^- Aβ(1–16) (black spectrum) and Cu^2+^-Aβ(4–16) (dark pink spectrum). As previously reported, TP removes the metal from Cu^2+^- Aβ(1–16) (gray spectrum).^16^ On the other hand, after addition of 1 equiv of TP* to Cu^2+^-Aβ(4–16), the EPR spectrum remains unchanged (light pink spectrum). *A*_∥_ values are given in 1 × 10^–4^ cm^–1^.

Although Aβ(1–X) species are the most studied, *N*-truncated Aβ peptides, such as Aβ(4–X) species (Figure 4A), are highly abundant Aβ isoforms detected in both healthy and AD brains.^58−60^ Although both peptides bind Cu^2+^, their coordination properties and reactivities are completely different. Cu^2+^-Aβ(1–16) displays two coordination modes at physiological pH (Figure 4B, top panel): while Mode I involves the participation of the free NH_2_ group, the carbonyl backbone of Asp1, His6, and His13/14, Mode II includes the free NH_2_ moiety, a deprotonated amide from Ala2, and a His from His6/13/14.^61,62^ On the other hand, Cu^2+^ coordination of Cu^2+^-Aβ(4–16) is governed by its ATCUN binding motif (Figure 4B, bottom panel),^63,64^ in a similar fashion as observed for Cu^2+^-hCtr1 and Cu^2+^-HSA complexes. Moreover, while Cu^2+^-Aβ(1–16) complexes can produce reactive oxygen species, Cu^2+^-Aβ(4–16) cannot.^12,63−65^ Hence, Aβ(4–16) has been proposed to play a functional role in metal homeostasis.^63,64,66^ Therefore, an ideal Cu^2+^ chelator should remove Cu^2+^ from Aβ(1–40/42) but not from Aβ(4–40/42). TP removes the metal ion from the Cu^2+^-Aβ(1–16) species, as the addition of 1 equiv of TP to the Cu^2+^-Aβ(1–16) complex drastically changes the EPR spectrum, yielding signals identical to those of the Cu^2+^-TP complex (Figure 4C). In contrast, the EPR spectrum of the Cu^2+^-Aβ(4–16) complex remains unchanged upon addition of 1 equiv of TP*, displaying signals with *g*_∥_ = 2.195 and *A*_∥_ = 201 × 10^–4^ cm^–1^ (Figure 4C, pink lines), associated with Cu^2+^-Aβ(4–16) species. These results demonstrate that although TP can take Cu^2+^ away from Aβ(1–16) (Scheme 1), it does not remove the metal ion from Cu^2+^-Aβ(4–16) complexes.

### TP* Forms Ternary Complexes at the Cu^2+^-Binding Sites of the Prion Protein

**Figure 5:**
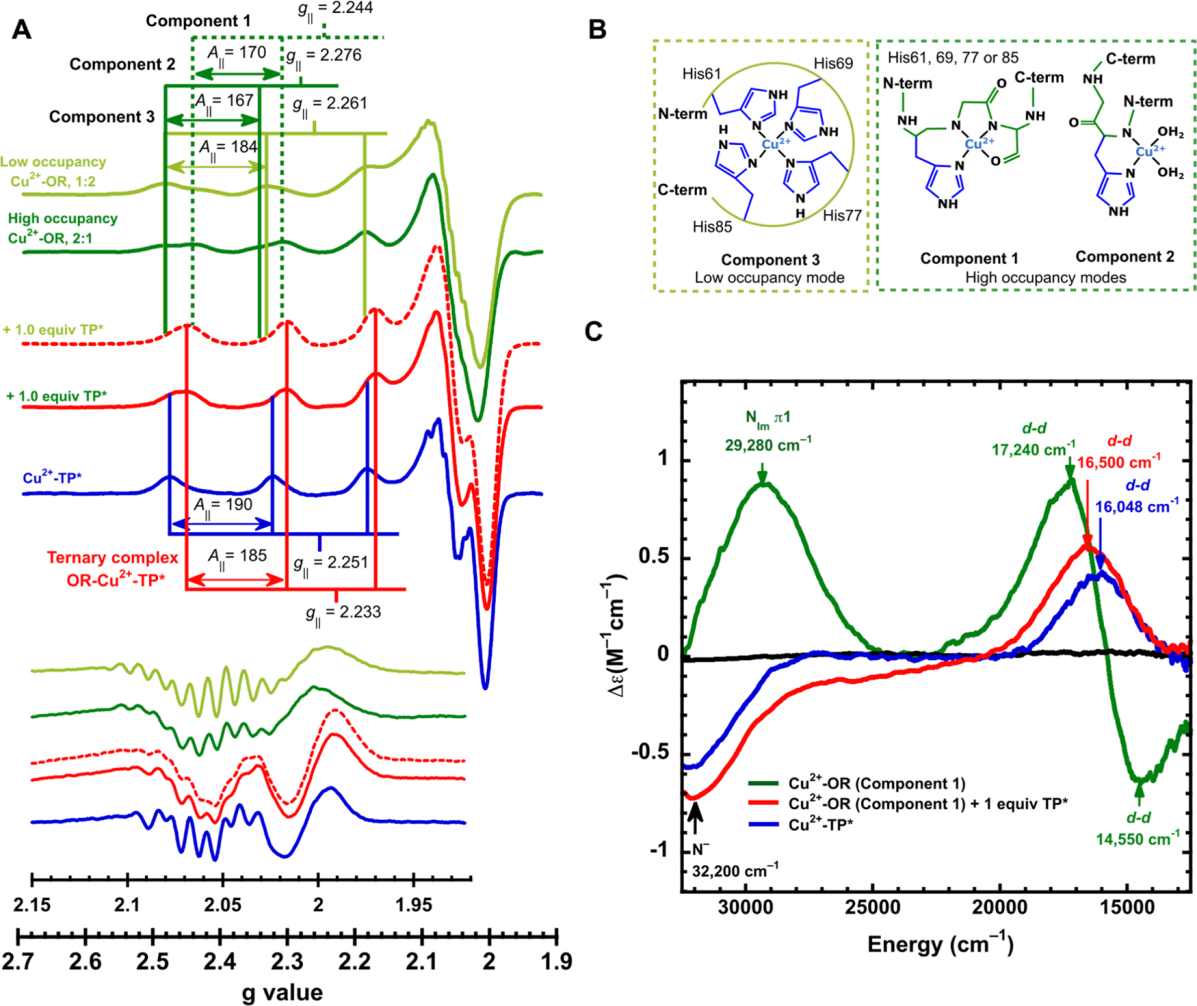
Competition for Cu^2+^ ions between TP* and the OR sites of PrP. EPR (A) spectra that correspond to the addition of 1 equiv of TP* (with respect to the metal ion) to the low- and high-occupancy modes of the Cu^2+^-PrP(60–91) (olive- and dark-green spectra, respectively) (the *A*_∥_ values are given in 1 × 10^–4^ cm^–1^). The corresponding Cu^2+^-coordination modes are shown in (B). Additions of TP* to both occupancy modes (red spectra) yielded a new set of signals associated with a ternary TP*-Cu^2+^-PrP(60–91) complex. (C) Addition of TP* to the high-occupancy modes (dark-green spectrum) was also followed by CD spectroscopy. Addition of 1.0 equiv with respect to the metal ion yielded signals similar but not identical bands (red spectrum) to that of the Cu^2+^-TP* complex (blue spectrum).

To study how TP* impacts Cu^2+^ binding to the OR sites of PrP^C^, the intrinsically disordered OR region was modeled by the PrP(60–91) peptide fragment. The Cu^2+^ binding in this region is governed by the metal concentration: At low Cu^2+^ concentration (up to 1:4 Cu^2+^/PrP^C^ ratio), one Cu^2+^ ion is bound to PrP^C^ using three or four His residues; this coordination mode is named component 3 or the low-occupancy mode (Figure 4B).^33,34^ At Cu^2+^ concentrations greater than 1:1 Cu^2+^/PrP^C^ ratio, each His residue binds one Cu^2+^ ion, yielding components 1 and 2 or high-occupancy modes (Figure 5B). The low-occupancy mode (component 3) was prepared as previously reported,^67^ using 1:4 ratio of Cu^2+^:PrP(60–91) at physiological pH = 7.4. The EPR spectrum of this complex exhibits a set of signals with *g*_∥_ = 2.261 and *A*_∥_ = 184 × 10^–4^ cm^–1^, consistent with previous reports (Figure 5A, olive-green spectrum).^34,67^ Upon addition of 1 equiv of TP* (with respect to the metal ion), a new set of signals with *g*_∥_ = 2.233 and *A*_∥_ = 185 × 10^–4^ cm^–1^ emerged (Figure 5A, dotted red spectrum) that do not correspond to the Cu^2+^-TP* complex. On the other hand, the high-occupancy modes were prepared using 2:1 ratio of Cu^2+^:PrP(60–91) at pH 7.4. The EPR spectrum displays two set of signals: one with *g*_∥_ = 2.244 and *A*_∥_ = 170 × 10^–4^ cm^–1^ associated with the component 1 and another set with *g*_∥_ = 2.276 and *A*_∥_ = 167 × 10^–4^ cm^–1^ corresponding to component 2 (Figure 5A, dark-green spectrum), as previously described.^34^ Upon addition of 1 equiv of TP* (with respect to the metal ion), a new set of signals with *g*_∥_ = 2.233 and *A*_∥_ = 185 × 10^–4^ cm^–1^ emerged (Figure 5A, continuous red spectrum), in a similar fashion to those observed for the case of the low-occupancy mode. In both cases, the spectroscopic features are distinct from those of the original Cu^2+^-PrP(60–91) complexes and from those of the Cu^2+^-TP* complex (Figure 4A). Overall, these results suggest the formation of a new ternary species composed of TP*, Cu^2+^, and PrP(60–91). Considering that components 3 is CD-silent,^67^ the titration with TP* was followed by CD spectroscopy only for the case of the high-occupancy modes. Component 1 displays two ligand field bands: a negative one at 14,550 cm^–1^ and a positive one at 17,240 cm^–1^ and a LMCT feature of high intensity at 29,280 cm^–1^ that is associated with the imidazole-to-copper signal (N_im_π_1_), while component 2 is also CD-silent (Figure 5C).^34^ Upon titration with TP*, the negative *d–d* transition and the LMCT bands disappear, while the positive one decreases in intensity and shifts to lower energy (16,500 cm^–1^), and a negative LMCT band at ∼32,200 cm^–1^ becomes evident. The CD spectrum at the final points of the titration (1 equiv of TP* with respect to the metal ion) is similar, but not identical, to that of the Cu^2+^-TP* complex (Figure 5C). Still, the *g* and *A* values for the species formed in the presence of TP, Cu^2+^, and PrP(60–91) are distinct from those of the original Cu^2+^-PrP complexes and the Cu^2+^-TP complex (Figure 5A). Moreover, the second derivative of the perpendicular region of the EPR spectra shows distinct nitrogen superhyperfine splitting patterns that do not correspond to those of the Cu^2+^-TP or the Cu^2+^-PrP complexes (Figure 5A, inset). Altogether, these results point to the formation of a ternary TP*-Cu^2+^-PrP(60–91) complex in the OR region that can be formed independently of the nature of the starting Cu^2+^-PrP(60–91) species (components 1, 2, and 3). Similarly, the impact of TP* on Cu^2+^-binding to the non-OR region of PrP was also evaluated by EPR and CD, finding that upon addition of TP*, a mixture of Cu^2+^-TP* and ternary TP*-Cu^2+^-PrP species is observed. These ternary complexes are similar in nature to those observed for the OR sites (SI text and Figure S6).

### Coordination Sphere of TP*-Cu^2+^-PrP Ternary Complexes

**Figure 6:**
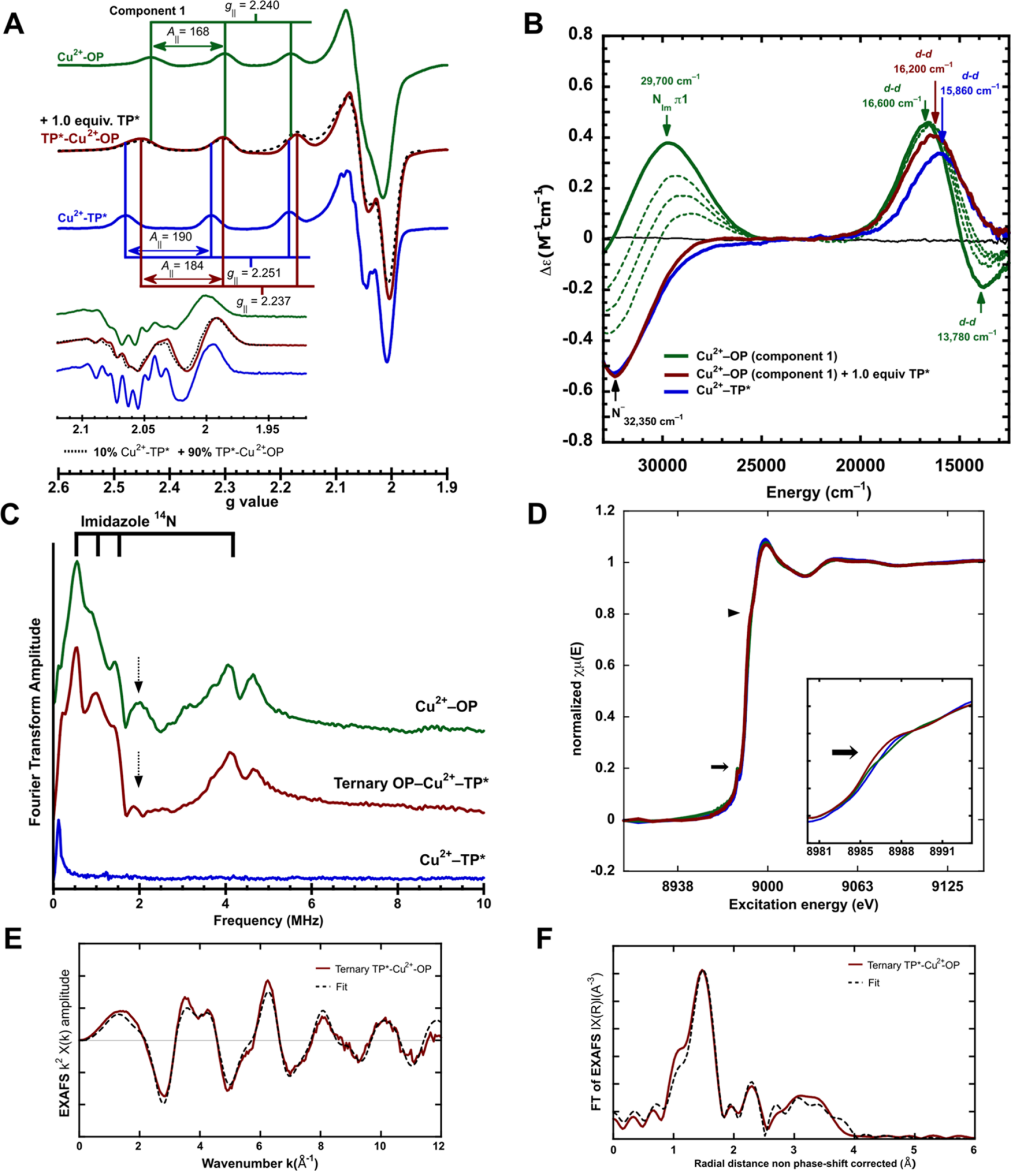
Spectroscopic analysis of the ternary TP*-Cu^2+^-OP complex. EPR (A), CD (B), ESEEM (C), and XANES (D) spectra of the Cu^2+^-OP (green), TP*-Cu^2+^-OP (maroon), and Cu^2+^-TP* (blue) complexes. EPR (A) shows the formation of the ternary TP*-Cu^2+^-OP complex after the addition of 1 equiv of TP* to the Cu^2+^-OP complex, and the second derivative of the perpendicular region is shown in the inset. CD-monitored titration (B) of the Cu^2+^-OP complex with increasing amounts of TP* (dotted green lines) yields the formation of the TP*-Cu^2+^-OP species. Three-pulse ESEEM spectra (C) of Cu^2+^-OP, TP*-Cu^2+^-OP, and Cu^2+^-TP* complexes: Cu^2+^-OP and ternary TP*-Cu^2+^-OP complexes display characteristic features assigned to the participation of a His ligand in their coordination environment. Black arrows indicate participation of backbone coordination to Cu^2+^ in the Cu^2+^-OP complex but not in the ternary complex. Normalized copper *K*-edge XANES spectra (D) of the three Cu^2+^-complexes. The black arrow represents the pre-edge features of XANES, and the black triangle points to the edge of the spectra. EXAFS and FT of EXAFS (in k^2^χ(k)) of the ternary TP*-Cu^2+^-OP complex are shown in (E) and (F), respectively, and the fit of experimental spectra is indicated as dotted dashed lines, using simulation parameters given in Table S10.

Given that TP* can form ternary complexes with all Cu^2+^-binding sites of PrP, as modeled by OR and non-OR fragments, it is crucial to demonstrate whether a single octapeptide (OP) model (Pro-His-Gly-Gly-Gly-Trp-Gly-Gln) that has a single Cu^2+^-binding site is able to form a ternary complex as the major species. As previously reported,^68^ the EPR spectrum of a Cu^2+^-OP complex prepared at pH = 7.4 using 1:1 ratio of Cu^2+^:OP exhibits only signals associated with component 1 with *g*_∥_ = 2.240 and *A*_∥_ = 168 × 10^–4^ cm^–1^ (Figure 6A, green spectrum), while its CD spectrum displays two ligand field bands (− 13,780 cm^–1^ and +16,600 cm^–1^) and a N_im_π_1_-to-copper LMCT at +29,700 cm^–1^ (Figure 6B). Upon addition of 1 equiv of TP*, a set of signals with *g*_∥_ = 2.237 and *A*_∥_ = 184 × 10^–4^ cm^–1^ emerged (Figure 6A, maroon spectrum), while by CD, the negative *d–d* transition and the positive LMCT band disappear, the positive *d–d* band shifts to ∼16,200 cm^–1^, and a negative LMCT appears at 32,350 cm^–1^ (Figure 6B, maroon spectrum). Remarkably, these spectroscopic features are very similar to those observed in ternary species formed with other Cu^2+^ binding sites of PrP (Figure S7, Tables S5 and S6). After the addition of 1 equiv of TP*, the EPR spectrum of the ternary TP*-Cu^2+^-OP complex shows a broadening, particularly evident in the first signal of the parallel region. The EPR spectrum was deconvoluted as a mixture of 10% Cu^2+^-TP* and 90% ternary TP*-Cu^2+^-OP (Figure 6A, dotted black lines), assuming that it has similar EPR features as those of the ternary TP*-Cu^2+^-PrP(60–91). Overall, these results demonstrate that a single octapeptide can also form a ternary TP*-Cu^2+^-PrP complex as the major species, and it is suitable for further characterization. The four ternary complexes identified so far, namely TP*-Cu^2+^-PrP(60–91), TP*-Cu^2+^-PrP(92–99), TP*-Cu^2+^-PrP(106–115), and TP*-Cu^2+^-OP, show similar spectroscopic features (Figure S7, Tables S5 and S6), mainly, a positive ligand field transition at ∼16,600 cm^–1^ and a negative LMCT at ∼32,400 cm^–1^, associated with an N^–^_amide_ to copper CT. The similarity in their CD spectra to those of the Cu^2+^-TP* complex suggests a comparable geometry but not necessarily an identical set of ligands. The EPR spectrum of this ternary species was simulated using *g*_∥_ = 2.237 and *A*_∥_ = 184 × 10^–4^ cm^–1^ (Figure S8, Table S7) that, according to Peisach–Blumberg correlations,^69^ correspond to an equatorial coordination sphere with more nitrogen character than that of the Cu^2+^-TP* complex (Figure S9). Hence, the ternary complex might involve one more nitrogen-based ligand, likely His, that could be provided by PrP.

**Scheme 3:**
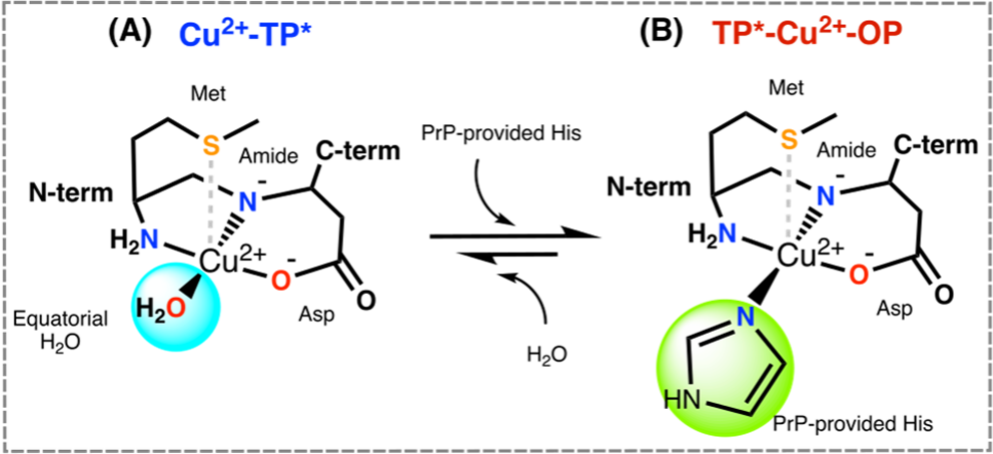
Coordination modes of (A) Cu^2+^-TP and (B) ternary TP-Cu^2+^-OP complexes at pH = 7.4, with possible 3N1O or 3N1O1S coordination modes

To test for the coordination of a His residue in the TP*-Cu^2+^-OP complex, we employed ESEEM (electron spin echo envelope modulation) spectroscopy. ESEEM is a pulsed EPR technique that detects His coordination through magnetic coupling of the imidazole remote ^14^N to the Cu^2+^ ion.^70^ ESEEM spectra were collected at pH = 7.4 for the Cu^2+^-TP*, Cu^2+^-OP, and TP*-Cu^2+^-OP complexes (Figure 6C); the latter two display the characteristic features assigned to the remote nitrogen of a copper-bound imidazole. Specifically, the low-frequency interactions at ∼0.50, 1.00, and 1.41 MHz (Figure 6C) are typical of a weakly coupled ^14^N nucleus to copper.^68,70^ The ESEEM spectra also show a characteristic broad signal double quantum (DQ) feature at ∼4.11 MHz. The DQ feature along with the peak observed at ∼2 MHz (dotted black arrow) has been assigned to the OP backbone coordination to Cu^2+^.^68^ Both features are decreased in the ternary TP*-Cu^2+^-OP complex (Figure S10). None of these transitions are observed for the Cu^2+^-TP* complex (Figure 6C, blue spectrum), consistent with the fact that it does not contain His ligands in its coordination sphere (Figure 2B, inset). These findings demonstrate that a His from PrP participates in the coordination sphere of the ternary TP*-Cu^2+^-PrP complex. The main difference between Cu^2+^-TP* and the ternary TP*-Cu^2+^-PrP complex is the replacement of an oxygen ligand by a PrP-provided His residue (Scheme 3). To identify the nature of the exchangeable ligand that is replaced by the His residue, we prepared Cu^2+^-TP* and TP*-Cu^2+^-OP complexes in 60–70% ^17^O-enriched water. (Figure S11, Tables S8 and S9). Although both EPR spectra are affected by ^17^O enrichment, only the spectrum of Cu^2+^-TP* displays a significant broadening (∼14 G), as previously reported,^71^ suggesting that a PrP-provided His replaces the water molecule from the Cu^2+^-TP* coordination sphere, as shown in Scheme 3.

To gain insights into the local coordination environment of the ternary complex, comparative copper K-edge X-ray absorption near-edge structure (XANES) (Figure 6D) and extended X-ray absorption fine structure (EXAFS) spectroscopy of the ternary complex were performed (Figures 6E,F and S11). The XANES region provides information on the geometrical arrangement of the ligands around the metal center.^72,73^ The three Cu^2+^ complexes display a weak pre-edge shoulder at 8987–8988 eV (Figure 6D, inset black arrow) that is often associated with square pyramidal complexes with axial ligands or distant solvent atoms.^72,73^ Moreover, the edge 1*s* → 4*p* transitions for the Cu^2+^-OP, TP*-Cu^2+^-OP, and Cu^2+^-TP* complexes appear in the range of 8998.3 to 8999.0 eV (Figure 6D, black triangle), and considering the energy of the copper metal K-edge as 8991.0 eV, the corresponding edge shift range is 7.3 to 8 eV, which are values commonly observed for Cu^2+^ square pyramidal species. Furthermore, the normalized intensity of the edge is in the range of 1.07–1.08, falling between the values commonly observed for Cu^2+^ square pyramidal species.^72^

Finally, the EXAFS region of the spectra provides information about the local structure of the complex, the nature of the neighboring atoms, and their metal–ligand distances.^72^ Fitting of the EXAFS spectrum and its FT for the TP*-Cu^2+^-OP complex was performed considering a 3N1O1S coordination shell containing three Cu^2+^-N (∼2.00 Å), one Cu^2+^-O (2.20 Å), and one Cu^2+^-S (2.67 Å) interactions (Figure 6E,F, Table S10). This coordination sphere yields a significantly better fit as compared to that of a 3N1O shell (Figure S12 and S13, Table S11), suggesting an actual contribution from the Cu^2+^-S interaction. This is in good agreement with a 3N1O1S coordination mode arranged in square pyramidal geometry (Scheme 3).

### TP* Forms a Ternary Complex with the Full-Length Prion Protein

**Figure 7:**
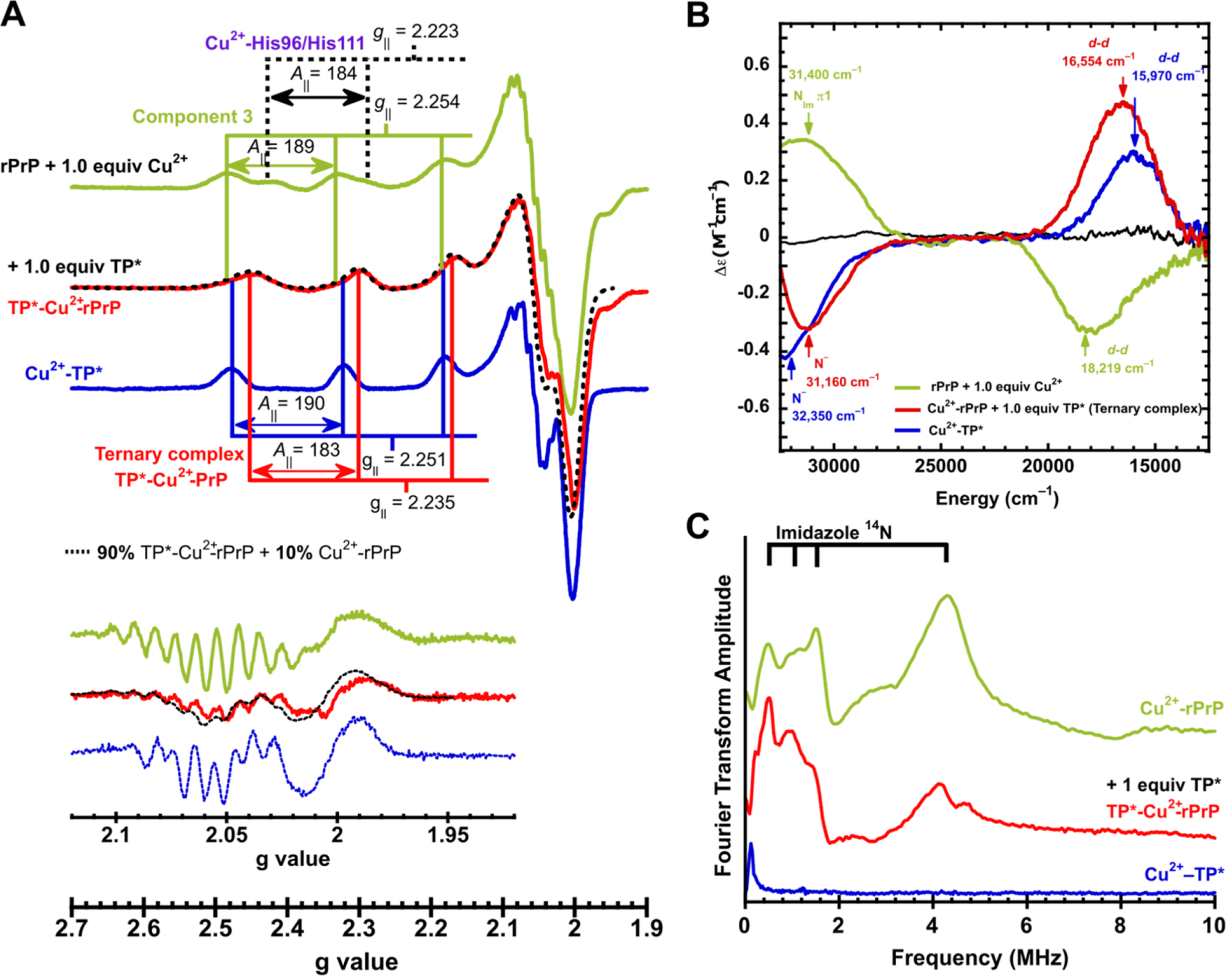
Competition for Cu^2+^ between TP and the rPrP. EPR (A), CD (B), and ESEEM spectra (C) of the ternary TP*-Cu^2+^-rPrP complex. (A) Cu^2+^-rPrP complexes (green lines) were prepared using a Cu^2+^-rPrP ratio of 1:1 and mixtures of component 3 and Cu^2+^-His96/His111 are evident. Addition of 1 equiv of TP* results in the formation of ternary TP*-Cu^2+^-rPrP species (red lines). The spectra were deconvoluted as a mixture of species represented in black dotted lines. The second derivative of the perpendicular region is shown in the inset. (B) CD spectra are consistent with the formation of ternary TP*-Cu^2+^-rPrP species. (C) Three-pulse ESEEM spectra of Cu^2+^-rPrP, TP*-Cu^2+^-rPrP, and Cu^2+^-TP* complexes: Cu^2+^-rPrP and ternary TP*-Cu^2+^-rPrP complexes display characteristic features assigned to the participation of an imidazole (His) ligand in their coordination sphere. *A*_∥_ values are given in 1 × 10^–4^ cm^–1^.

The formation of a ternary TP*-Cu^2+^-PrP complex was also evaluated by using the full-length recombinant murine prion protein (rPrP) with 1 equiv of Cu^2+^ at pH = 7.4. At such a metal:rPrP ratio, component 3 is the main species at the OR region and a mixture of Cu^2+^ bound to His96 and His111 at the non-OR region.^38,74^ Consistently, two sets of EPR signals can be discerned: one with *g*_∥_ = 2.254 and *A*_∥_ = 189 × 10^–4^ cm^–1^ corresponding to component 3 and another one with *g*_∥_ = 2.223 and *A*_∥_ = 184 × 10^–4^ cm^–1^ associated with Cu^2+^ bound to His96 and His111 (Figure 7A). Although component 3 is CD-silent,^33^ the CD spectrum of rPrP with Cu^2+^ displays the characteristic LMCT and *d–d* bands associated with Cu^2+^ bound to the non-OR sites (Figure 7B).^38^ Addition of 1 equiv of TP* yielded CD (Figure 7B) and EPR (Figure 7A) signals that do not correspond to those of Cu^2+^-TP* or the Cu^2+^-rPrP complexes. Hence, a ternary TP*-Cu^2+^-rPrP species is formed. The EPR spectrum showed signals with *g*_∥_ = 2.235 and *A*_∥_ = 183 × 10^–4^ cm^–1^ and a distinct nitrogen superhyperfine pattern, that can be deconvoluted as a mixture of 10% Cu^2+^-rPrP and 90% ternary TP*-Cu^2+^-rPrP (Figure 7A, black dotted lines), assuming that it has similar EPR features as those of the ternary Cu^2+^-TP*-PrP(60–91) complex. The participation of rPrP His residues in the ternary TP*-Cu^2+^-rPrP species was probed by ESEEM (Figure 7C). The ternary complex (red) and Cu^2+^-rPrP (green) display NQI at ∼0.48, 1.11, and 1.49 MHz and a DQ line at ∼4.23 MHz, assigned to the remote nitrogen of a Cu^2+^-bound imidazole. Additionally, the spectrum for the ternary TP*-Cu^2+^-rPrP displays a decreased intensity in the DQ/NQI ratio, as compared with the multi-His coordination mode of the Cu^2+^-rPrP complex. (Figure S14) This difference is associated with a decreased His coordination mode for the ternary TP*-Cu^2+^-rPrP complex. Moreover, the ESEEM spectrum of TP*-Cu^2+^-rPrP is practically identical to that of the TP*-Cu^2+^-OP ternary complex (Figure S15). Altogether, these results demonstrate that the TP* forms ternary complexes with the Cu^2+^-binding sites of the rPrP with a similar coordination sphere to that of the extensively characterized TP*-Cu^2+^-OP complex.

### TP* Does Not Impact the Cu^2+^-Promoted *cis*-Interdomain Interaction of the rPrP

**Figure 8:**
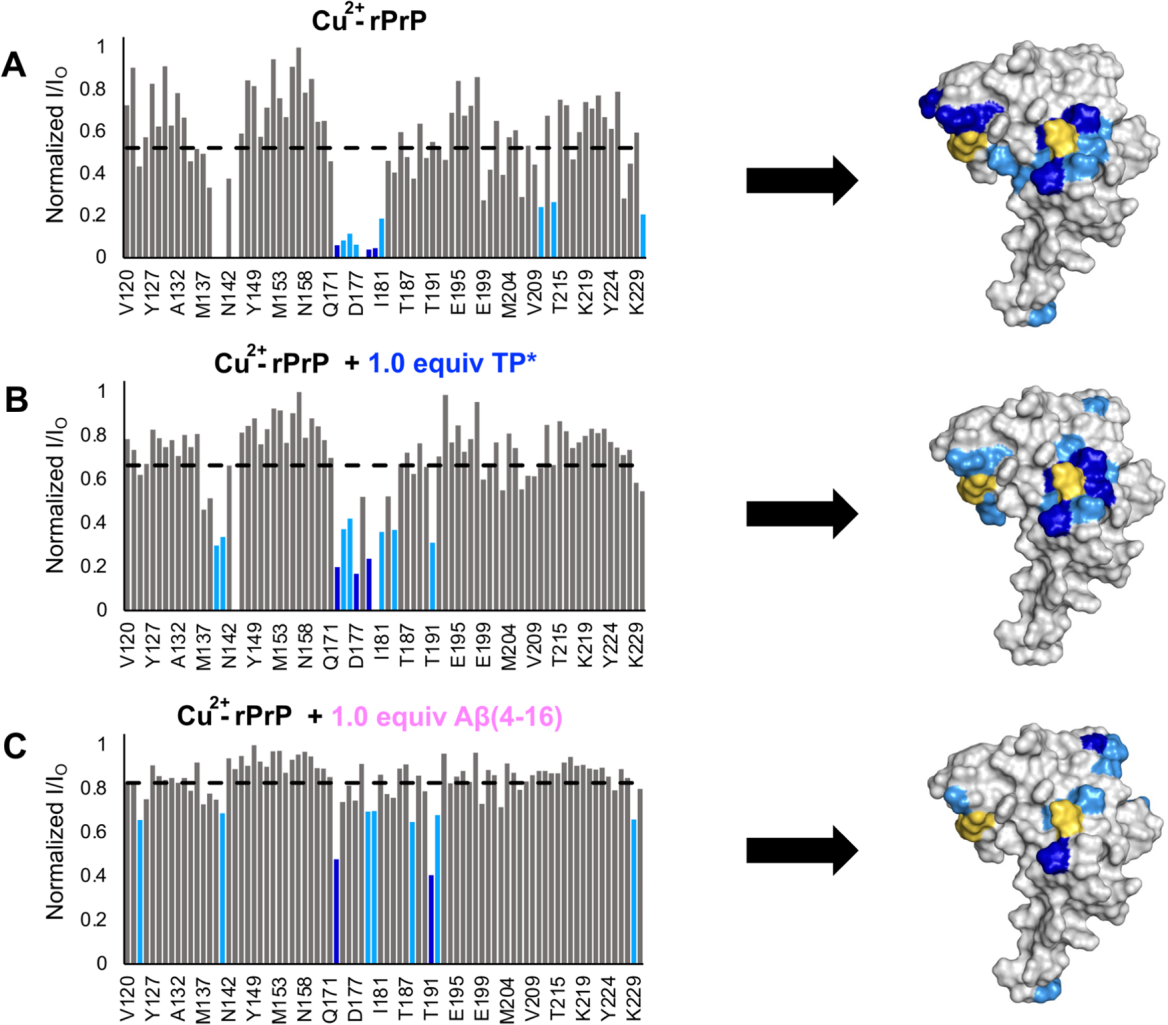
Observed PREs of the *cis*-interdomain interaction in Cu^2+-^rPrP complexes. Bar plots showing the magnitude of the ^1^H–^15^N HSQC NMR peak intensity reduction derived from the PREs on specific residues in the rPrP. Normalized I/I_O_ values for rPrP-Cu^2+^ (A), rPrP-Cu^2+^ + 1.0 equiv TP* (B), and rPrP-Cu^2+^ + Aβ(4–16) are plotted against rPrP residues. Gray bars represent peaks unaffected by PREs, light blue represents relatively weak PREs, and dark blue represents relatively strong PREs. These PREs are mapped onto the surface of PrP (PDB: 1XYX), where His139 and His176 are in yellow.

Given that TP* forms ternary TP*-Cu^2+^-rPrP complexes, it is essential to understand how this would affect the neuroprotective the *cis*-interaction of rPrP that is stabilized by Cu^2+^.^41^ Therefore, to ensure that the *cis*-interdomain interaction occurs still in the presence of TP*, we employed ^1^H–^15^N heteronuclear single quantum coherence (HSQC) spectroscopy. This NMR technique can monitor the interaction between Cu^2+^ and the *C*-terminus. The paramagnetic property of Cu^2+^ causes distance-dependent paramagnetic relaxation effects (PREs), which decrease the intensity of *C*-terminal residue peaks in the HSQC. By dividing the peak intensities of the Cu^2+^-containing sample (I) by those of WT rPrP with no metal (*I*_O_), *I*/*I*_O_ is calculated to determine which residues are near the Cu^2+^ center (Figure 8A). Additionally, these values were mapped onto the surface of the three-dimensional structure of the rPrP *C*-terminal domain (PDB: 1XYX). The *cis*-interdomain interaction (Figure 8A) is characterized by having two patches (blue shade of colors) of significantly decreased intensity due to PREs around His139 and His176 on the surface of the *C*-terminus (yellow marks). Upon addition of 1 equiv of TP* to the Cu^2+^-rPrP complexes, the same residues experience PREs (Figure 8B), confirming that the *cis*-interdomain interaction persists and that TP* does not disturb it. Moreover, when TP* is added to rPrP in the absence of Cu^2+^, the HSQC spectrum shows insignificant changes (Figure S16), suggesting that they do not interact in the absence of Cu^2+^. As demonstrated above, TP* transfers Cu^2+^ to ATCUN sites from hCtr1(1–14), HSA, and Aβ(4–16). The latter was chosen as a proof of concept to evaluate if high-Cu^2+^ affinity chelating agents can perturb the effects of the PREs in Cu^2+^-rPrP species. Indeed, the addition of 1 equiv of Aβ(4–16) to the Cu^2+^-rPrP complexes dramatically decreases the PREs around His139 and His176 residues, (Figure 8C) confirming the strong chelating abilities of Aβ(4–16). Altogether, these results indicate that TP* does not significantly perturb the neuroprotective Cu^2+^-induced *cis-*interdomain interaction of the rPrP, while higher affinity Cu^2+^-chelating molecules can impair this conformation.

### Effect of TP* in the Colocalization of PrP^C^ and NMDARs

**Figure 9:**
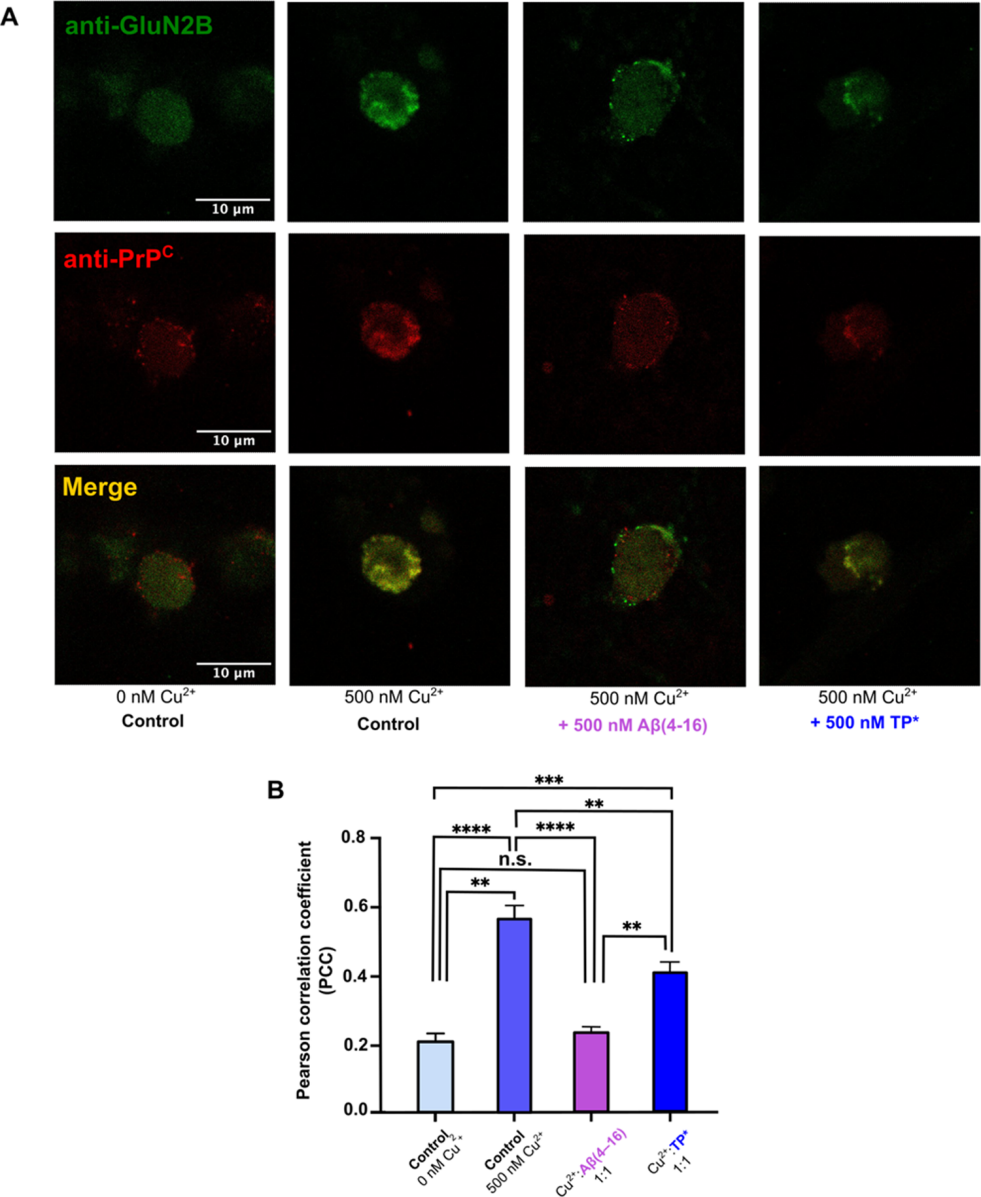
Effect of TP* in the Cu^2+^-dependent colocalization of PrP^C^ and NMDAR in SK-N-SH cells. Colocalization analysis was performed by immunocytochemistry using fluorescently labeled secondary antibodies: green for the GluN2B and red for PrP^C^. Representative images of colocalization in conditions without copper (control); 500 nM Cu^2+^ (control); 500 nM Cu^2+^/Aβ(4–16); and 500 nM Cu^2+^/TP* in a ratio of 1:1 of Cu^2+^:peptide. The Pearson correlation coefficient (PCC) was employed to quantify the colocalization between both fluorescent probes. Comparison of the PCC values of the different experimental conditions (B). All comparisons show statistical difference, except comparison between no copper control and Cu^2+^:Aβ(4–16) condition (data are the means ± SEM, one-way ANOVA, post hoc Tukey; *P < 0.05, ***P < 0.001, ****P < 0.0001).

We next evaluated the impact of TP* in the Cu^2+^-dependent colocalization of PrP^C^ and NMDARs. The PrP^C^–NMDAR interaction has been previously demonstrated by functional studies using a metal concentration of ∼100–500 nM.^25,26^ In this study, confocal immunofluorescence was used to detect fluorescently labeled secondary antibodies for the GluN2B subunit of NMDARs and PrP^C^. Colocalization allows us to determine if both probes codistribute in the cell membrane with one another.^75^ To evaluate whether TP* or the stronger chelating agent Aβ(4–16) can disrupt this colocalization, we used the human SK-N-SH neuroblastoma cell line differentiated with retinoic acid (RA). Under these conditions, cells adopt a neuronal phenotype, expressing both proteins in the cell membrane (Figures S17 and S18). Colocalization of PrP^C^ and NMDAR was tested in a wide range of metal concentrations (50 nM, 500 nM, 5 μM, and 50 μM). As observed in Figure S19, colocalization depends on Cu^2+^ concentrations, reaching a maximum at 500 nM Cu^2+^, as indicated by the Pearson correlation coefficient (PCC) (Figure S19).^75^ At such metal concentrations, the impact of TP* and Aβ(4–16) in the colocalization of PrP^C^ and GluN2B was evaluated (Figures 9A and S20). Addition of 500 nM Aβ(4–16) or TP* significantly decreased PCC values as compared to those of positive Cu^2+^ control conditions (Figure 9B and S21). However, while Aβ(4–16) completely abolished colocalization between both proteins, reaching PCC values comparable to controls with no copper, the effect of TP* is less pronounced, with PCC values significantly different from those in the absence of metal ions (Figures 9B and S21). It is important to note that these effects are not due to changes in protein expression upon the different treatments (Figure S22). Overall, these results indicate that high-affinity Cu^2+^ chelators can disturb the colocalization of PrP^C^ and NMDARs, while TP* only displays a mild effect, and hence, it would have a minor impact in this Cu^2+^-dependent neuroprotective mechanism.

## Discussion

**Scheme 4:**
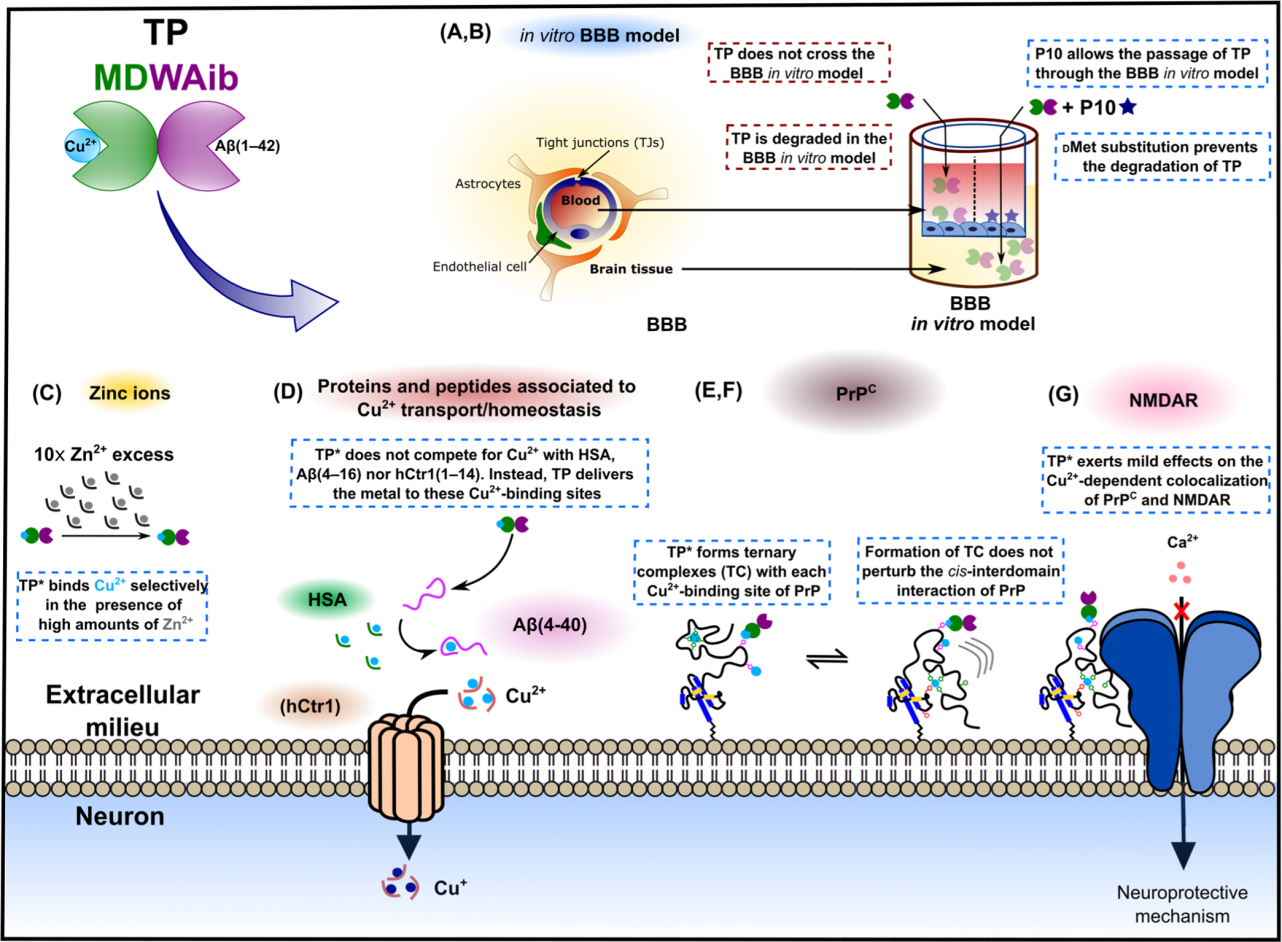
Schematic Representation of the Principal Results of This Work In Summary, Our Findings Indicate That (A, B) TP Does Not Cross the BBB *In Vitro* Model and Is Degraded. However, Its Coadministration with P10 Allow Its Passage Using the Paracellular Pathway and the Substitution of L-to-D Met in the Sequence of TP Prevents Its Degradation. (C) Metal Binding of Optimized TP* is Highly Selective for Cu^2+^ in the Presence of High Amounts of Zn^2+^. (D) TP* Does Not Compete for Cu^2+^ with Proteins/Peptides Associated to Copper Trafficking, Such as hCtr1, HSA, and Aβ(4–40); Instead, TP* Can Transfer the Metal Ion to These Species. (E) TP* Forms Ternary TP*-Cu^2+^-PrP Complexes with Peptide Models of Each of the Cu^2+^-Binding Sites of PrP and with the Full-Length rPrP; however, (F) Formation of Ternary Species Does Not Perturb Importantly the cis-Interdomain Interaction of the Protein. Finally, (G) TP* Exerts Only Mild Effects in the Cu^2+^-Dependent Colocalization of PrP^C^ and NMDARs.

In this study, we used a multidisciplinary strategy to evaluate the desirable features of a peptide, TP, with therapeutic potential for AD, including its bioavailability in a BBB model, metal-binding preference, and its impact on Cu^2+^ to proteins involved in metal homeostasis and neuroprotection, such as PrP and its Cu^2+^-dependent colocalization with NMDARs. Scheme 4 summarizes our findings. Although TP does not cross the BBB, coadministration with the P10 peptide facilitates its passage through the paracellular pathway. TP is degraded in the BBB model; however, this process was decreased by changing the stereochemistry of the amino acid in the first position (d-Met). The optimized sequence (TP*) preserves its bifunctional properties, it displays the same Cu^2+^-binding properties as TP, and it competes for Cu^2+^ ions with Aβ(1–40), preventing the peptide from taking the metal-induced aggregation pathway (Figure S3). TP* also binds Cu^2+^ ions selectively, even in the presence of high amounts of Zn^2+^, and it does not compete for Cu^2+^ ions with ATCUN sites in proteins that play key roles in copper trafficking and homeostasis, such as hCtr1 and HSA, or the *N*-truncated Aβ(4–16). Interestingly, TP* forms ternary complexes with every Cu^2+^-binding site of PrP, but the formation of these species does not perturb the physiologically relevant *cis*-conformation of rPrP. In contrast to the case of high-affinity Cu^2+^ chelators, such as the ATCUN-containing Aβ(4–16), TP* exerts only minor effects on the Cu^2+^-dependent colocalization of PrP^C^ and NMDAR (Scheme 4).

Peptide-based drugs display a low permeability through the BBB and are susceptible to proteolytic degradation.^44^ Indeed, TP cannot cross the BBB through the cells (transcellular pathway) using transporters, it is too “bulky” to cross between cells (paracellular pathway), and it is proteolytically degraded. In this work, TP passage through the BBB model was enhanced by using a strategy that targets the paracellular pathway. P10 is a peptide that opens the BBB, as it is derived from the sequence of rotavirus protein VP8 that reversibly opens TJs in complex environments such as mouse models.^47^ This approach modulates the size exclusion properties of the barrier interfering with the formation of TJs, allowing diffusion of molecules from the apical to the basal compartments in analogy to the passage from the bloodstream to the brain.^47^ Indeed, coadministration of TP with P10 allowed the detection of TP in the basal compartment of the BBB cell model (Scheme 4 A,B). Overall, this strategy can be employed for other bioactive molecules with therapeutic potential, such as peptides, proteins, or oligonucleotides, that cannot cross through the transcellular route, to enhance their delivery.^76^ In fact, in recent studies, it has been demonstrated that FDA-approved drugs and excipients open tight junctions, including those found in the BBB.^47^ On the other hand, the inability of TP to cross cellular membranes by itself may be advantageous, as once it is delivered in the brain, TP would be mainly distributed in the extracellular milieu where its therapeutic targets (Aβ and Cu^2+^) are present. Moreover, the degradation of TP was prevented by changing the stereochemistry of Met-1. This is consistent with the presence of aminopeptidases in the BBB that recognize only the first amino acid at the *N*-terminus of peptide sequences. Hence, the use of a d-amino acid in the first position is an important feature in the design of peptide-based drugs. Our permeability assays showed promising *in vitro* results; however, further characterization is required to validate and optimize the use of P10 and TP* as a therapeutic strategy.

A desirable aspect of a copper chelator for AD is the specific binding to its target metal ion, without interfering with the functions of other metal ions at the synapse,^77^ such as Zn^2+^. From a therapeutical point of view, selective Cu^2+^ binding of TP* might prevent interference with Zn^2+^-dependent functions, such as enzymatic activity, including metalloproteinases that process APP through the nonamyloidogenic pathway,^78,79^ Zn^2+^-signaling processes, and oxidative stress, among others^79^ (Scheme 4C). Our spectroscopic results demonstrated that TP* binds Cu^2+^ in conditions when both Cu^2+^ and Zn^2+^ ions are available. While this is, in fact, a remarkable feature of TP*, additional studies would be required to evaluate the impact of TP* in Zn^2+^ homeostasis and Zn^2+^-dependent functions at the synapse.

Copper is an essential cofactor of key metalloenzymes,^80^ and selective Cu^2+^ chelators designed for AD should not indiscriminately remove copper from proteins involved in copper trafficking and homeostasis,^17,23^ such as hCtr1 and HSA. A critical feature of TP* is that it does not compete for Cu^2+^ ions with the ATCUN sites of such proteins. These findings are in line with the affinity of ATCUN sites for Cu^2+^, with the dissociation constant (K_d_) in the range of 10^–12^ M,^81^ as compared to TP*, with an estimated K_d_ of ∼10^–10^ M for the *N*-term MD sequence shared with α-synuclein protein.^82^ Such a large difference in affinities is consistent with the fact that TP* can deliver Cu^2+^ ions to the ATCUN sites of hCtr1 and HSA, and it further suggests that, in a clinical setting, TP* would bind excess free copper to deliver it to copper-trafficking proteins, although this requires further evaluation to shed light on the impact of TP* in copper homeostasis.

On the other hand, *N*-truncated Aβ(4–42) is another ATCUN-containing species at the brain that arises from nonspecific proteolytic cleavage of APP, and it constitutes ∼50–60% of amyloid plaques in AD patients.^58−60^ Although its physiological role is still unknown, emerging evidence shows that Aβ(4–X) can take Cu^2+^ from Aβ(1–X), forming redox-silent Cu^2+^-Aβ(4–X) species, that might provide redox-protective functions in the brain.^63,64,83^ Our results indicate that TP* does not remove the metal from the Cu^2+^-Aβ(4–X) species; hence, it would not disrupt the formation of redox-inert Cu^2+^-Aβ(4–X) complexes. This is important in the context of AD pathology, where redox imbalances contribute significantly to the progression of disease.^84^ Future studies should inform on the ability of TP* to protect neurons from Cu^2+^-catalyzed oxidative damage.

A paramount strategy in this study was to explore the effect of TP* on Cu^2+^ coordination to PrP, another AD-related copper-binding protein, beyond Aβ(1–X) species.^85^ PrP^C^ is one of the main copper-binding proteins at the synapse and it has been recently implicated with AD-neurotoxic mechanisms; it colocalizes in the amyloid plaques of AD patients^86−89^ and binds Aβ monomers and oligomers,^90,91^ triggering neurotoxic pathways and promoting Aβ uptake.^92^ This is indeed the first time that the impact of a copper chelator with therapeutic potential for AD in copper binding to PrP^C^ is evaluated. Our spectroscopic results show that TP* forms ternary complexes with each of the Cu^2+^-binding sites of PrP (Scheme 4D,E). Combining a wide range of spectroscopic tools, a molecular-level description of the TP*-Cu^2+^-PrP species was achieved, showing that it retains the coordination sphere of the Cu^2+^-TP* complex, except that the equatorial water molecule is replaced by a PrP-provided His ligand (Scheme 3). A similar behavior has been reported for PBT2, a phase-2 clinically tested therapeutic ionophore for AD.^93,94^ The Cu^2+^-PBT2 complex also includes a “labile” equatorial Cl^–^ ligand that can be replaced by a His provided by Aβ(1–X), histamine, or virtually any donor of His side chains.^95−97^ In this sense, PBT2 shows a “promiscuous” formation of ternary complexes, while TP* selectively forms ternary species with PrP^C^, but not with ATCUN sites, or Aβ(1–16). This behavior of TP* suggests that the propensity to form ternary complexes depends not only on Cu^2+^ binding affinity but also on additional factors, such as hydrophobic, steric, and electrostatic effects that could stabilize ternary interactions. While the therapeutic benefits of copper chelators that form ternary complexes are still under debate,^98,99^ our NMR results revealed that TP* does not impact the *cis*-interdomain interaction of PrP^C^, while the high-affinity copper chelator Aβ(4–16) nearly abolished this “folded” conformation of rPrP. This points to potentially toxic side effects when using high-affinity copper chelators to prevent Cu^2+^-Aβ(1–X) interactions. Moreover, these findings are consistent with the observed impact of TP* and Aβ(4–16) in the Cu^2+^-dependent colocalization of PrP^C^ and NMDAR: while TP* displays only a minor effect, *N*-truncated Aβ(4–16) abolishes this colocalization (Scheme 3G). Overall, these findings strongly suggest that the moderate affinity and unique coordination properties of TP* could lessen undesired effects on Cu^2+^-dependent physiological mechanisms, while the use of high-affinity copper chelators might disturb a plethora of Cu^2+^-dependent neuroprotective mechanisms. Although additional cellular studies are necessary to evaluate the impact of TP* in the function of PrP^C^ and its interaction with other proteins involved in memory and learning processes, this study provides a molecular basis for understanding such interactions. Future research focused on characterizing the efficacy and safety of TP* through *in vitro* and *in vivo* models is necessary, with the hope of translating our findings into clinical therapies.

## Concluding Remarks

In this study, we have evaluated and improved the drug-like properties of a bifunctional peptide with therapeutic potential for AD. TP* harbors desirable features for a copper chelator against AD, including enhanced BBB permeability and degradation resistance; metal selectivity; and the ability to prevent Cu^2+^-Aβ(1–X) interactions, without impacting Cu^2+^ binding to proteins that are important for copper trafficking or neuroprotective mechanisms. More importantly, this is the first study that points to PrP^C^ as an important player in the characterization of copper chelators against Alzheimer’s disease.

The multidisciplinary approach used here to evaluate such properties of TP* underscores the importance of having continuous feedback between biophysical and biological studies in the design and optimization of metal-targeting drugs. This strategy might inspire the optimization and screening of other therapeutic metal chelators before being tested in animal models and clinical trials. Although further studies are necessary to uncover the therapeutic benefits of TP* in AD models, this work provides molecular outcomes that contribute to elucidating its action mechanisms.

## Supporting Information Available

Changes in the transendothelial electrical resistance after administration of TP, P10, and TP+P10, chromatographic profile of TP samples incubated in the BBB model and identification of degradation products by MS; bifunctional properties of TP and TP* variants; peptide models; EPR parameters of the final point of titrations of Cu^2+^-TP* with ATCUN sites; titration of Cu^2+^-TP* with HSA followed by CD, CD parameters for the final point of titrations of Cu^2+^-TP* with ATCUN species; EPR and CD spectra of ternary TP*-Cu^2+^-PrP species of reported ternary complexes; competitions for Cu^2+^ between TP* and non-OR sites of PrP; EPR and CD parameters of ternary complexes; EPR simulation and spin Hamiltonian parameters; Peisach–Blumberg correlations; ESEEM comparisons between Cu^2+^-OP and ternary OP-Cu^2+^-TP*spectra; comparative EPR spectra of Cu^2+^-TP* and TP*-Cu^2+^-OP complexes prepared in ^16^O water and ^17^O-enriched water; comparison of EXAFS and FT of EXAFS spectra of Cu^2+^-OP, Cu^2+^-TP*; and TP*-Cu^2+^-OP samples; EXAFS fit of the ternary TP*-Cu^2+^-OP complex using a 3N1O1S coordination sphere; EXAFS fit of the ternary TP*-Cu^2+^-OP complex using a 3N1O coordination sphere; ESEEM comparisons between Cu^2+^-rPrP and ternary OP-Cu^2+^-TP* spectra; ESEEM comparison between ternary TP*-Cu^2+^rPrP and ternary TP*-Cu^2+^-OP spectra; comparative XAS spectra of TP*-Cu^2+^-rPrP, Cu^2+^-rPrP; and Cu^2+^-TP* complexes; effect of TP* in the PREs of the *cis*-interdomain interaction in the absence of Cu^2+^; differentiation protocol for the SK-N-SH cell line using retinoic acid; expression of NMDAR and PrP^C^ in the RA-differentiated SK-N-SH cells; effect of Cu^2+^ in the colocalization of PrP^C^ and NMDARs; multiple comparisons of the effect of TP* in the Cu^2+^-dependent colocalization of PrP^C^ and NMDAR; and expression of PrP^C^ in the different experimental conditions (PDF)

## Abbreviations

AD: Alzheimer′s disease
Aβ: Amyloid β
TP: Tetrapeptide
TP*: optimized sequence of TP
BBB: blood–brain barrier
PrP^C^: cellular prion protein
NMDAR: *N*-methyl-d-aspartate receptor
EPR: electron paramagnetic resonance
NMR: nuclear magnetic resonance
APP: amyloid precursor protein
non-CP: non-ceruloplasmin
ATP7B: ATPase copper binding pump 7B
TJs: tight junctions
HSA: human serum albumin
ATCUN: amino-terminal copper and nickel binding site
OR: Octarepeat
non-OR: non-octarepeat
XAS: X-ray absorption spectroscopy
RBMECs: rat brain microvascular endothelial cells
TEER: transendothelial electrical resistance
HPLC: high-performance liquid chromatography
RTs: retention times
LMCT: ligand–metal charge transfer
hCtr1: human copper transporter 1
CD: circular dichroism
OP: octapeptide
ESEEM: electron spin echo envelope modulation
DQ: double quantum
NQI: ^14^N quadrupolar interaction
XANES: X-ray absorption near-edge structure
EXAFS: X-ray absorption fine structure
rPrP: recombinant prion protein
HSQC: heteronuclear single quantum coherence
AMPA: α-amino-3-hydroxy-5-methyl-4-isoxazolepropionic acid receptor
STI1/AChR: stress-inducible protein 1/acethylcholine receptor
NCAM: neuronal cell adhesion molecule

## References

[ref1] Organization, W. H. Dementia. 2019. https://www.who.int/news-room/fact-sheets/detail/dementia. (accessed July 4, 2024).

[ref2] Organization, W. H. Causes of Death. 2019. https://www.who.int/news-room/fact-sheets/detail/the-top-10-causes-of-death. (accessed July 4, 2024).

[ref3] DeTure M. A.; Dickson D. W. The neuropathological diagnosis of Alzheimer’s disease. Mol. Neurodegener. 2019, 14 (1), 32 10.1186/s13024-019-0333-5. 31375134 PMC6679484

[ref4] Lopez J. A. S.; Gonzalez H. M.; Leger G. C. Alzheimer’s disease. Handb. Clin. Neurol. 2019, 167, 231–255. 10.1016/B978-0-12-804766-8.00013-3. 31753135

[ref5] Wang H.; Wang M.; Wang B.; Li M.; Chen H.; Yu X.; Yang K.; Chai Z.; Zhao Y.; Feng W. Immunogold labeling and X-ray fluorescence microscopy reveal enrichment ratios of Cu and Zn, metabolism of APP and amyloid-beta plaque formation in a mouse model of Alzheimer’s disease. Metallomics 2012, 4 (10), 1113–1118. 10.1039/c2mt20056b. 22992540

[ref6] Zhu X.; Victor T. W.; Ambi A.; Sullivan J. K.; Hatfield J.; Xu F.; Miller L. M.; Van Nostrand W. E. Copper accumulation and the effect of chelation treatment on cerebral amyloid angiopathy compared to parenchymal amyloid plaques. Metallomics 2020, 12 (4), 539–546. 10.1039/c9mt00306a. 32104807 PMC8826596

[ref7] Miller L. M.; Wang Q.; Telivala T. P.; Smith R. J.; Lanzirotti A.; Miklossy J. Synchrotron-based infrared and X-ray imaging shows focalized accumulation of Cu and Zn co-localized with beta-amyloid deposits in Alzheimer’s disease. J. Struct. Biol. 2006, 155 (1), 30–37. 10.1016/j.jsb.2005.09.004. 16325427

[ref8] Lovell M. A.; Robertson J. D.; Teesdale W. J.; Campbell J. L.; Markesbery W. R. Copper, iron and zinc in Alzheimer’s disease senile plaques. J. Neurol. Sci. 1998, 158 (1), 47–52. 10.1016/S0022-510X(98)00092-6. 9667777

[ref9] Bourassa M. W.; Leskovjan A. C.; Tappero R. V.; Farquhar E. R.; Colton C. A.; Van Nostrand W. E.; Miller L. M. Elevated copper in the amyloid plaques and iron in the cortex are observed in mouse models of Alzheimer’s disease that exhibit neurodegeneration. Biomed. Spectrosc. Imaging 2013, 2 (2), 129–139. 10.3233/BSI-130041. 24926425 PMC4051362

[ref10] Squitti R.; Catalli C.; Gigante L.; Marianetti M.; Rosari M.; Mariani S.; Bucossi S.; Mastromoro G.; Ventriglia M.; Simonelli I.; et al. Non-Ceruloplasmin Copper Identifies a Subtype of Alzheimer’s Disease (CuAD): Characterization of the Cognitive Profile and Case of a CuAD Patient Carrying an RGS7 Stop-Loss Variant. Int. J. Mol. Sci. 2023, 24 (7), 6377 10.3390/ijms24076377. 37047347 PMC10094789

[ref11] Wild K.; August A.; Pietrzik C. U.; Kins S. Structure and Synaptic Function of Metal Binding to the Amyloid Precursor Protein and its Proteolytic Fragments. Front. Mol. Neurosci. 2017, 10, 21 10.3389/fnmol.2017.00021. 28197076 PMC5281630

[ref12] Cheignon C.; Tomas M.; Bonnefont-Rousselot D.; Faller P.; Hureau C.; Collin F. Oxidative stress and the amyloid beta peptide in Alzheimer’s disease. Redox Biol. 2018, 14, 450–464. 10.1016/j.redox.2017.10.014. 29080524 PMC5680523

[ref13] Squitti R.; Ghidoni R.; Simonelli I.; Ivanova I. D.; Colabufo N. A.; Zuin M.; Benussi L.; Binetti G.; Cassetta E.; Rongioletti M.; Siotto M. Copper dyshomeostasis in Wilson disease and Alzheimer’s disease as shown by serum and urine copper indicators. J. Trace Elem. Med. Biol. 2018, 45, 181–188. 10.1016/j.jtemb.2017.11.005. 29173477

[ref14] Cuajungco M. P.; Frederickson C. J.; Bush A. I. Amyloid-β Metal Interaction and Metal Chelation. Subcell. Biochem. 2005, 235–254. 10.1007/0-387-23226-5_12. 15709482

[ref15] Singh S. K.; Balendra V.; Obaid A. A.; Esposto J.; Tikhonova M. A.; Gautam N. K.; Poeggeler B. Copper-mediated beta-amyloid toxicity and its chelation therapy in Alzheimer’s disease. Metallomics 2022, 14 (6), mfac018 10.1093/mtomcs/mfac018. 35333348

[ref16] Márquez M.; Blancas-Mejia L. M.; Campos A.; Rojas L.; Castaneda-Hernandez G.; Quintanar L. A bifunctional non-natural tetrapeptide modulates amyloid-beta peptide aggregation in the presence of Cu(ii). Metallomics 2014, 6 (12), 2189–2192. 10.1039/C4MT00257A. 25350343

[ref17] Okafor M.; Gonzalez P.; Ronot P.; El Masoudi I.; Boos A.; Ory S.; Chasserot-Golaz S.; Gasman S.; Raibaut L.; Hureau C.; et al. Development of Cu(ii)-specific peptide shuttles capable of preventing Cu-amyloid beta toxicity and importing bioavailable Cu into cells. Chem. Sci. 2022, 13 (40), 11829–11840. 10.1039/D2SC02593K. 36320914 PMC9580518

[ref18] Stefaniak E.; Plonka D.; Drew S. C.; Bossak-Ahmad K.; Haas K. L.; Pushie M. J.; Faller P.; Wezynfeld N. E.; Bal W. The N-terminal 14-mer model peptide of human Ctr1 can collect Cu(ii) from albumin. Implications for copper uptake by Ctr1. Metallomics 2018, 10 (12), 1723–1727. 10.1039/C8MT00274F. 30489586

[ref19] Shenberger Y.; Shimshi A.; Ruthstein S. EPR Spectroscopy Shows that the Blood Carrier Protein, Human Serum Albumin, Closely Interacts with the N-Terminal Domain of the Copper Transporter, Ctr1. J. Phys. Chem. B 2015, 119 (14), 4824–4830. 10.1021/acs.jpcb.5b00091. 25794362

[ref20] Ackermann K.; Wu D.; Stewart A. J.; Bode B. E. EPR spectroscopic characterisation of native Cu(II)-binding sites in human serum albumin. Dalton Trans. 2024, 53 (32), 13529–13536. 10.1039/D4DT00892H. 39072685 PMC11320662

[ref21] Kirsipuu T.; Zadoroznaja A.; Smirnova J.; Friedemann M.; Plitz T.; Tougu V.; Palumaa P. Copper(II)-binding equilibria in human blood. Sci. Rep. 2020, 10 (1), 5686 10.1038/s41598-020-62560-4. 32231266 PMC7105473

[ref22] Lutsenko S. Human copper homeostasis: a network of interconnected pathways. Curr. Opin. Chem. Biol. 2010, 14 (2), 211–217. 10.1016/j.cbpa.2010.01.003. 20117961 PMC6365103

[ref23] Roy S.; Lutsenko S. Mechanism of Cu entry into the brain: many unanswered questions. Neural Regener. Res. 2024, 19 (11), 2421–2429. 10.4103/1673-5374.393107. PMC11090436 38526278

[ref24] Sharp P. A. Ctr1 and its role in body copper homeostasis. Int. J. Biochem. Cell Biol. 2003, 35 (3), 288–291. 10.1016/S1357-2725(02)00134-6. 12531240

[ref25] You H.; Tsutsui S.; Hameed S.; Kannanayakal T. J.; Chen L.; Xia P.; Engbers J. D.; Lipton S. A.; Stys P. K.; Zamponi G. W. Abeta neurotoxicity depends on interactions between copper ions, prion protein, and N-methyl-D-aspartate receptors. Proc. Natl. Acad. Sci. U.S.A. 2012, 109 (5), 1737–1742. 10.1073/pnas.1110789109. 22307640 PMC3277185

[ref26] Huang S.; Black S. A.; Huang J.; Stys P. K.; Zamponi G. W. Mutation of copper binding sites on cellular prion protein abolishes its inhibitory action on NMDA receptors in mouse hippocampal neurons. Mol. Brain 2021, 14 (1), 117 10.1186/s13041-021-00828-0. 34281567 PMC8287767

[ref27] Gasperini L.; Meneghetti E.; Pastore B.; Benetti F.; Legname G. Prion protein and copper cooperatively protect neurons by modulating NMDA receptor through S-nitrosylation. Antioxid. Redox Signaling 2015, 22 (9), 772–784. 10.1089/ars.2014.6032. PMC4361008 25490055

[ref28] Traynelis S. F.; Wollmuth L. P.; McBain C. J.; Menniti F. S.; Vance K. M.; Ogden K. K.; Hansen K. B.; Yuan H.; Myers S. J.; Dingledine R. Glutamate receptor ion channels: structure, regulation, and function. Pharmacol. Rev. 2010, 62 (3), 405–496. 10.1124/pr.109.002451. 20716669 PMC2964903

[ref29] Paoletti P.; Bellone C.; Zhou Q. NMDA receptor subunit diversity: impact on receptor properties, synaptic plasticity and disease. Nat. Rev. Neurosci. 2013, 14 (6), 383–400. 10.1038/nrn3504. 23686171

[ref30] Ultanir S. K.; Kim J. E.; Hall B. J.; Deerinck T.; Ellisman M.; Ghosh A. Regulation of spine morphology and spine density by NMDA receptor signaling in vivo. Proc. Natl. Acad. Sci. U.S.A. 2007, 104 (49), 19553–19558. 10.1073/pnas.0704031104. 18048342 PMC2148327

[ref31] Brown D. R.; Qin K.; Herms J. W.; Madlung A.; Manson J.; Strome R.; Fraser P. E.; Kruck T.; von Bohlen A.; Schulz-Schaeffer W.; et al. The cellular prion protein binds copper in vivo. Nature 1997, 390 (6661), 684–687. 10.1038/37783. 9414160

[ref32] Herms J.; Tings T.; Gall S.; Madlung A.; Giese A.; Siebert H.; Schürmann P.; Windl O.; Brose N.; Kretzschmar H. Evidence of Presynaptic Location and Function of the Prion Protein. J. Neurosci. 1999, 19 (20), 8866–8875. 10.1523/JNEUROSCI.19-20-08866.1999. 10516306 PMC6782778

[ref33] Walter E. D.; Chattopadhyay M.; Millhauser G. L. The affinity of copper binding to the prion protein octarepeat domain: evidence for negative cooperativity. Biochemistry 2006, 45 (43), 13083–13092. 10.1021/bi060948r. 17059225 PMC2905157

[ref34] Chattopadhyay M.; Walter E. D.; Newell D. J.; Jackson P. J.; Aronoff-Spencer E.; Peisach J.; Gerfen G. J.; Bennett B.; Antholine W. E.; Millhauser G. L. The octarepeat domain of the prion protein binds Cu(II) with three distinct coordination modes at pH 7.4. J. Am. Chem. Soc. 2005, 127 (36), 12647–12656. 10.1021/ja053254z. 16144413 PMC2909831

[ref35] Viles J. H.; Cohen F. E.; Prusiner S. B.; Goodin D. B.; Wright P. E.; Dyson H. J. Copper binding to the prion protein: structural implications of four identical cooperative binding sites. Proc. Natl. Acad. Sci. U.S.A. 1999, 96 (5), 2042–2047. 10.1073/pnas.96.5.2042. 10051591 PMC26733

[ref36] Grande-Aztatzi R.; Rivillas-Acevedo L.; Quintanar L.; Vela A. Structural models for Cu(II) bound to the fragment 92–96 of the human prion protein. J. Phys. Chem. B 2013, 117 (3), 789–799. 10.1021/jp310000h. 23240680

[ref37] Rivillas-Acevedo L.; Grande-Aztatzi R.; Lomeli I.; Garcia J. E.; Barrios E.; Teloxa S.; Vela A.; Quintanar L. Spectroscopic and electronic structure studies of copper(II) binding to His111 in the human prion protein fragment 106–115: evaluating the role of protons and methionine residues. Inorg. Chem. 2011, 50 (5), 1956–1972. 10.1021/ic102381j. 21261254

[ref38] Klewpatinond M.; Davies P.; Bowen S.; Brown D. R.; Viles J. H. Deconvoluting the Cu2+ binding modes of full-length prion protein. J. Biol. Chem. 2008, 283 (4), 1870–1881. 10.1074/jbc.M708472200. 18042548

[ref39] Jones C. E.; Klewpatinond M.; Abdelraheim S. R.; Brown D. R.; Viles J. H. Probing copper2+ binding to the prion protein using diamagnetic nickel2+ and 1H NMR: the unstructured N terminus facilitates the coordination of six copper2+ ions at physiological concentrations. J. Mol. Biol. 2005, 346 (5), 1393–1407. 10.1016/j.jmb.2004.12.043. 15713489

[ref40] Evans E. G.; Pushie M. J.; Markham K. A.; Lee H. W.; Millhauser G. L. Interaction between Prion Protein’s Copper-Bound Octarepeat Domain and a Charged C-Terminal Pocket Suggests a Mechanism for N-Terminal Regulation. Structure 2016, 24 (7), 1057–1067. 10.1016/j.str.2016.04.017. 27265848 PMC4938727

[ref41] Schilling K. M.; Tao L.; Wu B.; Kiblen J. T. M.; Ubilla-Rodriguez N. C.; Pushie M. J.; Britt R. D.; Roseman G. P.; Harris D. A.; Millhauser G. L. Both N-Terminal and C-Terminal Histidine Residues of the Prion Protein Are Essential for Copper Coordination and Neuroprotective Self-Regulation. J. Mol. Biol. 2020, 432 (16), 4408–4425. 10.1016/j.jmb.2020.05.020. 32473880 PMC7387163

[ref42] Posadas Y.; Lopez-Guerrero V. E.; Segovia J.; Perez-Cruz C.; Quintanar L. Dissecting the copper bioinorganic chemistry of the functional and pathological roles of the prion protein: Relevance in Alzheimer’s disease and cancer. Curr. Opin. Chem. Biol. 2022, 66, 102098 10.1016/j.cbpa.2021.102098. 34768088

[ref43] Banks W. A. Drug delivery to the brain in Alzheimer’s disease: consideration of the blood-brain barrier. Adv. Drug Delivery Rev. 2012, 64 (7), 629–639. 10.1016/j.addr.2011.12.005. PMC3389492 22202501

[ref44] Yao J. F.; Yang H.; Zhao Y. Z.; Xue M. Metabolism of Peptide Drugs and Strategies to Improve their Metabolic Stability. Curr. Drug Metab. 2018, 19 (11), 892–901. 10.2174/1389200219666180628171531. 29956618

[ref45] Luissint A. C.; Artus C.; Glacial F.; Ganeshamoorthy K.; Couraud P. O. Tight junctions at the blood brain barrier: physiological architecture and disease-associated dysregulation. Fluids Barriers CNS 2012, 9 (1), 23 10.1186/2045-8118-9-23. 23140302 PMC3542074

[ref46] González-Mariscal L.; Nava P.; Hernandez S. Critical role of tight junctions in drug delivery across epithelial and endothelial cell layers. J. Membr. Biol. 2005, 207 (2), 55–68. 10.1007/s00232-005-0807-y. 16477528

[ref47] González-Mariscal L.; Posadas Y.; Miranda J.; Uc P. Y.; Ortega-Olvera J. M.; Hernandez S. Strategies that Target Tight Junctions for Enhanced Drug Delivery. Curr. Pharm. Des. 2016, 22 (35), 5313–5346. 10.2174/1381612822666160720163656. 27510485

[ref48] Díaz-Coránguez M.; Segovia J.; Lopez-Ornelas A.; Puerta-Guardo H.; Ludert J.; Chavez B.; Meraz-Cruz N.; Gonzalez-Mariscal L. Transmigration of neural stem cells across the blood brain barrier induced by glioma cells. PLoS One 2013, 8 (4), e60655 10.1371/journal.pone.0060655. 23637756 PMC3618035

[ref49] Nava P.; Lopez S.; Arias C. F.; Islas S.; Gonzalez-Mariscal L. The rotavirus surface protein VP8 modulates the gate and fence function of tight junctions in epithelial cells. J. Cell Sci. 2004, 117 (Pt 23), 5509–5519. 10.1242/jcs.01425. 15494377

[ref50] Gonzalez-Mariscal L.; Nava-Dominguez P. Employment of rotavirus proteins, derived proteins and peptides for the modulation of tissue permeability. U.S. Patent US 8,383,129 B2, 2006.

[ref51] Mentlein R. Cell-surface peptidases. Int. Rev. Cytol. 2004, 235, 165–213. 10.1016/S0074-7696(04)35004-7. 15219783 PMC7126636

[ref52] Ackerman C. M.; Chang C. J. Copper signaling in the brain and beyond. J. Biol. Chem. 2018, 293 (13), 4628–4635. 10.1074/jbc.R117.000176. 29084848 PMC5880129

[ref53] Lei P.; Ayton S.; Bush A. I. The essential elements of Alzheimer’s disease. J. Biol. Chem. 2021, 296, 100105 10.1074/jbc.REV120.008207. 33219130 PMC7948403

[ref54] Zhang C.; Dischler A.; Glover K.; Qin Y. Neuronal signalling of zinc: from detection and modulation to function. Open Biol. 2022, 12 (9), 220188 10.1098/rsob.220188. 36067793 PMC9448499

[ref55] Bagheri S.; Squitti R.; Haertle T.; Siotto M.; Saboury A. A. Role of Copper in the Onset of Alzheimer’s Disease Compared to Other Metals. Front. Aging Neurosci. 2018, 9, 446 10.3389/fnagi.2017.00446. 29472855 PMC5810277

[ref56] Rodríguez E. E.; Arcos-Lopez T.; Trujano-Ortiz L. G.; Fernandez C. O.; Gonzalez F. J.; Vela A.; Quintanar L. Role of N-terminal methionine residues in the redox activity of copper bound to alpha-synuclein. JBIC, J. Biol. Inorg. Chem. 2016, 21 (5–6), 691–702. 10.1007/s00775-016-1376-5. 27422629

[ref57] Gonzalez P.; Bossak K.; Stefaniak E.; Hureau C.; Raibaut L.; Bal W.; Faller P. N-Terminal Cu-Binding Motifs (Xxx-Zzz-His, Xxx-His) and Their Derivatives: Chemistry, Biology and Medicinal Applications. Chemistry 2018, 24 (32), 8029–8041. 10.1002/chem.201705398. 29336493 PMC6152890

[ref58] Masters C. L.; Simms G.; Weinman N. A.; Multhaup G.; McDonald B. L.; Beyreuther K. Amyloid plaque core protein in Alzheimer disease and Down syndrome. Proc. Natl. Acad. Sci. U.S.A. 1985, 82 (12), 4245–4249. 10.1073/pnas.82.12.4245. 3159021 PMC397973

[ref59] Masters C. L.; Multhaup G.; Simms G.; Pottgiesser J.; Martins R. N.; Beyreuther K. Neuronal origin of a cerebral amyloid: neurofibrillary tangles of Alzheimer’s disease contain the same protein as the amyloid of plaque cores and blood vessels. EMBO J. 1985, 4 (11), 2757–2763. 10.1002/j.1460-2075.1985.tb04000.x. 4065091 PMC554575

[ref60] Portelius E.; Bogdanovic N.; Gustavsson M. K.; Volkmann I.; Brinkmalm G.; Zetterberg H.; Winblad B.; Blennow K. Mass spectrometric characterization of brain amyloid beta isoform signatures in familial and sporadic Alzheimer’s disease. Acta Neuropathol. 2010, 120 (2), 185–193. 10.1007/s00401-010-0690-1. 20419305 PMC3568930

[ref61] Faller P.; Hureau C. Bioinorganic chemistry of copper and zinc ions coordinated to amyloid-beta peptide. Dalton Trans. 2009, (7), 1080–1094. 10.1039/B813398K. 19322475

[ref62] Alies B.; Renaglia E.; Rozga M.; Bal W.; Faller P.; Hureau C. Cu(II) affinity for the Alzheimer’s peptide: tyrosine fluorescence studies revisited. Anal. Chem. 2013, 85 (3), 1501–1508. 10.1021/ac302629u. 23249207

[ref63] Wezynfeld N. E.; Stefaniak E.; Stachucy K.; Drozd A.; Plonka D.; Drew S. C.; Krezel A.; Bal W. Resistance of Cu(Abeta4–16) to Copper Capture by Metallothionein-3 Supports a Function for the Abeta4–42 Peptide as a Synaptic Cu(II) Scavenger. Angew. Chem., Int. Ed. 2016, 55 (29), 8235–8238. 10.1002/anie.201511968. 27238224

[ref64] Mital M.; Wezynfeld N. E.; Fraczyk T.; Wiloch M. Z.; Wawrzyniak U. E.; Bonna A.; Tumpach C.; Barnham K. J.; Haigh C. L.; Bal W.; et al. A Functional Role for Abeta in Metal Homeostasis? N-Truncation and High-Affinity Copper Binding. Angew. Chem., Int. Ed. 2015, 54 (36), 10460–10464. 10.1002/anie.201502644. 26178596

[ref65] Ekanayake R. S. K.; Streltsov V. A.; Best S. P.; Chantler C. T. Nanostructure and dynamics of N-truncated copper amyloid-beta peptides from advanced X-ray absorption fine structure. IUCrJ 2024, 11 (Pt 3), 325–346. 10.1107/S2052252524001830. PMC11067746 38602752

[ref66] Mital M.; Bal W.; Fraczyk T.; Drew S. C. Interplay between Copper, Neprilysin, and N-Truncation of beta-Amyloid. Inorg. Chem. 2018, 57 (11), 6193–6197. 10.1021/acs.inorgchem.8b00391. 29774745

[ref67] Posadas Y.; Parra-Ojeda L.; Perez-Cruz C.; Quintanar L. Amyloid beta Perturbs Cu(II) Binding to the Prion Protein in a Site-Specific Manner: Insights into Its Potential Neurotoxic Mechanisms. Inorg. Chem. 2021, 60 (12), 8958–8972. 10.1021/acs.inorgchem.1c00846. 34043332

[ref68] Burns C. S.; Aronoff-Spencer E.; Dunham C. M.; Lario P.; Avdievich N. I.; Antholine W. E.; Olmstead M. M.; Vrielink A.; Gerfen G. J.; Peisach J.; et al. Molecular features of the copper binding sites in the octarepeat domain of the prion protein. Biochemistry 2002, 41 (12), 3991–4001. 10.1021/bi011922x. 11900542 PMC2905306

[ref69] Peisach J.; Blumberg W. E. Structural implications derived from the analysis of electron paramagnetic resonance spectra of natural and artificial copper proteins. Arch. Biochem. Biophys. 1974, 165 (2), 691–708. 10.1016/0003-9861(74)90298-7. 4374138

[ref70] Colaneri M. J.; Peisach J. Electron Spin-Echo Envelope Modulation Studies Of 14N In Biological Systems. In Biomedical EPR, Part A: Free Radicals, Metals, Medicine, and Physiology; Eaton S. R.; Eaton G. R.; Berliner L. J., Eds.; Springer, 2005; pp 455–491.

[ref71] Binolfi A.; Rodriguez E. E.; Valensin D.; D’Amelio N.; Ippoliti E.; Obal G.; Duran R.; Magistrato A.; Pritsch O.; Zweckstetter M.; et al. Bioinorganic chemistry of Parkinson’s disease: structural determinants for the copper-mediated amyloid formation of alpha-synuclein. Inorg. Chem. 2010, 49 (22), 10668–10679. 10.1021/ic1016752. 20964419

[ref72] Gaur A.; Klysubun W.; Nitin Nair N.; Shrivastava B. D.; Prasad J.; Srivastava K. XAFS study of copper(II) complexes with square planar and square pyramidal coordination geometries. J. Mol. Struct. 2016, 1118, 212–218. 10.1016/j.molstruc.2016.04.008.

[ref73] Strange R. W.; Alagna L.; Durham P.; Hasnain S. S. An understanding of the x-ray absorption near-edge structure of copper(II) imidazole complexes. J. Am. Chem. Soc. 1990, 112 (11), 4265–4268. 10.1021/ja00167a022.

[ref74] Millhauser G. L. Copper and the prion protein: methods, structures, function, and disease. Annu. Rev. Phys. Chem. 2007, 58, 299–320. 10.1146/annurev.physchem.58.032806.104657. 17076634 PMC2904554

[ref75] Dunn K. W.; Kamocka M. M.; McDonald J. H. A practical guide to evaluating colocalization in biological microscopy. Am. J. Physiol.: Cell Physiol. 2011, 300 (4), C723–742. 10.1152/ajpcell.00462.2010. 21209361 PMC3074624

[ref76] O’Keeffe E.; Campbell M. Modulating the paracellular pathway at the blood-brain barrier: current and future approaches for drug delivery to the CNS. Drug Discovery Today:Technol. 2016, 20, 35–39. 10.1016/j.ddtec.2016.07.008. 27986221

[ref77] Behar A. E.; Sabater L.; Baskin M.; Hureau C.; Maayan G. A. Water-Soluble Peptoid Chelator that Can. Remove Cu(2+) from Amyloid-beta Peptides and Stop the Formation of Reactive Oxygen Species Associated with Alzheimer’s Disease. Angew. Chem., Int. Ed. 2021, 60 (46), 24588–24597. 10.1002/anie.202109758. 34510664

[ref78] Gough M.; Parr-Sturgess C.; Parkin E. Zinc metalloproteinases and amyloid Beta-Peptide metabolism: the positive side of proteolysis in Alzheimer’s disease. Biochem. Res. Int. 2011, 2011, 721463 10.1155/2011/721463. 21152187 PMC2989646

[ref79] Portbury S. D.; Adlard P. A. Zinc Signal in Brain Diseases. Int. J. Mol. Sci. 2017, 18 (12), 2506 10.3390/ijms18122506. 29168792 PMC5751109

[ref80] Lutsenko S.; LeShane E. S.; Shinde U. Biochemical basis of regulation of human copper-transporting ATPases. Arch. Biochem. Biophys. 2007, 463 (2), 134–148. 10.1016/j.abb.2007.04.013. 17562324 PMC2025638

[ref81] Maiti B. K.; Govil N.; Kundu T.; Moura J. J. G. Designed Metal-ATCUN Derivatives: Redox- and Non-redox-Based Applications Relevant for Chemistry, Biology, and Medicine. iScience 2020, 23 (12), 101792 10.1016/j.isci.2020.101792. 33294799 PMC7701195

[ref82] Binolfi A.; Rasia R. M.; Bertoncini C. W.; Ceolin M.; Zweckstetter M.; Griesinger C.; Jovin T. M.; Fernandez C. O. Interaction of alpha-synuclein with divalent metal ions reveals key differences: a link between structure, binding specificity and fibrillation enhancement. J. Am. Chem. Soc. 2006, 128 (30), 9893–9901. 10.1021/ja0618649. 16866548

[ref83] Esmieu C.; Ferrand G.; Borghesani V.; Hureau C. Impact of N-Truncated Abeta Peptides on Cu- and Cu(Abeta)-Generated ROS: Cu(I) Matters!. Chem. - Eur. J. 2021, 27 (5), 1777–1786. 10.1002/chem.202003949. 33058356

[ref84] Greenough M. A.; Camakaris J.; Bush A. I. Metal dyshomeostasis and oxidative stress in Alzheimer’s disease. Neurochem. Int. 2013, 62 (5), 540–555. 10.1016/j.neuint.2012.08.014. 22982299

[ref85] Walker L. C.; Jucker M. The prion principle and Alzheimer’s disease. Science 2024, 385 (6715), 1278–1279. 10.1126/science.adq5252. 39298592 PMC11492928

[ref86] Velayos J. L.; Irujo A.; Cuadrado-Tejedor M.; Paternain B.; Moleres F. J.; Ferrer V. The cellular prion protein and its role in Alzheimer disease. Prion 2009, 3 (2), 110–117. 10.4161/pri.3.2.9135. 19556894 PMC2712608

[ref87] Takahashi R. H.; Yokotsuka M.; Tobiume M.; Sato Y.; Hasegawa H.; Nagao T.; Gouras G. K. Accumulation of cellular prion protein within beta-amyloid oligomer plaques in aged human brains. Brain Pathol. 2021, 31 (5), e12941 10.1111/bpa.12941. 33624334 PMC8412093

[ref88] Takahashi R. H.; Tobiume M.; Sato Y.; Sata T.; Gouras G. K.; Takahashi H. Accumulation of cellular prion protein within dystrophic neurites of amyloid plaques in the Alzheimer’s disease brain. Neuropathology 2011, 31 (3), 208–214. 10.1111/j.1440-1789.2010.01158.x. 21062360

[ref89] Ferrer I.; Blanco R.; Carmona M.; Puig B.; Ribera R.; Rey M. J.; Ribalta T. Prion protein expression in senile plaques in Alzheimer’s disease. Acta Neuropathol. 2001, 101 (1), 49–56. 10.1007/s004010000271. 11194941

[ref90] Dohler F.; Sepulveda-Falla D.; Krasemann S.; Altmeppen H.; Schluter H.; Hildebrand D.; Zerr I.; Matschke J.; Glatzel M. High molecular mass assemblies of amyloid-beta oligomers bind prion protein in patients with Alzheimer’s disease. Brain 2014, 137 (Pt 3), 873–886. 10.1093/brain/awt375. 24519981

[ref91] Beland M.; Bedard M.; Tremblay G.; Lavigne P.; Roucou X. Abeta induces its own prion protein N-terminal fragment (PrPN1)-mediated neutralization in amorphous aggregates. Neurobiol. Aging 2014, 35 (7), 1537–1548. 10.1016/j.neurobiolaging.2014.02.001. 24602510

[ref92] Foley A. R.; Roseman G. P.; Chan K.; Smart A.; Finn T. S.; Yang K.; Lokey R. S.; Millhauser G. L.; Raskatov J. A. Evidence for aggregation-independent, PrP(C)-mediated Abeta cellular internalization. Proc. Natl. Acad. Sci. U.S.A. 2020, 117 (46), 28625–28631. 10.1073/pnas.2009238117. 33139554 PMC7682355

[ref93] Lannfelt L.; Blennow K.; Zetterberg H.; Batsman S.; Ames D.; Harrison J.; Masters C. L.; Targum S.; Bush A. I.; Murdoch R.; et al. Safety, efficacy, and biomarker findings of PBT2 in targeting Abeta as a modifying therapy for Alzheimer’s disease: a phase IIa, double-blind, randomised, placebo-controlled trial. Lancet Neurol. 2008, 7 (9), 779–786. 10.1016/S1474-4422(08)70167-4. 18672400

[ref94] Faux N. G.; Ritchie C. W.; Gunn A.; Rembach A.; Tsatsanis A.; Bedo J.; Harrison J.; Lannfelt L.; Blennow K.; Zetterberg H.; et al. PBT2 rapidly improves cognition in Alzheimer’s Disease: additional phase II analyses. J. Alzheimer’s Dis. 2010, 20 (2), 509–516. 10.3233/JAD-2010-1390. 20164561

[ref95] Kenche V. B.; Zawisza I.; Masters C. L.; Bal W.; Barnham K. J.; Drew S. C. Mixed ligand Cu2+ complexes of a model therapeutic with Alzheimer’s amyloid-beta peptide and monoamine neurotransmitters. Inorg. Chem. 2013, 52 (8), 4303–4318. 10.1021/ic302289r. 23537393

[ref96] Mital M.; Zawisza I. A.; Wiloch M. Z.; Wawrzyniak U. E.; Kenche V.; Wroblewski W.; Bal W.; Drew S. C. Copper Exchange and Redox Activity of a Prototypical 8-Hydroxyquinoline: Implications for Therapeutic Chelation. Inorg. Chem. 2016, 55 (15), 7317–7319. 10.1021/acs.inorgchem.6b00832. 27409140

[ref97] Drew S. C. Chelator PBT2 Forms a Ternary Cu(2+) Complex with beta-Amyloid That Has High Stability but Low Specificity. Int. J. Mol. Sci. 2023, 24 (11), 9267 10.3390/ijms24119267. 37298218 PMC10252752

[ref98] Drew S. C. The Case for Abandoning Therapeutic Chelation of Copper Ions in Alzheimer’s Disease. Front. Neurosci. 2017, 11, 317 10.3389/fnins.2017.00317. 28626387 PMC5455140

[ref99] Bush A. I.; Tanzi R. E. Therapeutics for Alzheimer’s disease based on the metal hypothesis. Neurotherapeutics 2008, 5 (3), 421–432. 10.1016/j.nurt.2008.05.001. 18625454 PMC2518205

