# Supplementary material for: A Copper-Binding Peptide with Therapeutic Potential against Alzheimer′s Disease: From the Blood–Brain Barrier to Metal Competition
Source: ACS Chem Neurosci. 2024 Dec 26;16(2):241–61. doi: 10.1021/acschemneuro.4c00796 (PMC11741003; doi:10.1021/acschemneuro.4c00796)
Supplement: Supplementary file 1 — cn4c00796_si_001.pdf [file cn4c00796_si_001.pdf]

Supporting information

A copper-binding peptide with therapeutic potential against Alzheimer´s disease: From the blood-brain barrier to metal competition

Victor E. Lopez-Guerrero,[1,2] Yanahi Posadas,[1,3] Carolina Sánchez-López,[4] Amanda Smart,[5] Jael Miranda,[1] Kevin Singewald,[5] Yamir Bandala,[2] Eusebio Juaristi,[2,6] Christophe Den Auwer,[7] Claudia Perez-Cruz,[3] Lorenza González-Mariscal,[1] Glenn Millhauser,[5,*] Jose Segovia,[1,*] Liliana Quintanar.[2,4,*]

[1]Department of Physiology, Biophysics, and Neuroscience, Center for Research and Advanced Studies (Cinvestav), Mexico City, 07350, Mexico.
[2]Department of Chemistry, Center for Research and Advanced Studies (Cinvestav), Mexico City, 07350, Mexico.
[3]Department of Pharmacology, Center for Research and Advanced Studies (Cinvestav), Mexico City, 07350, Mexico.
[4]Center for Research in Aging, Center for Research and Advanced Studies (Cinvestav), Mexico City, 14330, Mexico.
[5]Department of Chemistry and Biochemistry, University of California, 1156, Santa Cruz, USA.
[6]El Colegio Nacional, Mexico City, 06020, Mexico.
[7]Université Côte d´Azur, CNRS, Institute de Chimie de Nice, Nice, 06108, France.

Corresponding authors:

* Liliana Quintanar. Center for Research and Advanced Studies (Cinvestav), Mexico, City, Mexico.
E-mail: lilianaq@cinvestav.mx
* Jose Segovia. Center for Research and Advanced Studies (Cinvestav), Mexico, City, Mexico.
E-mail: jsegovia@fisio.cinvestav.mx
* Glenn Millhauser. Department of Chemistry and Biochemistry, University of California, Santa Cruz, California, USA.
E-mail: glennm@ucsc.edu

Supplementary information

Materials and methods section. 3

Figure S1. P10 opens the BBB. 8

Figure S2. Chromatographic profile of TP samples incubated in the BBB model and identification of degradation products 8

Figure S3. Bifunctional properties of original TP (MDDWAib) and TP* variant (DMDWAib). 9

Table S2. EPR parameters for the titration of Cu^{2+}-hCtr1 and Cu^{2+}-HSA complexes with TP* 10

Figure S4. Comparative CD spectra from Cu^{2+}-ATCUN sites 10

Table S3. CD parameters of control Cu^{2+}-ATCUN sites of hCtr1(1–14), HSA, and Aβ(4–16) 10

Figure S5. Titration of Cu^{2+}-TP* complex with HSA followed by CD 11

Table S4. CD parameters for the final point of the titrations of Cu^{2+}-TP* with ATCUN species. 11

Figure S6. Competition for Cu^{2+} between TP* and the non-OR sites of the PrP. 13

Figure S7. Comparative EPR and CD spectra of reported ternary TP*-Cu^{2+}-PrP complexes 14

Table S5. EPR parameters for reported ternary TP*-Cu^{2+}-PrP complexes. 14

Table S6. CD parameters for reported ternary TP*-Cu^{2+}-PrP complexes. 14

Figure S8. EPR simulation of the ternary TP*-Cu^{2+}-OP complex. 15

Table S7. Spin Hamiltonian Parameters for the EPR spectrum simulation of the ternary TP*-Cu^{2+}-OP complex. 15

Figure S9. Peisach-Blumberg correlations for the Cu^{2+}-OP, TP*-Cu^{2+}-OP, and Cu^{2+}-TP* complexes 16

Figure S10. ESEEM comparison between Cu^{2+}-OP and ternary TP*-Cu^{2+}-OP spectra. 17

Figure S11. Comparative EPR spectra of the Cu^{2+}-TP* and ternary TP*-Cu^{2+}-OP complexes prepared in ^{16}O water and ^{17}O enriched water. 18

Table S8. EPR parameters for the TP*-Cu^{2+} complex prepared in ^{16}O water and ^{17}O enriched water. 18

Table S9. EPR parameters for the TP*-Cu^{2+}-OP ternary complex prepared in ^{16}O water and ^{17}O enriched water. 18

Figure S12. Comparison of EXAFS and FT of EXAFS spectra of Cu^{2+}-OP, Cu^{2+}-TP* and TP*-Cu^{2+}-OP complexes. 19

Table S11. EXAFS simulation parameters using a 3N1O1S coordination mode for the TP*-Cu^{2+}-OP complex. 19

Figure S13. EXAFS fit of ternary TP*-Cu^{2+}-OP complex using a 3N1O coordination sphere. 19

Table S11. EXAFS simulation parameters using a 3N1O coordination mode for the TP*-Cu^{2+}-OP complex. 20

Figure S14. ESEEM comparison between Cu^{2+}-rPrP and ternary TP*-Cu^{2+}-OP spectra. 20

Figure S15. ESEEM comparison between ternary TP*-Cu^{2+}-rPrP and ternary TP*-Cu^{2+}-OP spectra. 21

Figure S16. ^{1}H-^{15}N HSQC intensity ratio of rPrP + 1.0 equiv of TP* compared to rPrP. 21

Figure S17. Differentiation of SK-N-SH cell line using RA 22

Figure S18. Expression of NMDAR (GluN1-1a/GluN2B subunits) and PrP^{C} in the RA-differentiated SK-N-SH cell line. 23

Figure S19. Effect of Cu^{2+} in the colocalization of PrP^{C} and NMDAR. 24

Figure S21. Multiple comparisons of the effect of TP* in the Cu^{2+}-dependent colocalization of PrP^{C} and NMDAR. 25

Figure S22. Expression of PrP^{C} in the different experimental condition. 26

References 26

Materials and methods section.

Materials. (Z)-ethyl 2-cyano-3-hydroxyacrylate potassium salt (K-Oxyme, 776874), 9-Fluorenylmethoxycarbonyl (Fmoc) protected amino acids, Human serum albumin (>96% purity) (A1653), Fmoc-Rink amide MBHA resin (Novabiochem, 8.55003), Wang resin (13609), Glycine (G8898), 2-Propanol (I9516), 2-mercaptoethanol (M6250), acetic acid (695092), piperidine (104094), 1,8-Diazabicyclo(5,4,0)undec-7-ene (DBU) (33482), N,N′-Diisopropylcarbodiimide (DIC) (D125407), 1,2 ethanedithiol (EDT) (02390), trifluoroacetic acid (TFA) (T6508), 4-ethylmorpholine (NEM) (109932), 3-(N-morfolino)propanesulfonic acid (MOPS) (M1254), 2-(N-Morpholino)ethanesulfonic acid (MES) (M3671), Triisopropylsilane (TIS) (233781), glycerol (G7893), , copper sulphate $CuSO_4•5H_2O$ (C3036), copper chloride (222011), retinoic acid (R2625), Collagenase type 2 (C6885), Percoll gradient (P1644), Dulbecco's Modified Eagle's Medium/Nutrient Mixture F-12 Ham DMEM/F12 (D0697), L-glutamine (35050061), Collagenase/Dispase (11097113001), Puromycin 1 mg/mL (P9620), 8-(4-Chlorophenylthiol) adenosine 39, 59-cyclic monophosphate sodium salt (C3912), the c-AMP phosphodiesterase-4-specific inhibitor RO-20-1724 (B8279), hydrocortisone (HO888), Isopropyl ß-D-1-thiogalactopyranoside (IPTG) (PHG0010), Complete Protease inhibitor (Roche, 11697498001), guanidinium chloride (G4505), ammonium chloride isotopic labelled$^{15}NH_4Cl$, Sodium Acetate (58750) and antibodies—anti-PrP^C clone 8H4, purified from hybridoma cell culture (P0110) and anti-NMDAR2B antibody (AB1557)—were purchased from Sigma-Aldrich. Millicell inserts (PIEP 15R 48), centrifugal filters (UFC5003), and zinc chloride $ZnSO_2•7H_2O$ (1.08883) were purchase from Merck Millipore. N,N-dimethylformamide (DMF) (1200), dichloromethane (DCM) (1725) and 2-Propanol (IPA) (0405) reactive grade were purchased from Meyer's chemical reactives. Methanol (MeOH), acetonitrile (ACN), and isopropanol (IPA) HPLC grade were purchase from J. T. Baker. Water used in all the experiments was obtained by the Elix/Milli-Q water purification system from Merck Millipore with a resistivity > 18 mΩ cm^{-1}. For cell culture Glutamax (35050079) and fungizone 1 mg/mL (P9620) were purchased from Invitrogen. Fetal bovine serum (Gibco, 16000044) and penicillin/streptomycin 100 U/mL/10000 µg/mL (Gibco, 15140122), were purchased from Thermo Fisher Scientific. Heparin used was acquired from Pisa S.A. de C.V. (177M90 SSA IV), penicillin 100 U/mL, streptomycin 100 mg/mL, gentamicin 1 mg/mL from Schering-Plough (63671 SSAIV), kanamycin from Bistrol-Myers Squibb (53508 SSA IV). Materials for western blotting such as 30% acrylamide/Bis solution 29:1 (1610156) and Immunoblot PVDF membranes (1620177) were purchased from BioRad. Sodium dodecyl sulfate (SDS) (15525–017). Antibodies employed for immunoblotting: anti PrP^C clone 8H4, purified from hybridoma cell culture (Merck, P0110), anti GluN2B (Merck, AB1557), anti GluN1 (Merck, 05-432), anti-mouse HRP (Invitrogen, 62-6520), anti-rabbit HRP (Invitrogen 65, 6120), anti-β-actin HRP conjugated (Sigma Aldrich, A3854). Membranes were revealed using a Western-Lightning Plus-ECL (PerkinElmer, Inc. NEL104001EA). Materials for immunofluorescence such as 1% Bovine Serum Albumin IgG-Free (BSA) was purchased from Jackson ImmunoResearch, (001-000-162) and Vectashield with or without DAPI from Vector laboratories (H-1200). Secondary antibodies such as Alexa Fluor 594: Donkey Anti-mouse (A21203), and Alexa Fluor 488: Donkey Anti-rabbit (A11008) were purchased from Sigma Aldrich.

Animals and ethical statement. Wistar rats were obtained from Cinvestav vivarium after approval of the Local Committee of Ethics on Animal Experimentation (CICUAL Cinvestav). Animals were treated following the regulations established in the Mexican Official Norm for the Use and Care of Laboratory Animals (Permit number: NOM-062-ZOO-1999) and all efforts were made to minimize suffering. Rats were euthanized by CO_2 inhalation.

Peptide synthesis, purification, and characterization. All the peptides used in this study were synthesized using the standard protocol for the solid-phase synthesis (SPPS) with Fmoc-strategy.[1] TP* and all the fragments used for spectroscopic characterization—PrP(60–91), PrP(92–99), PrP(106–115), OP, Ctr1(1–14), Aβ(4–16)—were synthesized with an amide group at C-terminal. PrP(60–91), PrP(92–99), PrP(106–115), and OP were acetylated at the N-terminal, while TP*, Ctr1(1–14) and Aβ(4–16) were synthesized with the free-amino group. Sequences are shown in Table S1. P10 was synthesized with the free-amino group and a free-carboxylate group at C-terminal. Fmoc-amino acids were activated using a coupling reaction with Oxyma pure/DIC-containing DMF solution. Fmoc group was removed using a solution with 20% piperidine and 2% DBU in DMF. Peptides were cleaved from the solid support using a cocktail of composition: 92.5% TFA, 2.5% TIS, 2.5% EDT, and 2.5% water. The crudes of synthesis were purified by high performance liquid chromatography (HPLC) using a semi-preparative column Pursuit C18 (10 µm, 25 x 10 mm) on a Waters HPLC system with a photodiode array (PDA) UV-vis detector. Peptides were eluted using gradient methods, which were optimized for each sequence. Pure peptides were lyophilized and storage at –20ºC. The peptides were characterized using mass spectrometry. Peptide purity was assayed by HPLC using an analytical column Zorbax Eclipse C18 (4.5 µm, 150 x 4.5 mm). All the peptides used in this study have a purity ≥ 95%.

Blood-brain barrier (BBB) *in vitro* model: The BBB in vitro model used in this study is based on the primary culture of rat brain microvascular endothelial cells (RBMECs) grown on top of an insert with a semipermeable filter and placed in a multiwell plate. In the basal compartment, conditioned media (CM) obtained from a primary culture of rat cortical astrocytes was added to induce the sealing of the BBB. The formation and sealing of TJs was demonstrated by continuously measuring the transendothelial resistance (TEER).

RBMECs were isolated from the meninges of 2-day-old Wistar rat brains as previously described. The cerebral tissue was cut into small pieces and digested using 1 mg/mL of Collagenase type 2 at 37 °C for 75 minutes. Myelin was separated by centrifugation at 1000 g for 20 minutes in a solution of 20% albumin. Then, a second digestion was done using 1 mg of Collagenase/Dispase at 37 °C for 50 minutes. The fragmented microvasculature was centrifuged with a Percoll gradient for 10 minutes at 1000 g. The RBMECs were grown in DMEM/F12 media supplemented with 20% of fetal bovine serum, 500 ng/mL bFGF (507361), and 0.2 M of Glutamax, 8 mg/mL of heparin penicillin 100 U/mL, streptomycin 100 mg/mL, gentamicin 1 mg/mL, kanamycin 1 mg/mL and fungizone 1 mg/mL. The RBMECs were grown in a media with puromycin 1 mg/mL for the first two days to remove contaminating cells. CM was obtained by culturing cortical astrocytes of newborn rats, as described previously, and removing the media on the fourth day. CM is enough to induce the formation of the characteristic paracellular seal of the blood-brain barrier in a culture of RBMECs.

The RBMECs were cultured in semipermeable millicell inserts with a pore size of 8 µm and an area of 1.2 cm^2. After reaching confluency, monolayers were supplemented in the apical compartment with 12.5 mg/ml 8-(4-Chlorophenylthiol) adenosine 39, 59-cyclic monophosphate sodium salt, 9.75 mg/ml the c-AMP phosphodiesterase-4-specific inhibitor RO-20-1724 and 50 mg/ml hydrocortisone, and CM in basal compartment. The inserts were placed into cells of the CellZcope® instrument (nanoAnalytics, Münster, Germany) and TEER was measured continuously. All peptide treatments (TP*, and P10) were administered after reaching a TEER around 100 $\Omega\cdot cm^2$. For each treatment, samples from apical and basal compartments were taken at different times and analyzed by HPLC.

Quantification of TP* in conditioned cultured media: Cultured media samples obtained from apical and basal compartments of the in vitro BBB model were filtered using centrifugal filters with a cutoff of 3 kDa. A sample of 15 µL

of the filtered solution was injected in a Waters HPLC system with a photodiode array (PDA) UV-vis detector using an isocratic method (Phase A: 70% MilliQ Water with 0.1% TFA and Phase B: 30% Methanol) with an analytical column Zorbax Eclipse C18 (4.5 μm, 150 x 4.5 mm). TP* and its variants were quantified using a solution of peptide with known concentration as a reference. Recovery of TP* was reported as the percentage of TP* quantified in the sample in comparison with the concentration of TP* added. The identity of TP* and its degradation products was confirmed by analyzing the fraction collected in each retention time by mass spectrometry.

Sample preparation for CD and EPR. Lyophilized peptides or proteins were weighted using a microbalance (Metler-Toledo XP26) and were dissolved in the corresponding buffer at pH 7.4 (100 mM NEM for peptides and HSA and 20 mM MOPS for the rPrP) with 50% glycerol for peptides and HSA, and 20% for rPrP. Samples were prepared at different concentrations to improve their measurements. For copper selectivity, two types of experiments were performed: To assess copper selectivity, stock solutions containing a mixture of Cu^{2+}:Zn^{2+} in either a 1:1 or a 1:10 ratio were prepared and 1 equivalent (0.5 mM) was added to a solution of TP* and CD and EPR spectra were collected. In the other experiment, Cu^{2+}-TP* complex were prepared and then 1 or 10 equiv of Zn^{2+} were added to the copper complex, the CD and EPR spectra were collected. The solution of hCtr1(1–14), Aβ(4–16) and HSA were prepared at 0.5 mM, PrP(60–91) was prepared at 0.3 mM to form the high-occupancy modes and 0.8 mM for the low-occupancy mode. PrP(92–99), PrP(106–115 were prepared at a concentration of 0.5 mM and) and OP was prepared at a concentration of 1.0 mM. To prepare the Cu^{2+}-complexes, a stock of $CuSO_4.5H_2O$ at 25 mM was used. For each titration, a fresh stock solution (15–20 mM) of TP* was prepared.

Sample preparation for ESEEM experiments. Lyophilized peptides were weighted using a microbalance (Metler-Toledo XP26) and were dissolved in MOPS 20 mM with 25% glycerol for peptides and without glycerol for rPrP. Copper complexes were prepared at a concentration of 0.3-0.5 mM. To form the Cu^{2+}-PrP complexes, a stock of $CuCl_2$ at 9.8 mM was used. For each titration, a fresh stock solution (15–20 mM) of TP* was prepared.

Sample preparation for XAS experiments. Lyophilized peptides were weighted using a microbalance (Metler-Toledo XP26) and were dissolved in 100 mM NEM at pH = 7.4 without glycerol at concentrations in the range of 3.7–5.9 mM.

Circular Dichroism Spectroscopy (CD). CD spectra were acquired on a Jasco J-815 CD spectropolarimeter at room temperature, using a quartz cell with a 10 mm path length. Spectra were measured >250 nm (according to the absorption of the peptide) to 800 nm. Measurements every 2 nm with a scan speed of 200 nm/min and averaging two scans.

CW and pulsed electron paramagnetic resonance spectroscopy (EPR and ESEEM). Continuous wave EPR spectra were recorded on a EMX plus Bruker Spectrometer using an X-band cavity (ER 4102ST) and a variable temperature nitrogen system (ER4131VT). EPR spectra were acquired at a microwave frequency of ~9.4 GHz and modulation frequency was 100 kHz. Each EPR spectra was measured three times and the averaged was obtained. EPR spectra were simulated using the last version of EasySpin (5.2.36) software on MATLAB, using pepper function for continuous wave EPR in solid-state. Spin system was defined as a Cu nucleus with natural isotopic abundance, and a spin of 1/2 , which is coupled to nitrogen nuclei with natural isotopic abundance. All the spectra were simulated with the hybrid method and considering an anisotropic broadening (*g*-strain).

Electron spin echo-envelope modulation (ESEEM) EPR experiments were performed using the pulse sequence $\pi/2 - \tau - \pi/2 - \tau -$ echo at 18 K on a Bruker E580 pulsed X-band spectrometer at ~9.7 GHz with a Bruker MD-5 dielectric resonator. The

pulse frequency was set to the center of the resonator mode and the magnetic field was adjusted to the maximum of the Cu^{2+} signal in the echo-detected filed-swept spectrum. The $\pi/2$ pulse length was 8 ns and τ was 210 ns.T was varied in 16 ns steps for 1024 points starting at 12 ns. Then, the ESEEM time domain signal was background subtracted using an exponential decay, zero filled to 2048 points, and Fourier transformed to obtain the ESEEM spectra.

X-ray Absorption Spectroscopy (XAS): XAS at the Cu *K*-edge was performed at beamline 7–3 at Stanford Synchrotron Radiation Lightsource (SLAC) (USA), using the setup: Source 20-pole, 2-Tesla wiggles, 0.8 mrad beam with energy range (5400–32000 eV), which allows a resolution of $1x10^{-4}$ $\Delta E/E$ allowing for a spot size of 2x15 mm^2. A monochromator LN_2 cooled, Si(220), $\phi = 0^o$ & 90°, double-crystal, non-fixed exit slit. 30-element Ge solid-state detector (PIPS, Lytle, Canberra). Sample was held in a Oxford Helium cryostat, cooled either by closed-cycle cooled He gas loop (10–100 K) or by open-cycle liquid He dewar (4–200 K). Signal-to-noise ratios were improved by averaging of spectra collected on the only sample spots. Photodamage was slightly observed. XAS spectra were averaged after detector dead time and self-absorption correction and normalized to derive XANES spectra. The monochromator energy axis was calibrated using Cu foil as standard and the resulting spectra was an average of five to six scans per sample.

Recombinant Prion Protein Expression: The protein was expressed as previously described.[2] In brief, the *Mus musculus* PrP(23-230) construct cloned into the pJ414 vector (DNA 2.0) was transformed and expressed using Escherichia coli (*E. coli*) (BL21[DE3]; Invitrogen). The protein was then purified following established methods.[3] Bacteria were grown in M9 minimal media supplemented with $^{15}NH_4Cl$ (1 g/L) (Cambridge Isotopes) for uniformly ^{15}N-labeled protein. Cells were grown at 37 °C until an OD_{600} of 1.0 was reached, and then 1 mM Isopropyl ß-D-1-thiogalactopyranoside (IPTG) was added, and cells were grown at 25 °C overnight. The protein was extracted from inclusion bodies at room temperature with 8 M guanidinium chloride (GndHCl), 100 mM Tris, and 100 mM Sodium Acetate at pH 8. The soluble fraction was purified by Ni^{2+}-immobilized metal-ion chromatography (IMAC). Proteins were eluted from the column with 5 M GndHCl, 100 mM Tris, and 100 mM Sodium Acetate at pH 4.5 and brought to pH 8 with 6 M potassium hydroxide (KOH) and left at 4 °C for 2 days to oxidize the native disulfide bridge. Proteins were desalted into 50 mM potassium acetate buffer and purified on a C8 column with reverse-phase liquid chromatography. Proteins were lyophilized and stored at –70 °C until use.

Nuclear Magnetic Resonance Lyophilized uniformly labeled ^{15}N-rPrP was first fully solubilized in water and protein concentration was determined by the absorbance at 280 nm with the appropriate extinction coefficient (ε_{ox}= 63,495.00 $M^{-1}cm^{-1}$). All samples were made to 100 μM of ^{15}N-rPrP, additional peptides or $CuCl_2$ was then added, and samples were made to 25 mM 2-(N-morpholino) ethane sulfonic acid (MES) buffer with 10% of D_2O, and the pH was adjusted to 6.6 with 1 M hydrochloric acid or 1 M potassium hydroxide. Samples were loaded into a Shigemi NMR tube (BMS-005B; Wilmad Glass) and a ^1H- ^{15}N Heteronuclear Single Quantum Coherence (HSQC) spectrum was collected at 37 °C on an 800-MHz spectrometer (Bruker) at the University of California, Santa Cruz NMR Facility. The protein peaks were determined using previous assignments.[2] Data was processed and analyzed with POKY.[4] Intensity ratios (I/Io) were calculated by dividing the non-apo intensity (I) by the peak intensity of WT rPrP (I_O). The I/I_O ratios were then scaled for comparison. The weighted average chemical shifts (Δ) were calculated using the equation $\Delta = [\Delta\delta HN^2 + (0.17*\Delta\delta N^2)]^{1/2}$, where $\Delta\delta HN$ and $\Delta\delta N$ are the ligand–induced amide proton and nitrogen chemical shifts, respectively.

Culture of neuroblastoma cells: SK-N-SH cell line derived from human neuroblastoma (ATCC HTB-11) were maintained in Dulbecco´s Modified Eagle´s Medium/Nutrient Mixture F-12 Ham (D0697), supplemented with 10% fetal bovine serum

(FBS), 1 mM L-glutamine, and 100 U/mL penicillin/100μg streptomycin. Cell cultures were maintained at 37°C, in a 5% CO_2 atmosphere. Cells were used until the 10th passage in culture. For differentiation, cells were plated into poly-L-lysine-coated dishes at a density ranging from 6.0 x10^3 to 60 x 10^3 cells per cm^2 and grown as monolayer. 24 hours after plating, the cells were incubated with fresh media and 10 μM trans retinoic acid in ethanol. The medium was changed on alternate days, and cultures were allowed to differentiate for two weeks.

Copper and treatments with copper chelators: After differentiation, cells were incubated with copper or media alone for 30 minutes. Cells treated with copper and Aβ(4–16) or TP* were either treated with copper or media alone for 30 minutes, and then, chelators were added and incubated during 30 minutes before fixation and immunostaining or protein extraction protocols.

Protein extraction: After differentiation, cells were lysed with 100 μL of lysis buffer (50 mM Tris-Cl pH 8.0, 1 mM EDTA, 0.5% Triton, 150 mM. NaCl), supplemented with Complete Protease inhibitor. The pierce BCA Protein Assay Kit was used for the quantification of proteins from cell extracts in a spectrophotometer iMark Microplate Reader (Bio-Rad). Samples were processed inmediately or stored at –20 °C until use.

Immunoblotting assays: For immunoblotting analysis, 50 μg of protein was loaded onto variable percentage polyacrylamide gels for the detection of the proteins of interest. After electrophoresis, proteins were transferred onto PVDF membranes and were blocked with 5% skim milk. The used primary antibodies were: anti-PrP^C clone 8H4, purified from hybridoma cell culture (1:1000), anti-NMDAR2B antibody (1:500) membranes were incubated overnight at 4 °C and then were washed using Tris Buffered Saline with Tween (0.1%) 1X buffer (TBST) and incubated with the secondary antibodies: anti-mouse HRP (1:2000), or anti-rabbit HRP (1:2000) for one hour. The membranes were then washed and revealed using a Western Lightning Plus-ECL, following the manufacturer's instructions. The membranes were photo-documented using the FUSION SOLO S (Vilver) instrument. A buffer stripping containing, Tris-HCl 62.5 mM pH 6.8, mercaptoethanol 100mM, SDS 2% w/v, was used for stripping and detection of different proteins on the same membrane. Load controls were made treating stripped membranes with an anti-β-actin HRP conjugated (1:40000) for 20 minutes.

Immunofluorescence: Cells were growth and differentiated in poly-L-lys treated coverslips. Cells were fixed using paraformaldehyde 4% in PBS pH 7.4. To determine the presence of extracellular proteins in the membrane, cells were processed without permeabilization treatment. Cells were washed between each step with phosphate-buffered saline (PBS), and 1% Bovine Serum Albumin IgG-Free. Blocking solution was added for 1 h at room temperature. After blocking, primary antibodies against the proteins of interest were added overnight at 4 °C, namely, anti-PrP^C clone 8H4, anti-GluN2B, or anti-GluN1. The next day, cells were washed with PBS, and secondary antibodies were subsequently added; For PrP^C primary antibody, Invitrogen Alexa-Fluor 594 Donkey anti-mouse was used, while for primary antibody GluN2B detection, Alexa Fluor 488 Donkey anti-rabbit antibody was employed. Subsequently, cells were washed and mounted with Vectashield with or without DAPI on slides for subsequent analysis. using a Leica TCS SP8 laser confocal microscope with a Leica HC PL APO CS2 63x/1.40 oil objective with a pinhole size of 1 AU, smart offset of 0.2%, and pixel dwell time of 600 ns. Four to six cells were analyzed using confocal microscopy. The images were processed using ImageJ® software (NIH) and analyzed using the Pearson correlation coefficient and line scan methods, which measures the relative fluorescence by means of the linear trace of an arrow through the distance in the different channels.

Statistical analysis: Statistical analyses were performed with the GraphPad Prism Version 10.2.1 software. All samples were tested for normality and using appropriate normality tests as indicated in the manuscript. Experiments were performed at least in triplicate (n = 4–6 per experimental condition).

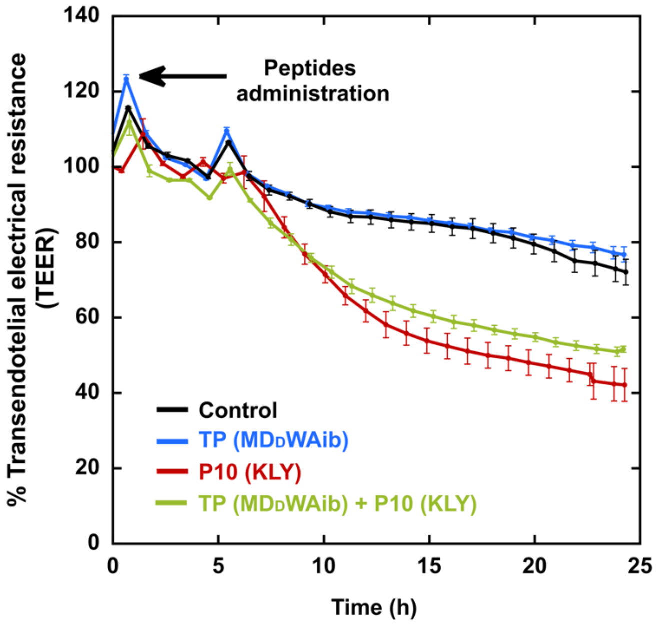

Figure S1. P10 opens the BBB. Administration of P10 alone (red trace) or together with TP (green trace) decreased the TEER of RBMECs in approximately 30% with respect to control monolayers (black). Treatment with TP alone (blue trace) exerted no effect on TEER.

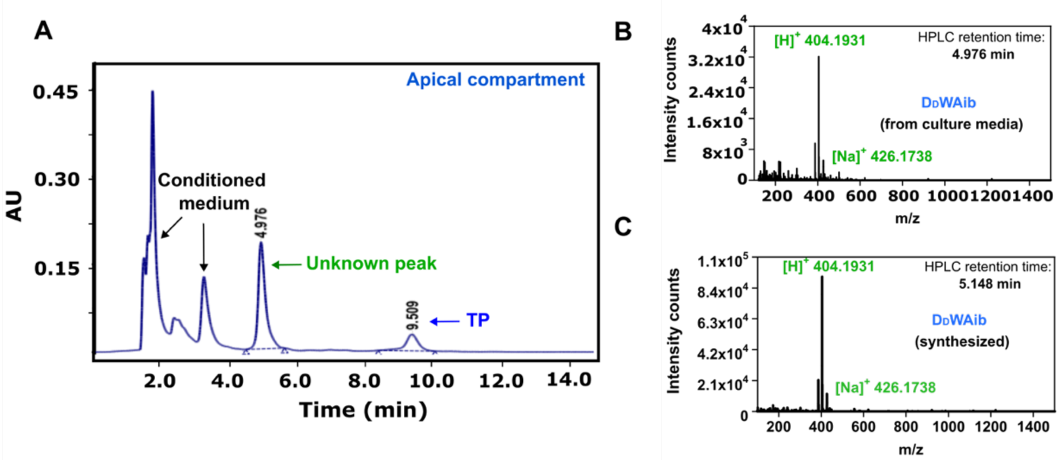

Figure S2. Chromatographic profile of TP samples incubated in the BBB model and identification of degradation products (A) In the region from one to four minutes, peaks corresponding to conditioned medium are observed. While TP elutes at 9.509 min, an unknown peak at 4.976 minutes was observed. (B) mass spectrum of the collected peak from the TP sample incubated in the BBB model. The m/z was assigned to the DDWAib sequence that was further confirmed with its corresponding synthesized and purified fragment (C).

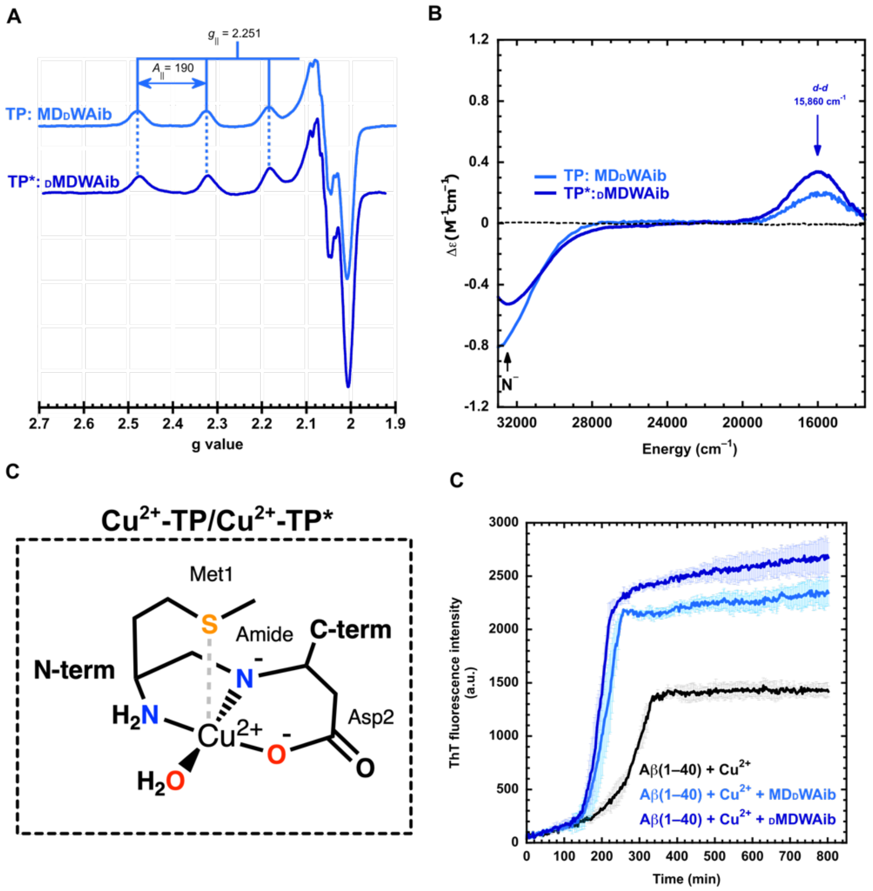

Figure S3. Bifunctional properties of original TP (MDᴅWAib) and optimized TP* (ᴅMDWAib). EPR (A), and CD (B) of TP* variants and its effect in the Cu^{2+}-induced Aβ(1–40) aggregation (D). Spectra of MDᴅWAib and ᴅMᴅWAib are shown in light blue and dark blue, respectively (A–B). Both peptides display similar EPR signals with $g_{||}$ = 2.251 and $A_{||}$ = 190x10^{-4} cm^{-1}, as well as CD features: a positive *d-d* band at 15,860 cm^{-1} and a negative LMCT at ~32,500 cm^{-1}. Hence, coordination spheres of Cu^{2+}-TP* and Cu^{2+}-TP* complexes are identical (C). Both peptides (blue traces) modulate Aβ(1–40) in the presence of copper in a similar fashion, as detected by ThT fluorescence (D).

Table S1. Synthesized peptides Tryptophan residues of peptide sequences are highlighted in blue

Peptide fragment	Sequence
PrP(60–91)	Ac-PHGGG**W**GQPHGGG**W**GQPHGGG**W**GQPHGGG**W**GQ-Am
PrP(92–99)	Ac-GGGTHSQ**W**-Am
PrP(106–115)	Ac-KTNMKHMAGA-Am
OP	Ac-PHGGG**W**GQ-Am
hCtr1(1–14)	NH_2-MDHSHHMGMSYMDS-Am
Aβ(4–16)	NH_2-EFRHDSGYEVHHQK-Am
TP* (ᴅTrp)	NH_2-MD**ᴅW**Aib-Am
TP** (ᴅMetᴅTrp)	NH_2-ᴅMD**ᴅW**Aib-Am
TP* (ᴅMet)	NH_2-ᴅMD**W**Aib-Am
P10	NH_2-KLY

Ac: Acetylation; Am: Amidation

Table S2. EPR parameters for the titration of Cu^{2+}-hCtr1 and Cu^{2+}-HSA complexes with TP*

Sample	$g_{\|}$	$A_{\|}$ (1×10^{-4} cm^{-1})
Cu^{2+}-hCtr1(1–14)	2.188	206
hCtr1(1-14) + 1.0 equiv. TP*	2.183	213
Cu^{2+}-HSA	2.188	205
Cu^{2+}-HSA + 1.0 equiv. TP*	2.187	210

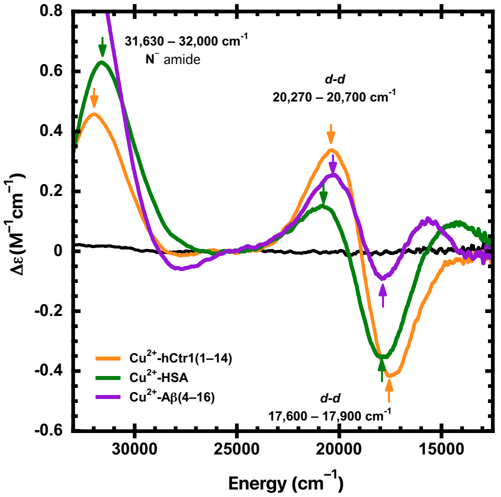

Figure S4. Comparative CD spectra from Cu^{2+}-ATCUN sites. CD spectra of Cu^{2+}-hCtr1(1–14) (orange), Cu^{2+}-HSA (green), Cu^{2+}-Aβ(4–16) (purple) complexes. The three complexes display similar ligand-field and LMCT transitions energies, as enlisted in Table S3.

Table S3. CD parameters of control Cu^{2+}-ATCUN sites of hCtr1(1–14), HSA, and Aβ(4–16)

Sample	***d-d*** **Ligand-field transitions**	**LMCT** **N^- amide**
Cu^{2+}-hCtr1(1–14)	– 17,600 cm^{-1}<br>+ 20,457cm^{-1}	+ 32,000 cm^{-1}
Cu^{2+}-HSA	–17,900 cm^{-1}<br>+20,700 cm^{-1}	+ 31,643 cm^{-1}
Cu^{2+}-Aβ(4–16)	–17,862 cm^{-1}<br>+20,270 cm^{-1}	+ 31,630 cm^{-1}

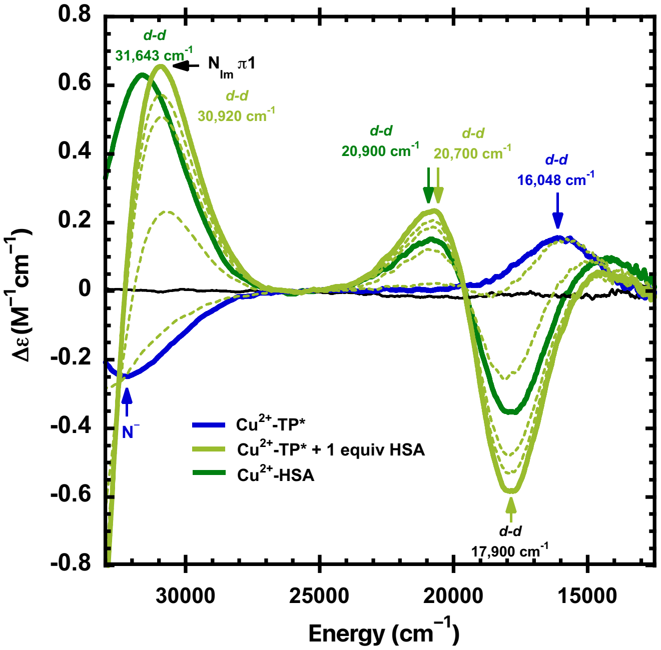

Figure S5. Titration of Cu^{2+}-TP* complex with HSA followed by CD: Cu^{2+}-TP* complex was titrated with HSA (green). Increasing amounts of HSA removed Cu^{2+} from the Cu^{2+}-TP* complex. The resulting spectra at the final point of the titration (solid light shade of colors) is very similar to that of their respective Cu^{2+}-ATCUN controls (solid dark green).

Table S4. CD parameters for the final point of the titrations of Cu^{2+}-TP* with ATCUN species.

Sample	***d-d*** **Ligand-field transitions**	**LMCT N⁻ amide**
Cu^{2+}-hCtr1(1–14)	–17,650 cm^{-1}<br>+20,520cm^{-1}	+ 32,000 cm^{-1}
Cu^{2+}-HSA	–17,900 cm^{-1}<br>+20,900 cm^{-1}	+ 31,643 cm^{-1}

Competition for Cu^{2+} between TP* and the non-OR sites of the PrP.

To analyze how TP impacts Cu^{2+} binding to the non-OR sites of PrP, the His96 and His111 sites were modelled using the PrP(92–99) and PrP(106–115) peptides. His96 and His111 binds Cu^{2+} at the non-OR region displaying almost identical Cu^{2+}-binding sites encompassing a mixture of 3N1O and 4N coordination modes with small differences in dissociation constants (C and F).[5-7] Peptide models were titrated with TP and followed by EPR and CD at pH = 7.4. The Cu^{2+}-PrP(92–99) complex yields characteristic EPR signals with $g_{\parallel}$ = 2.231 and $A_{\parallel}$ = 182 × 10^{-4} cm^{-1} (A, bold pink line), and by CD, two ligand-field transitions (– 16,800 cm^{-1} and + 19,940 cm^{-1}), and two LMCT bands (+ 28,200 cm^{-1} and – 32,450 cm^{-1}) assigned to $N_{im}\pi 1$ and N^{-}amide to copper CT transitions, respectively (B, bold pink spectrum). These spectroscopic features are associated to the 4N and 3N1O equatorial coordination modes shown in C, which are present in a mixture at pH=7.4.[5] Upon titration with TP, the EPR signals in the parallel region shift to larger *g* values and broaden significantly (A), indicating the presence of several species, while the CD signals of the Cu^{2+}-PrP(92–99) complexes decrease significantly, reaching ~40% of the original intensity at 1 equiv of TP (with respect to the metal ion) (B, solid lilac spectrum). Further additions of TP (up to 2 equiv) resulted in less broad EPR signals that allow the resolution of two sets of signals: one that corresponds to the Cu^{2+}-TP complex and a second species with $g_{\parallel}$ = 2.234 and $A_{\parallel}$ = 198 × 10^{-4} cm^{-1} (A, solid brown spectrum); the latter are different to those of the Cu^{2+}-PrP(92–99) and Cu^{2+}-TP complexes, suggesting the formation of a ternary species. Indeed, the second derivative of the perpendicular region shows a distinct nitrogen superhyperfine splitting pattern (A, inset) that resembles that of the ternary TP-Cu^{2+}-PrP(60–91) complex (4A, inset). Consistently, by CD the ligand-field transitions associated with the Cu^{2+}-PrP(92–99) complex disappear, a new negative *d-d* band at 12,500 cm^{-1} and a positive one at 16,270 cm^{-1} appear, while the negative LMCT at ~32,400 cm^{-1} assigned as an N^{-}_{amide} to copper CT persists (B, solid brown spectra). The resulting CD spectrum after the addition of 2 equiv of TP does not correspond to those of the Cu^{2+}-PrP(92–99) and displays the characteristic shifted ligand-field transitions observed for the ternary TP-Cu^{2+}-PrP(60–91) complex (C), suggesting the formation of a ternary TP-Cu^{2+}-PrP(92–99) species. In fact, the EPR spectrum after addition of 1 equiv of TP can be deconvoluted as a mixture of three species: 40% Cu^{2+}-PrP(92–99) complex, 30% Cu^{2+}-TP complex and 30% of a ternary TP-Cu^{2+}-PrP(92–99) complex assuming it has similar EPR features as those of the ternary Cu^{2+}-TP-PrP(60–91) complex (A, dotted spectrum). Finally, the EPR spectrum after addition of 2 equiv of TP can be simulated as a mixture of 20% of Cu^{2+}-TP and 80% of the ternary TP-Cu^{2+}-PrP(92–99) complex (A, dashed-black spectrum). Altogether, these results indicate that TP cannot effectively remove Cu^{2+} from the His96 binding site, and instead, it forms ternary species similar in nature to that observed with the OR sites.

A similar scenario is observed for the case of the His111 site. Upon titration of the Cu^{2+}-PrP(106–115) complex with TP, the characteristic signals (D-E purple spectra) of the 3N1O and 4N coordination modes,[6, 8] shown in F, decrease gradually, while the spectroscopic features associated with the Cu^{2+}-TP complex appear along with a new set of signals: EPR spectrum with $g_{\parallel}$ = 2.237 and $A_{\parallel}$ = 198 × 10^{-4} cm^{-1} (D, cerulean line) and a CD spectrum with a positive *d-d* band at 16,230 cm^{-1} and a negative LMCT at ~32,400 cm^{-1}(E, solid cerulean line). These spectroscopic features do not correspond to those of the Cu^{2+}-PrP(106–115), nor the Cu^{2+}-TP complexes, and in fact are very similar to those of the previously identified ternary TP-Cu^{2+}-PrP complexes. Moreover, the EPR spectrum after addition of 1 equiv of TP can be deconvoluted as a mixture of 30% Cu^{2+}-PrP(106–115) complex, 50% Cu^{2+}-TP complex and 20% of a ternary TP-Cu^{2+}-PrP(106–115) complex assuming it has similar EPR features as those of the ternary TP-Cu^{2+}-PrP(60-91) complex (D, dotted spectrum); while the EPR spectrum after addition of 2 equiv of TP can be simulated as a mixture of 40% of Cu^{2+}-TP and 60% of the ternary TP-Cu^{2+}-PrP(106–115) complex (D, dashed-black spectrum). Overall, these results indicate that TP can also form a ternary species similar to those observed with the OR and the His96 sites; however, it can remove more effectively Cu^{2+} from the His111 binding site, as compared to the His96 or the OR sites. It is interesting to note that this does not follow a direct trend with respect to the relative Cu^{2+} binding affinities of these sites (K_d ~0.46 μM for the His111 4N mode and K_d ~0.74 μM for the His96 4N mode),[7] suggesting that there are other factors that determine the relative stability of the observed ternary TP-Cu^{2+}-PrP species.

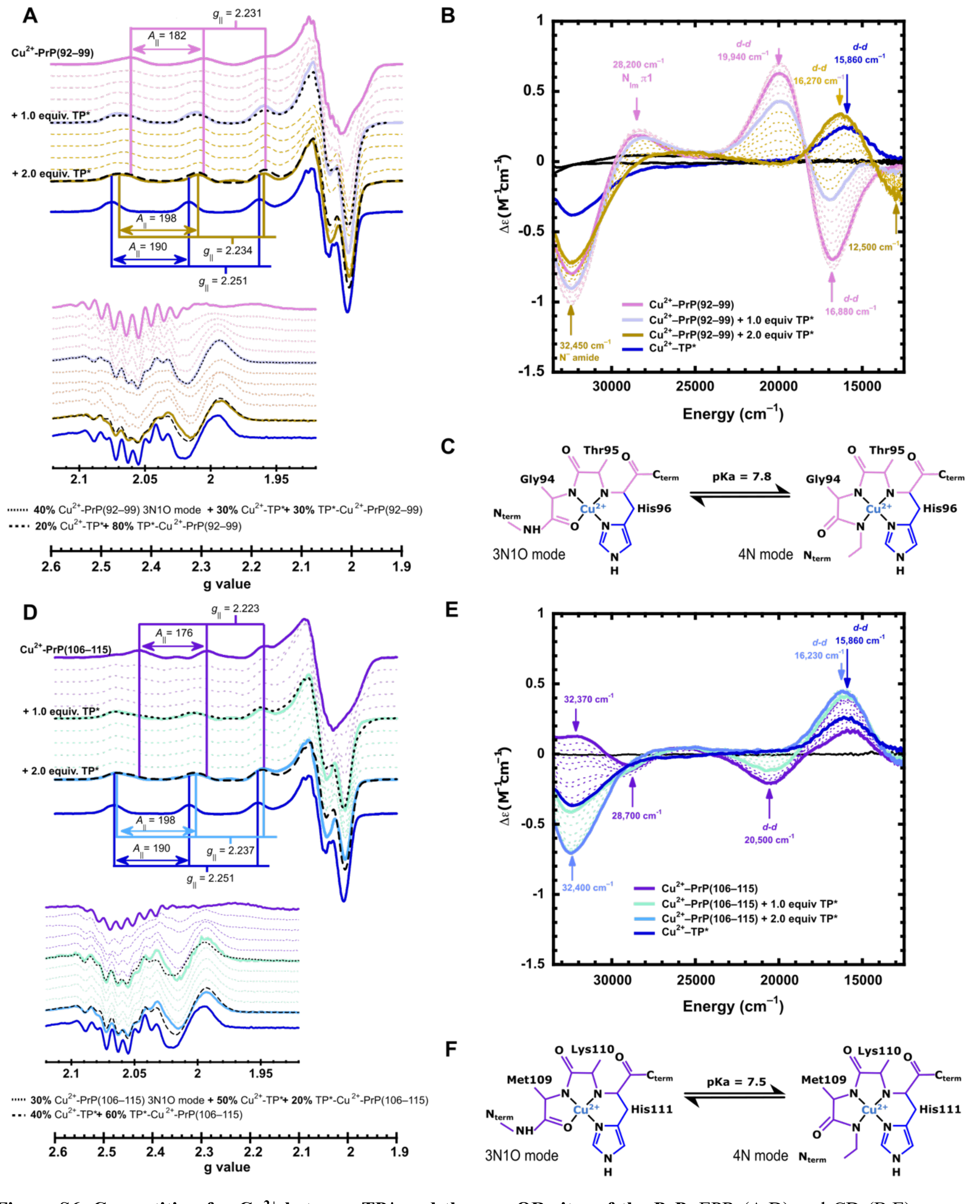

Figure S6. Competition for Cu^{2+} between TP* and the non-OR sites of the PrP. EPR (A,D) and CD (B,E) spectra following the titration of the PrP His96 and His111 sites with TP. The Cu^{2+}-PrP(92–99) and Cu^{2+}-PrP(106–115) complexes are represented in pink (A,B,C) and purple, (D,E,F), respectively. Increasing additions of TP are represented as colored dotted spectra, while those corresponding to the addition of 1.0 and 2.0 equivalents are represented in solid lines: lilac and brown for the His96 site, and green and cerulean for His111. The spectra after addition of 1.0 and 2.0 equivalents of TP were deconvoluted as a mixture of species (as indicated in the figure inset) and shown in black-dotted and black-dashed lines, respectively. Coordination modes for the Cu^{2+}-PrP(92–99) and Cu^{2+}-PrP(106–115) complexes are included in C and F, and all the $A_{\parallel}$ values are given in 1×10^{-4} cm^{-1}.

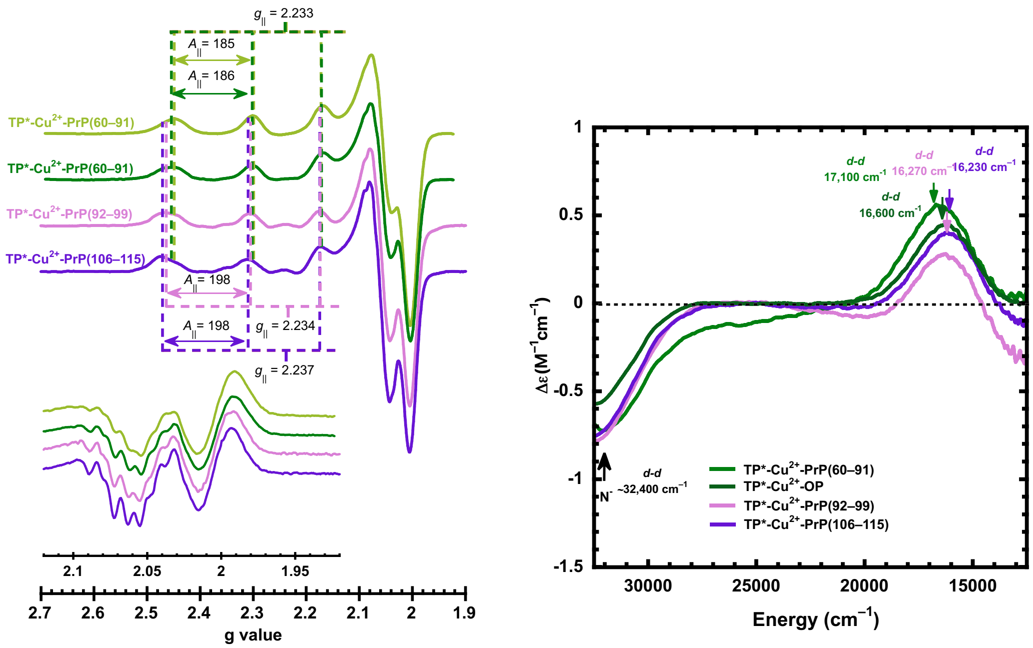

Figure S7. Comparative EPR and CD spectra of reported ternary TP*-Cu^{2+}-PrP complexes. Reported ternary TP*-Cu^{2+}-PrP complexes, namely TP*-Cu^{2+}-PrP(60–91) (low-occupancy modes, LOM) (light green spectra), TP*-Cu^{2+}-PrP(60–91) (high-occupancy modes, HOM) (green spectra), TP*-Cu^{2+}-OP (darkest green spectra), TP*-Cu^{2+}-PrP(92–99) (pink spectra), and TP*-Cu^{2+}-PrP(106–115) (purple spectra) display similar EPR (left) and CD (right) features, as enlisted in tables S5 and S6.

Table S5. EPR parameters for reported ternary TP*-Cu^{2+}-PrP complexes.

Ternary species	$g_{\|}$	$A_{\|}$ (1×10^{-4} cm^{-1})
TP*-Cu^{2+}-PrP(60–91) LOM	2.233	186
TP*-Cu^{2+}-PrP(60–91) HOM	2.233	185
TP*-Cu^{2+}-OP	2.237	184
TP*-Cu^{2+}-PrP(92–99)	2.234	198
TP*-Cu^{2+}-PrP(106–115)	2.237	198

Table S6. CD parameters for reported ternary TP*-Cu^{2+}-PrP complexes.

Ternary species	*d-d* Ligand-field	LMCT N^- amide
TP*-Cu^{2+}-PrP(60–91)	+ 17,100 cm^{-1}	– 32, 200 cm^{-1}
TP*-Cu^{2+}-PrP(92–99)	+ 16,270 cm^{-1}	–32,400 cm^{-1}
TP*-Cu^{2+}-PrP(106–115)	+ 16,230 cm^{-1}	– 32,400 cm^{-1}

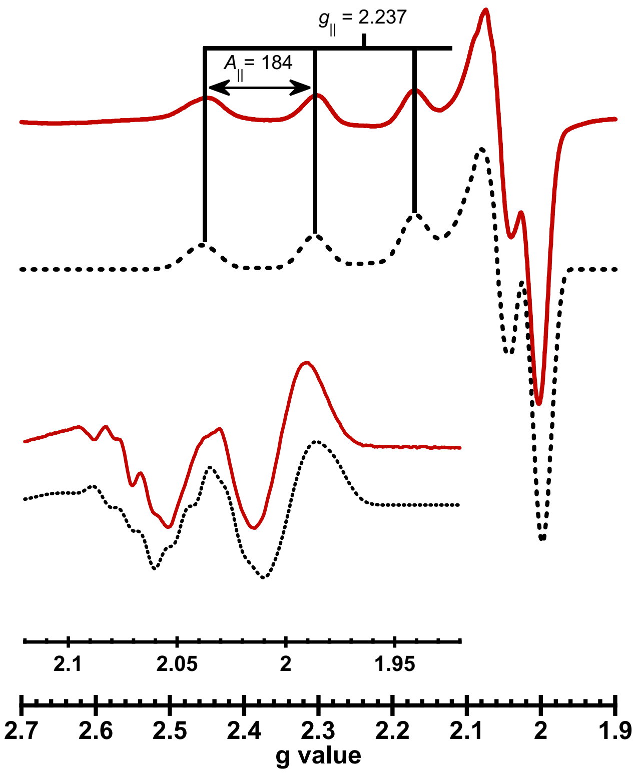

Figure S8. EPR simulation of the ternary TP*-Cu^{2+}-OP complex. Simulations of the ternary TP*-Cu^{2+}-OP complex and its second derivative were performed using EasySpin software (version 5.2.36), using the parameters enlisted in table S6.

Table S7. Spin Hamiltonian Parameters for the EPR spectrum simulation of the ternary TP*-Cu^{2+}-OP complex.

Parameter	value
g_x	2.056
g_y	2.057
g_z	2.237
${}^{Cu}A_x$	51
${}^{Cu}A_y$	26
${}^{Cu}A_z$	551
${}^{N1(NH2)}A_X$	40
${}^{N1(NH2)}A_y$	35
${}^{N1(NH2)}A_z$	35
${}^{N2(amide)}A_x$	45
${}^{N2(amide)}A_y$	50
${}^{N2(amide)}A_z$	45
${}^{N3(His)}A_x$	39
${}^{N3(His)}A_y$	45
${}^{N3(His)}A_z$	39
g strain$_x$	0.0135
g-strain$_y$	0.0120
g-strain$_z$	0.0210

Note: All hyperfine coupling values are given in MHz

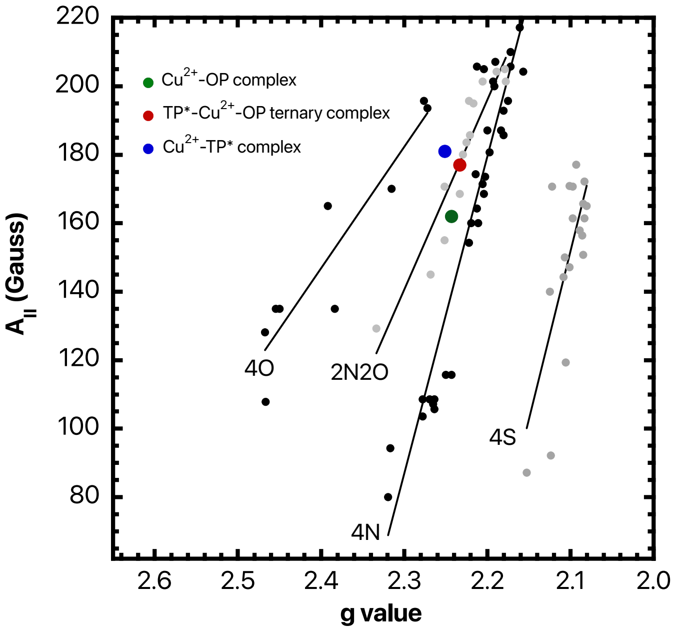

Figure S9. Peisach-Blumberg correlations for the Cu^{2+}-OP, TP*-Cu^{2+}-OP, and Cu^{2+}-TP* complexes. Peisach-Blumberg correlations for A_z and g_z values for TP*-Cu^{2+}-OP (green), ternary TP*-Cu^{2+}-OP (red) and TP*-Cu^{2+} (blue) complexes with different equatorial coordination environments: 4S, 4O, 4N or 2N2O, using data from ref [9]. Ternary TP*-Cu^{2+}-OP complex falls closer to the 4N correlation as compared with the Cu^{2+}-TP* complex, which suggests that ternary complex contains more contribution from nitrogen-based ligands.

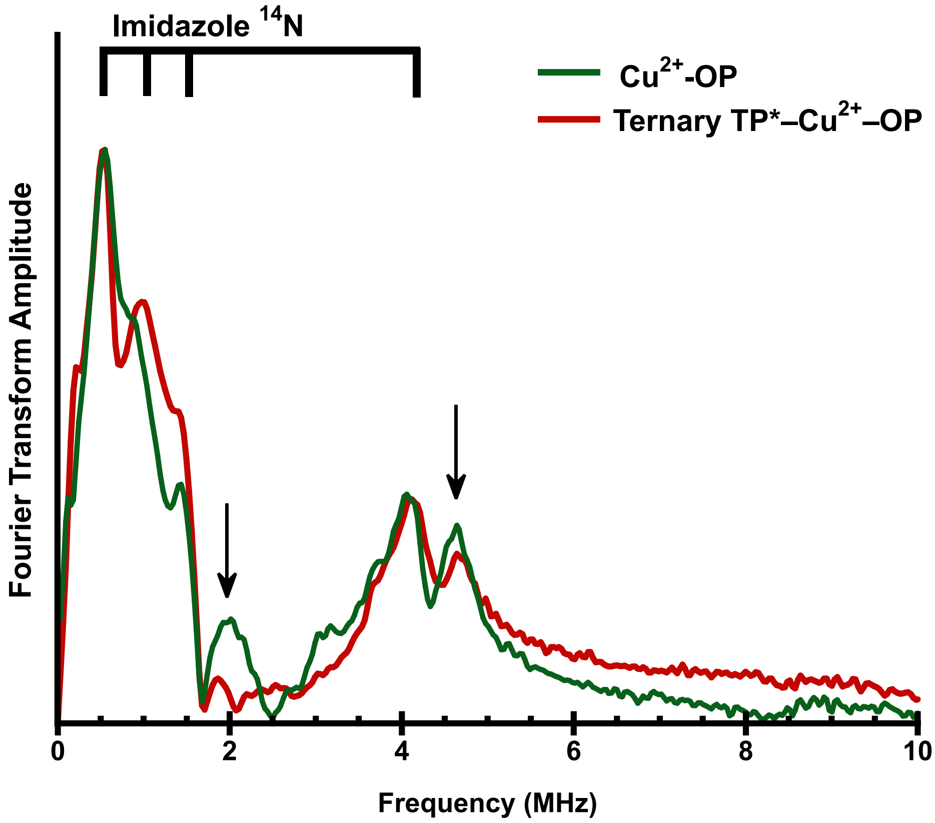

Figure S10. ESEEM comparison between Cu^{2+}-OP and ternary TP*-Cu^{2+}-OP spectra. Comparison of the normalized intensity of Cu^{2+}-OP (green) and ternary TP*-Cu^{2+}-OP (red). The DQ feature at 4.11 MHz and the peak at 2 MHz are assigned to the OP backbone coordination to Cu^{2+}.[10, 11] While Cu^{2+}-OP complex display these evident features, both signals are clearly decreased in the ternary TP*-Cu^{2+}-OP complex. This evidence strongly discards the participation of the OP backbone to Cu^{2+} in the coordination sphere of the ternary complex, while the participation of the His residues is clearly observed in the NQI signals.

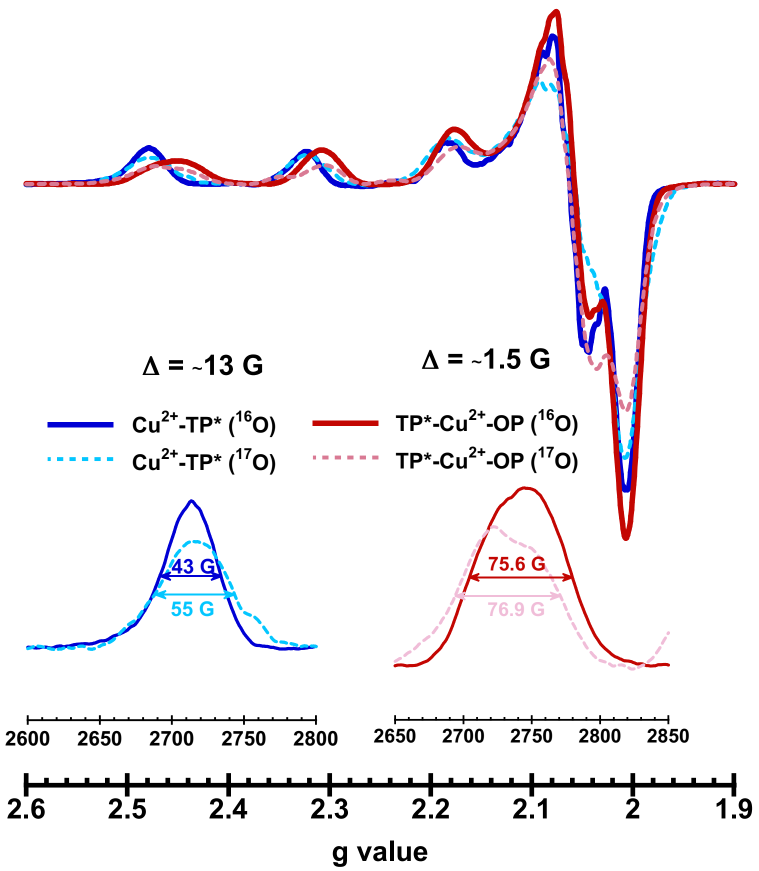

Figure S11. Comparative EPR spectra of the Cu^{2+}-TP* and ternary TP*-Cu^{2+}-OP complexes prepared in ^{16}O water and ^{17}O enriched water. Comparative X-band EPR spectra of Cu^{2+}-TP* (blue) and TP*-Cu^{2+}-OP (maroon) complexes in buffer at pH = 7.4, prepared with either ^{16}O water (solid lines) and ^{17}O enriched water (dashed lines). Inset displays a broadening of the first parallel signal, indicating the linewidths at half the maximal amplitude

Table S8. EPR parameters for the TP*-Cu^{2+} complex prepared in ^{16}O water and ^{17}O enriched water.

**Sample**	**TP*-08Cu^{2+} $H_2{}^{16}O$**	**TP*-08Cu^{2+} $H_2{}^{17}O$**	***Signal broadening***
Linewidth 2713 G	43 G	55 G	55 G – 43 G = **12 G = 13 X 10^{-4} cm^{-1}**
Linewidth 2891 G	51 G	55 G	55 G – 41G = **14 G = 15 X 10^{-4} cm^{-1}**
g_{ll}	2.257	2.249	
A_{ll}	192	192	

Table S9. EPR parameters for the TP*-Cu^{2+}-OP ternary complex prepared in ^{16}O water and ^{17}O enriched water.

**Sample**	**TP*-Cu^{2+}-OP $H_2{}^{16}O$**	**TP*-Cu^{2+}-OP $H_2{}^{17}O$**	***Signal broadening***
Linewidth 2713 G	76 G	77 G	77 G – 76 G = **1 G = 1 X 10^{-4} cm^{-1}**
Linewidth 2891 G	54 G	52 G	54 G – 52G = **2 G = 2 X 10^{-4} cm^{-1}**
g_{ll}	2.236	2.238	
A_{ll}	181	184	

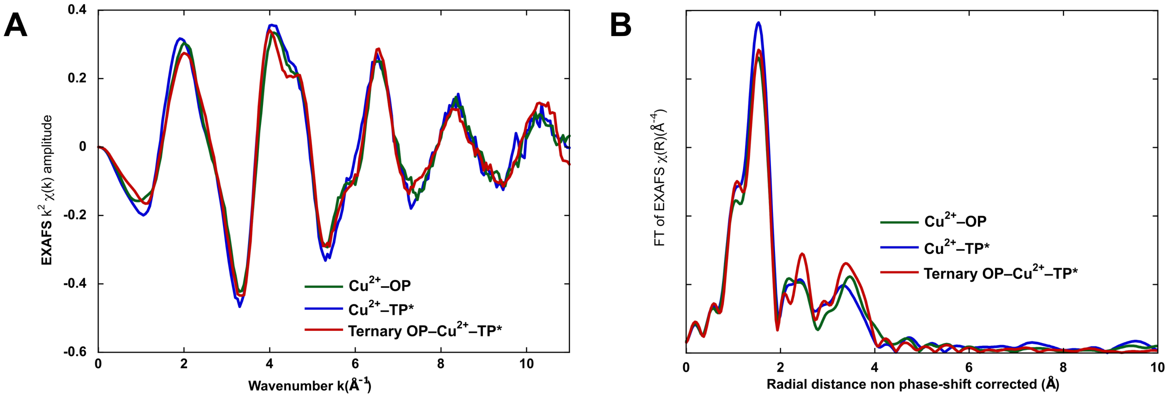

Figure S12. Comparison of EXAFS and FT of EXAFS spectra of Cu^{2+}-OP, Cu^{2+}-TP* and TP*-Cu^{2+}-OP complexes. Comparison of EXAFS scattering patterns of the three Cu^{2+} complexes (Cu^{2+}-OP green, Cu^{2+}-TP* blue, and ternary TP*-Cu^{2+}-OP red.) are shown in (A). The three complexes display similar interference patterns; however, slight changes can be observed at short distances from scattering neighbors to the metal center (~1.9 Å, 2.2 Å, and 3.8 Å) (B). Taken together, spectroscopic data confirm that the three complexes share similar geometry, but discrete changes in their coordination spheres.

Table S10. EXAFS simulation parameters using a 3N1O1S coordination mode for the TP*-Cu^{2+}-OP complex. Numbers in brackets are the estimated uncertainties on the last digit. σ2 is the Debye Waller factor of the considered scattering path. $S0^2$ is the global amplitude factor, e0 is the energy threshold, R_{factor} is the agreement factor of the fit in %. Note that N+O=4 (fixed). Numbers in italics have been fixed.

Path	N	σ^2	R
Cu-N	3.2(6)	0.0015	2.00(1)
Cu-O	0.8	*0.0015*	2.20(3)
Cu-S	*1*	0.0058	2.67(2)
Cu…C	3.9(7)	0.0267	3.26(3)
Cu…C-O	*3.9*	0.0056	4.15(2)

$S0^2$ = 1.1, e0 = 0.18 eV, R_{factor} = 2.1%

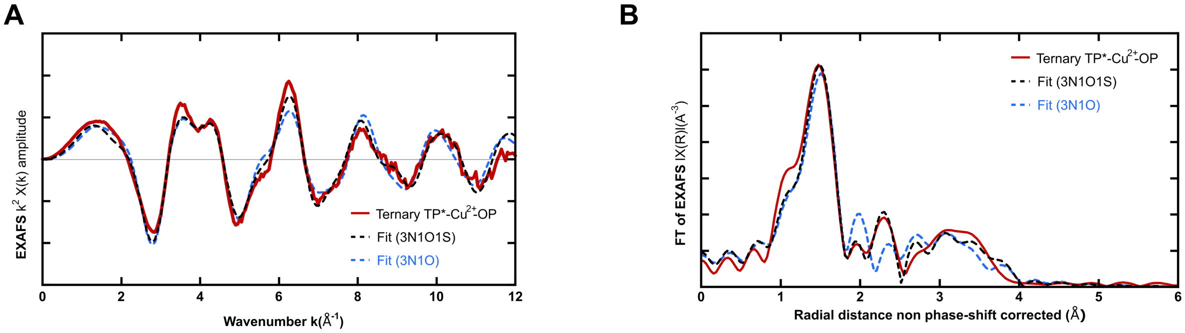

Figure S13. EXAFS fit of ternary TP*-Cu^{2+}-OP complex using a 3N1O coordination sphere. Fitting of experimental EXAFS spectrum (maroon) and its FT for the TP*-Cu^{2+}-OP complex was performed considering a 3N1O1S (black dashed lines) and a 3N1O (blue dashed lines) coordination shell. The coordination sphere 3N1O1S yields a significantly better fit as compared to that of a 3N1O shell. Simulation parameters are given in table S10 for 3N1O1S and in table S11 for 3N1O coordination shells.

Table S11. EXAFS simulation parameters using a 3N1O coordination mode for the TP*-Cu^{2+}-OP complex. Numbers in brackets are the estimated uncertainties on the last digit. σ2 is the Debye Waller factor of the considered scattering path. $S0^2$ is the global amplitude factor, e0 is the energy threshold, R_{factor} is the agreement factor of the fit in %. Note that N+O=4 (fixed). For comparison with the fit of 3N1O1S (Table S10), the main parameters have been kept equal.

Path	N	σ^2	R
Cu-N	3.2(6)	0.0023	2.01(1)
Cu-O	0.8	*0.0023*	2.27(3)
Cu…C	3.9(7)	0.0226	3.13(3)
Cu…C-O	*3.9*	0.0056	4.14(2)

$S0^2 = 1.1$, e0 = 0.18 eV, $R_{factor} = 5.6\%$

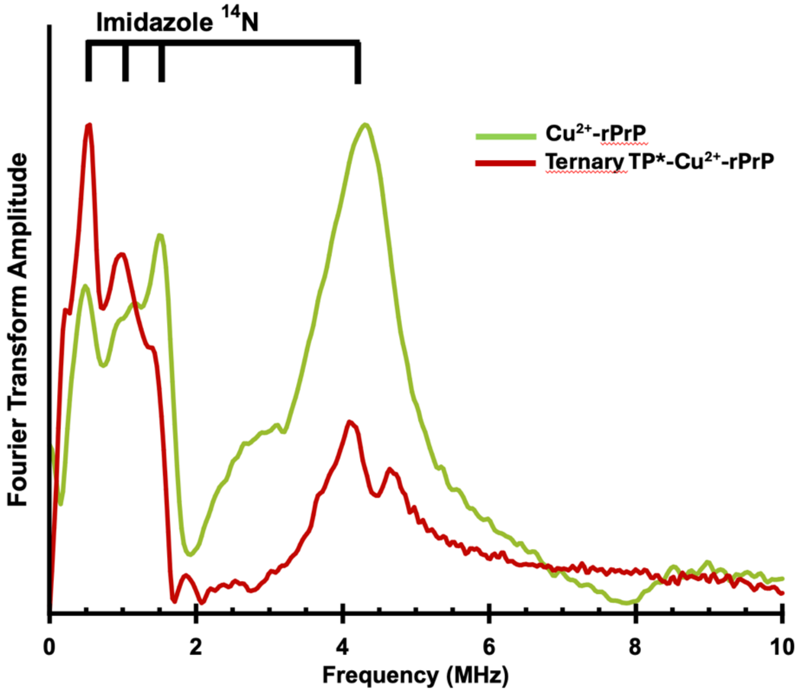

Figure S14. ESEEM comparison between Cu^{2+}-rPrP and ternary TP*-Cu^{2+}-OP spectra. Comparison of the normalized intensity of Cu^{2+}-rPrP (olive green) and ternary TP*-Cu^{2+}-OP complex (maroon). The DQ/NQI intensity ratio is related with the participation of His residues in the coordination sphere of Cu^{2+}-complexes. While Cu^{2+}-rPrP in a low-occupancy coordination mode display a high DQ/NQI ratio and a multi-His coordination sphere has been previously reported.[10, 11] The DQ/NQI ratio for the ternary TP*-Cu^{2+}-OP complex is clearly decreased, which is associated with the participation of a single His residue in its coordination sphere.

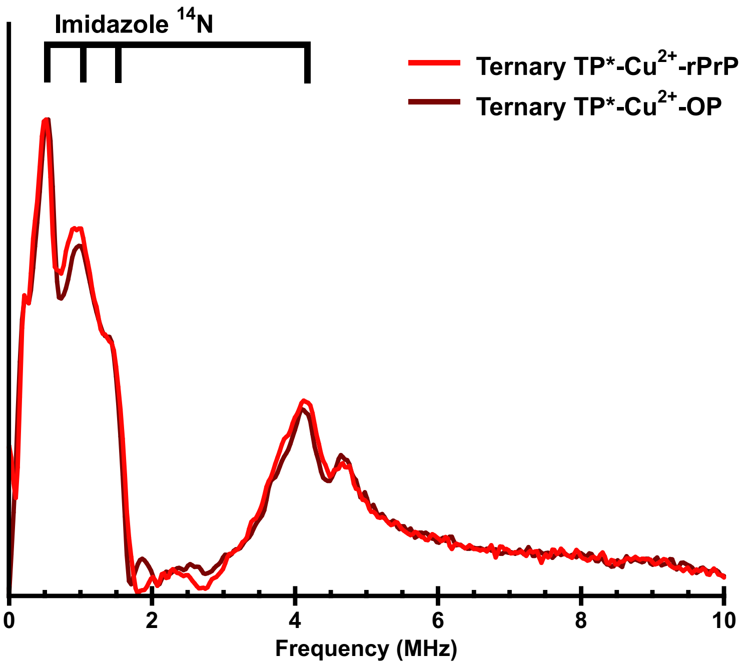

Figure S15. ESEEM comparison between ternary TP*-Cu^{2+}-rPrP and ternary TP*-Cu^{2+}-OP spectra. Comparison of the normalized intensity of TP*-Cu^{2+}-rPrP (red) and ternary TP*-Cu^{2+}-OP complex (maroon). Both spectra display very similar features, including a similar DQ/NQI ratio, which strongly suggest that only a His residue participates in the coordination sphere of both ternary complexes.

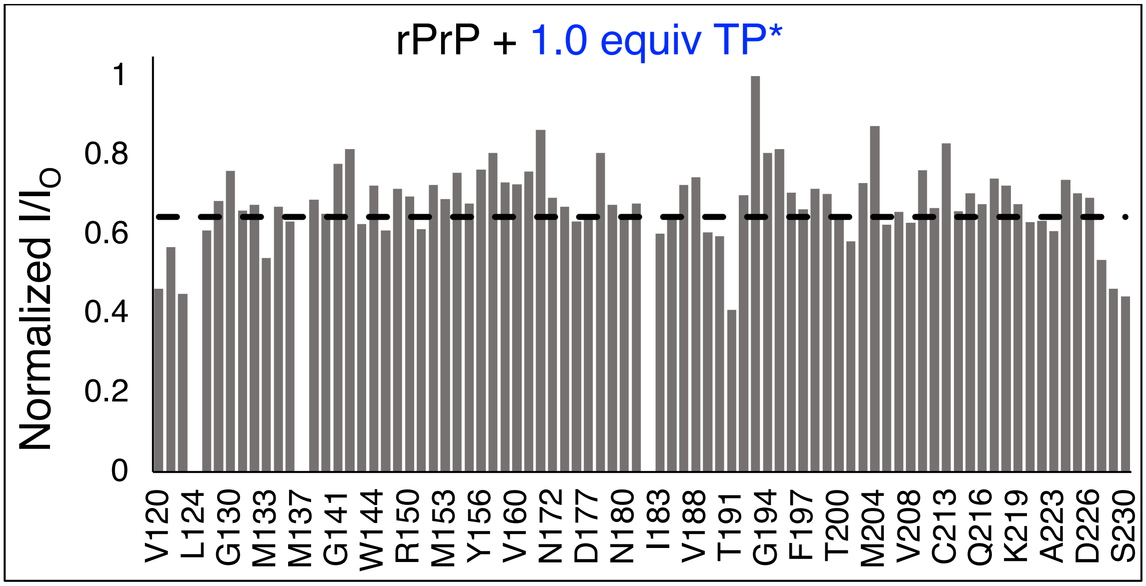

Figure S16. ^{1}H-^{15}N HSQC intensity ratio of rPrP + 1.0 equiv of TP* compared to rPrP. Each peak in the HSQC for rPrP with 1.0 equiv TP* were divided by rPrP peak intensity values. The ratios were then intensity normalized. Grey bars show peaks not significantly affected by the addition of TP*. Two peaks disappeared but no peak significantly changed, suggesting rPrP does not interact with TP* in the absence of Cu^{2+}.

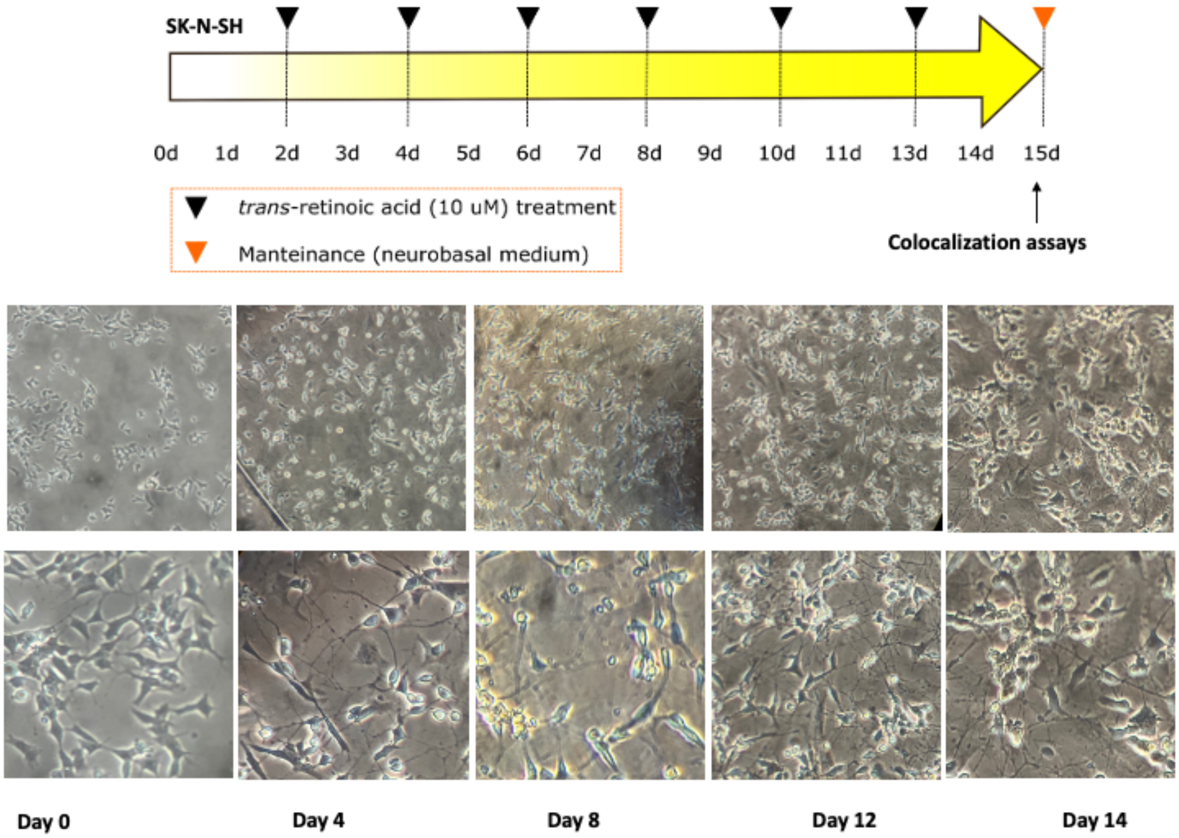

Figure S17. Differentiation of SK-N-SH cell line using RA. For differentiation, SK-N-SH cells were incubated with fresh media and 10 µM trans retinoic acid in ethanol. The medium was changed on alternate days, and cultures were allowed to differentiate for two weeks. Before differentiation (day 0), cells display short neurite processes (N-type cells). After treatments, most cells adopted neuronal morphology. Cells showed small phase-bright properties, with a high nucleus/cytoplasm ratio. Extensive neuritic networks were observed after day 8.

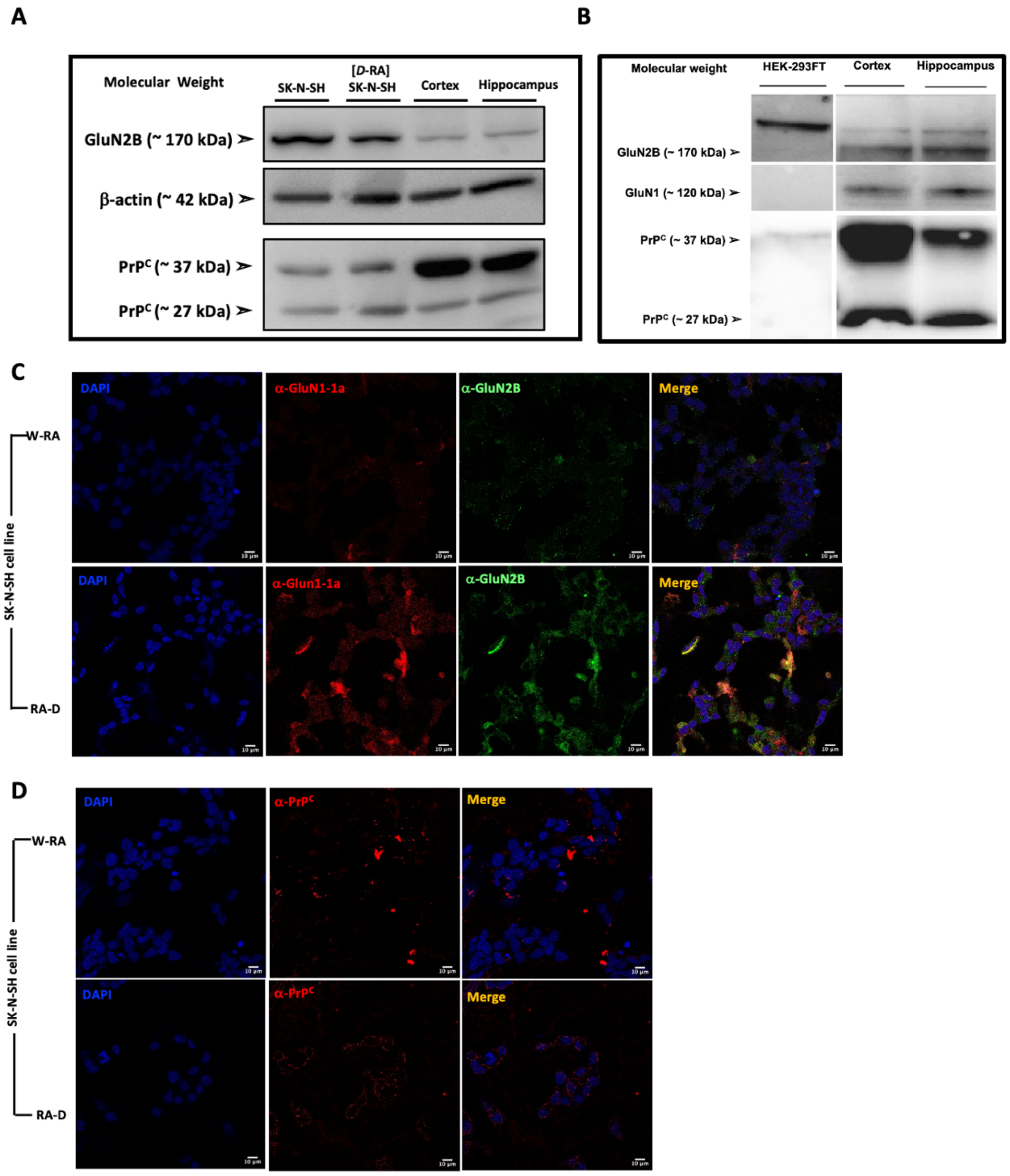

Figure S18. Expression of NMDAR (GluN1-1a/GluN2B subunits) and PrP^C in the RA-differentiated SK-N-SH cell line.

To validate the SK-N-SH cell line as a model to study the PrP^C-NMDAR colocalization, the expression of both proteins was investigated using western-Blot (A,B) and immunofluorescence (C,D) studies. The expression of NMDAR was evaluated by immunodetecting (A) the GluN2B subunit (~170 kDa), while PrP^C was also evaluated by the detection of ~37 and ~27 kDa band weights. (B) as negative control, HEK293-FT cells were employed to detect GluN2B, GluN1 and PrP^C proteins. The expression of those proteins in SK-N-SH and HEK293-FT cells was compared with rat cortex and hippocampus as positive expression controls, and the load control was performed detecting β-catenin protein (~ 42 kDa). The localization of the proteins were analyzed using immunofluorescence of non-permeabilized SK-N-SH cells (C,D). While expression of NMDAR (C) increased after the differentiation protocol (RA-D, first row) as compared to those without differentiation treatment (W-RA); the localization of PrP^C (D) became more homogeneous in the peripheric of cell membranes (RA-D) as compared with the untreated cells (W-RA).

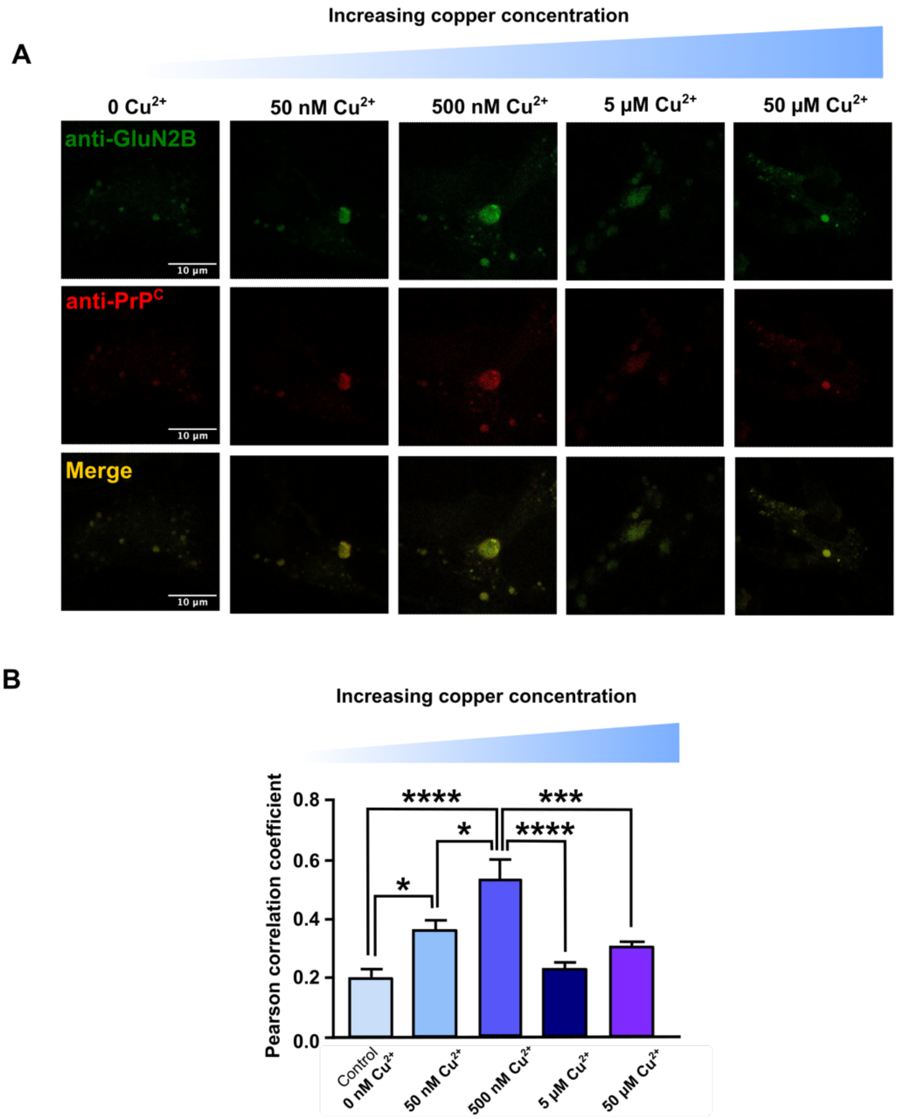

Figure S19. Effect of Cu^{2+} in the colocalization of PrP^C and NMDAR. (A) Representative confocal images of colocalization of GluN2B (green) and PrP^C (red) using increasing concentrations of Cu^{2+} ions. SK-N-SH cells were incubated in DMEM/F12 media alone, or 50 nM Cu^{2+}, 500 nM Cu^{2+}, 5 mM Cu^{2+}, or 50 mM Cu^{2+}. (B) Colocalization of GluN2B and PrP^C was quantified using the Pearson correlation coefficient (PCC) (data is shown with the mean +/- SEM; one-way ANOVA, post hoc Tukey; *P<0.05, ***P<0.001,****P<0.0001).

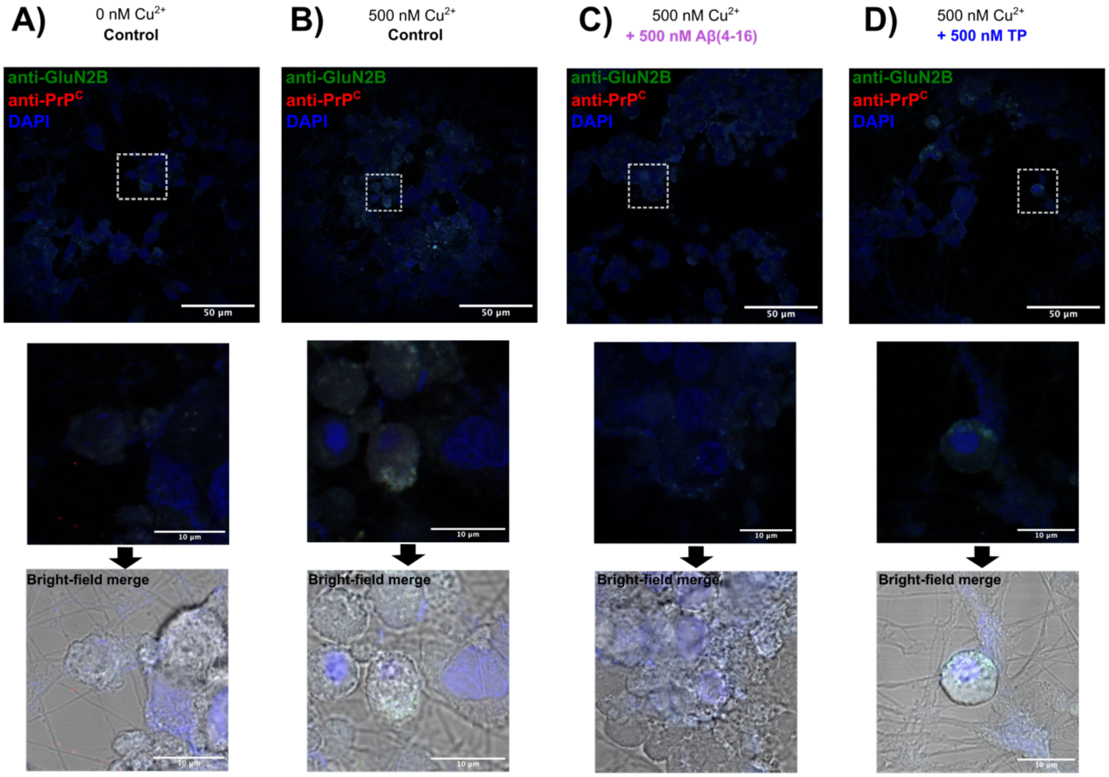

Figure S20. Colocalization of GluN2B and PrP^C in SK-N-SH cells using DAPI-containing mounting media. Representative images of colocalization between GluN2B and PrP^C in conditions of A) no copper; B) 500 nM Cu^{2+}; or 500 nM Cu^{2+} and C) Aβ(4–16) or D) 500 nM TP*. Each condition includes a maximization inset and its corresponding bright-field merge.

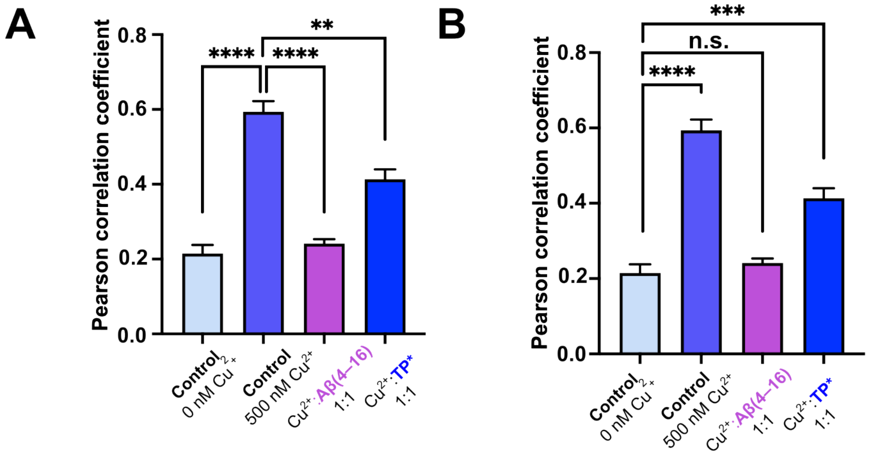

Figure S21. Multiple comparisons of the effect of TP* in the Cu^{2+}-dependent colocalization of PrP^C and NMDAR. Colocalization between GluN2B and PrP^C was quantified using PCC. Comparison of PCC values of the different experimental conditions with those of copper control (A) or compared to control without copper (C). (In both cases, data are the means +/– SEM, one way ANOVA, followed by Dunnett´s test; *P<0.05, ***P<0.001, ****P<0.0001).

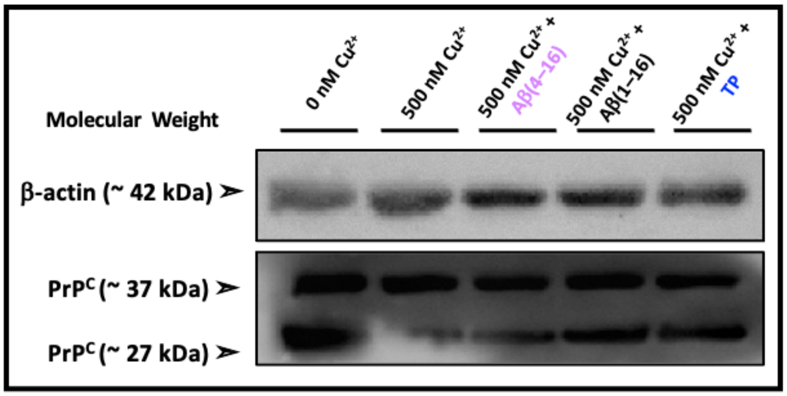

Figure S22. Expression of PrP^C in the different experimental condition. Comparative expression patterns of PrP^C with the different treatments used in colocalization assays. The signals in the immunoblotting does not display apparent changes in the expression of PrPC (~37 and ~27 kDa) as compared with the expression of β-actin protein (~ 42 kDa), a protein with relatively stable expression under different experimental conditions.

References

(1) Jensen, K. J.; Shelton, P. T.; Pedersen, S. L. Peptide Synthesis and Applications. In *Methods in Molecular Biology*, 2013.
(2) Evans, E. G.; Pushie, M. J.; Markham, K. A.; Lee, H. W.; Millhauser, G. L. Interaction between Prion Protein's Copper-Bound Octarepeat Domain and a Charged C-Terminal Pocket Suggests a Mechanism for N-Terminal Regulation. *Structure* **2016**, *24* (7), 1057-1067. DOI: 10.1016/j.str.2016.04.017 From NLM Medline.
(3) Spevacek, A. R.; Evans, E. G.; Miller, J. L.; Meyer, H. C.; Pelton, J. G.; Millhauser, G. L. Zinc drives a tertiary fold in the prion protein with familial disease mutation sites at the interface. *Structure* **2013**, *21* (2), 236-246. DOI: 10.1016/j.str.2012.12.002 From NLM Medline.
(4) Lee, W.; Rahimi, M.; Lee, Y.; Chiu, A. POKY: a software suite for multidimensional NMR and 3D structure calculation of biomolecules. *Bioinformatics* **2021**, *37* (18), 3041-3042. DOI: 10.1093/bioinformatics/btab180 From NLM Medline.
(5) Grande-Aztatzi, R.; Rivillas-Acevedo, L.; Quintanar, L.; Vela, A. Structural models for Cu(II) bound to the fragment 92-96 of the human prion protein. *J Phys Chem B* **2013**, *117* (3), 789-799. DOI: 10.1021/jp310000h From NLM Medline.
(6) Rivillas-Acevedo, L.; Grande-Aztatzi, R.; Lomeli, I.; Garcia, J. E.; Barrios, E.; Teloxa, S.; Vela, A.; Quintanar, L. Spectroscopic and electronic structure studies of copper(II) binding to His111 in the human prion protein fragment 106-115: evaluating the role of protons and methionine residues. *Inorg Chem* **2011**, *50* (5), 1956-1972. DOI: 10.1021/ic102381j From NLM Medline.
(7) Sánchez-López, C.; Rivillas-Acevedo, L.; Cruz-Vásquez, O.; Quintanar, L. Methionine 109 plays a key role in Cu(II) binding to His111 in the 92–115 fragment of the human prion protein. *Inorganica Chimica Acta* **2018**, *481*, 87-97. DOI: https://doi.org/10.1016/j.ica.2017.09.046.
(8) Klewpatinond, M.; Davies, P.; Bowen, S.; Brown, D. R.; Viles, J. H. Deconvoluting the Cu2+ binding modes of full-length prion protein. *J Biol Chem* **2008**, *283* (4), 1870-1881. DOI: 10.1074/jbc.M708472200 From NLM Medline.
(9) Peisach, J.; Blumberg, W. E. Structural implications derived from the analysis of electron paramagnetic resonance spectra of natural and artificial copper proteins. *Arch Biochem Biophys* **1974**, *165* (2), 691-708. DOI: 10.1016/0003-9861(74)90298-7 From NLM Medline.
(10) Burns, C. S.; Aronoff-Spencer, E.; Legname, G.; Prusiner, S. B.; Antholine, W. E.; Gerfen, G. J.; Peisach, J.; Millhauser, G. L. Copper coordination in the full-length, recombinant prion protein. *Biochemistry* **2003**, *42* (22), 6794-6803. DOI: 10.1021/bi027138+ From NLM Medline.
(11) Burns, C. S.; Aronoff-Spencer, E.; Dunham, C. M.; Lario, P.; Avdievich, N. I.; Antholine, W. E.; Olmstead, M. M.; Vrielink, A.; Gerfen, G. J.; Peisach, J.; et al. Molecular features of the copper binding sites in the octarepeat domain of the prion protein. *Biochemistry* **2002**, *41* (12), 3991-4001. DOI: 10.1021/bi011922x From NLM Medline.
